# MYC-Targeting Inhibitors Generated from a Stereodiversified Bicyclic Peptide Library

**DOI:** 10.1021/jacs.3c09615

**Published:** 2024-01-03

**Authors:** Zhonghan Li, Yi Huang, Ta I Hung, Jianan Sun, Desiree Aispuro, Boxi Chen, Nathan Guevara, Fei Ji, Xu Cong, Lingchao Zhu, Siwen Wang, Zhili Guo, Chia-en Chang, Min Xue

**Affiliations:** † Department of Chemistry, University of California, Riverside, Riverside, California 92521, United States; ‡ Environmental Toxicology Graduate Program, University of California, Riverside, Riverside, California 92521, United States

## Abstract

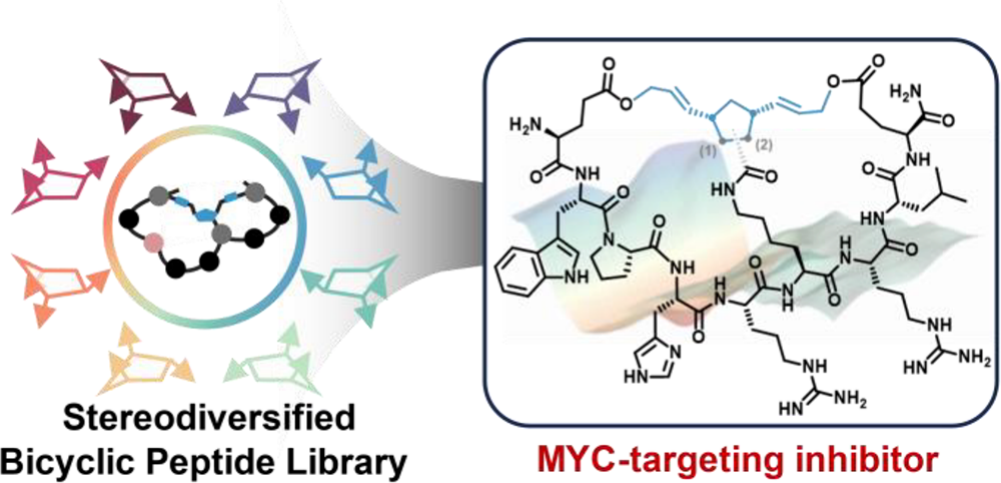

Here, we present the second generation of our bicyclic peptide library (NTB), featuring a stereodiversified structure and a simplified construction strategy. We utilized a tandem ring-opening metathesis and ring-closing metathesis reaction (ROM-RCM) to cyclize the linear peptide library in a single step, representing the first reported instance of this reaction being applied to the preparation of macrocyclic peptides. Moreover, the resulting bicyclic peptide can be easily linearized for MS/MS sequencing with a one-step deallylation process. We employed this library to screen against the E_363_-R_378_ epitope of MYC and identified several MYC-targeting bicyclic peptides. Subsequent in vitro cell studies demonstrated that one candidate, NT-B2R, effectively suppressed MYC transcription activities and cell proliferation.

## Introduction

MYC is a critical transcription factor whose aberrant activity is implicated in more than 75% of all human cancer cases.^1−3^ Numerous studies have established that MYC can facilitate cancer initiation, maintenance, and progression by boosting transcription activities.^2,4−6^ Although its exact mechanism of action is under debate,^7−11^ MYC has long been one of the most sought-after oncology drug targets.^12−14^ Despite decades of research, MYC remains an “undruggable” target with no clinically viable therapeutics.^5,12^

The challenges of targeting MYC manifest in multiple aspects. First, MYC is an intrinsically disordered protein with no discernible binding pockets, posing substantial obstacles for conventional small-molecule-based drug development pipelines.^12,15^ Although there exist a few putative sites amenable to small-molecule binding, medicinal chemistry optimization pathways are unclear.^16,17^ Second, MYC has fast turnover rates at both transcription and translation levels, with typical half-lives shorter than 30 min.^6,18,19^ This property confounds the efforts of covalent targeting strategies, as it may quickly exhaust the intracellular drug pool. Third, MYC exerts its function in the nuclear region,^15,20^ inaccessible to large biologics such as therapeutic antibodies. With these challenges, current research efforts have significantly shifted toward indirect approaches, that is, targeting MYC’s upstream regulators and downstream effectors.^21−26^ However, because MYC is a hub for many signaling pathways, a collection of indirect methods is needed to accommodate the diverse pathological mechanisms.^27^ Therefore, directly targeting MYC remains an attractive goal.

Previously, we demonstrated an MYC-targeting peptide, NT-A1,^28^ generated from a unique peptide library with a quasi-reversible bicyclic topology. We have shown that the binding was enabled by the rigid backbone, validating the benefits of implementing bicyclic structures to target proteins.^29−33^ We also proved that the binding was sensitive to the spatial arrangements of the functional groups. While this finding pointed to a discrete hit distribution landscape in the 3D chemical space and obscured the pathway for optimization, it also hinted at the possibility of identifying more MYC binders through exploring additional 3D chemical space.

To this end, we sought to explore strategies to access the 3D diversifiable chemical space beyond that sampled by our previous NTA constructs. An obvious path would be to retain the NTA scaffold and increase the number of modular amino acids. Here, adding an additional amino acid module will cause an 18-fold expansion of the chemical diversity. However, this expansion has several potential pitfalls. First, it will increase the physical size of the library by the same magnitude, quickly rendering the screening process unpractical. Second, this approach preserves the backbone structure and the corresponding privileged conformational space. Consequently, it will likely access similar 3D chemical space as that provided by the original 5-mer scaffold. Third, expanding the ring size may confer additional flexibility to the backbone and, therefore, may elicit an entropic penalty and impede the binding. Taken together, these considerations urged us to design different backbone scaffolds to potentiate the search for MYC binders.

**Figure 1:**
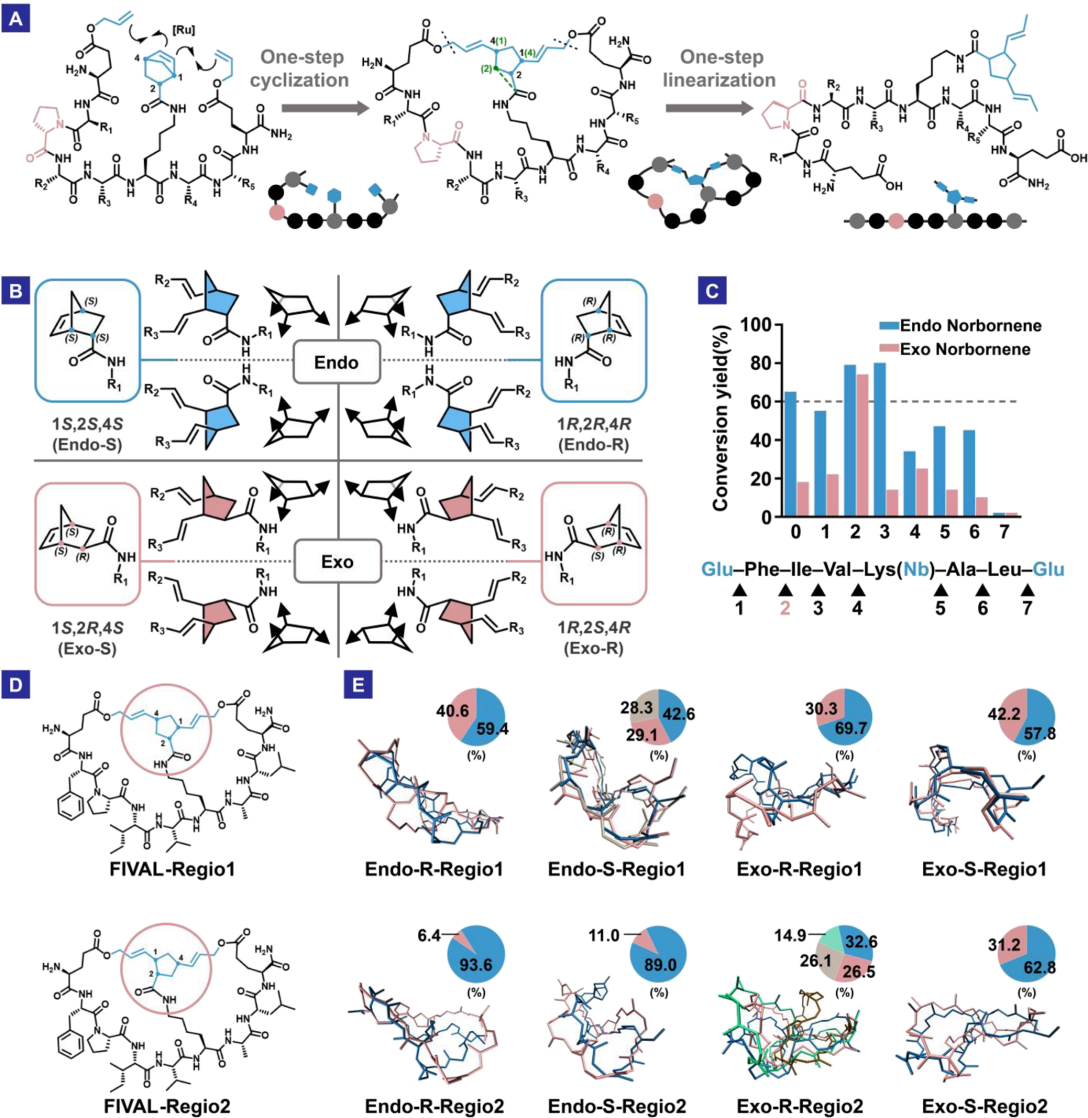
(A) Macrocyclization and linearization of the bicyclic peptide through ROM-RCM and deallylation reactions. In the cartoon illustration, the balls represent amino acid residues, and other solid shapes depict the functional groups that participate in the reaction. Green numbers and the green dashed bond illustrate regioisomerism. (B) Stereodiversity of 5-norbornene-2-carboxylic acid amide derivatives before and after cyclization. The eight isomers exhibit distinct stereo structures when combined with all l-amino acids. (C) Proline scanning results. The numbers indicate the positions of proline insertion in the linear precursor. The reaction yield was semiquantitatively determined by mass spectrometry. (D) Structures of the model peptide (FIVAL) regioisomers. (E) Conformation clusters of each isomer illustrated by different colors. Endo and exo refer to the stereostructure of the norbornene building block. The percent population of each conformation cluster is shown in the pie charts with corresponding colors.

Herein, we present a stereodiversified bicyclic peptide library. Using norbornene and allyl ester building blocks, we demonstrate the first example of a tandem ring-opening metathesis and ring-closing metathesis (ROM-RCM) strategy to construct a bicyclic scaffold (Figure 1A). From this library, we identified a new set of MYC-binding bicyclic peptides with submicromolar affinity and validated their bioactivities in human cancer cell lines.

## Results and Discussion

### Design Principles of the Bicyclic Peptide Scaffold

Our objectives here are (1) accessing a 3D chemical space different from that of our NTA library, (2) increasing library diversity, and, more importantly, (3) avoiding library physical size inflation. Inspired by the early works where stereodiversity enabled chemical space expansion, we sought to construct a library using building blocks with side-chain stereocenters. Within the premise of solid-phase libraries, we envisioned that racemic building blocks could provide stereodiversity on the same resin, eliminating the need to expand the physical size of the library. With careful construction, such a one-bead-multiple-compounds design would not interfere with the library screening process. After the screening, the identified hit sequences can be synthesized using optically pure building blocks, and the products can be validated for their binding ability. We reasoned that a single building block with multiple stereocenters would be the most efficient and suitable approach for practical feasibility.

Based on those objectives, we introduced a norbornene group and attached it to a lysine side chain. We proposed a tandem ROM-RCM reaction to construct the bicyclic topology, where the norbornene group could transform into a cyclopentane structure with three stereocenters. Considering regioisomerism, this process can generate eight isomers (Figure 1B). Similar to the NTA library, this bicyclic topology can be transformed to a linear form using a Pd-catalyzed deallylation reaction, enabling *de novo* hit sequencing by tandem mass spectrometry.

Nevertheless, the design above is synthetically challenging for three reasons: (1) there has been no successful implementation of ROM-RCM reactions for constructing large cyclic structures, and its feasibility remained questionable, (2) the correct reaction required the simultaneous formation of two cyclic structures, competing with other equally possible constructs, and (3) the success of the bicyclic peptides’ construction and linearization could be sequence-dependent.

### Construction of the Bicyclic Peptide Library

Built upon our experience with the NTA library, we hypothesized that a proline residue could facilitate the formation of the bicyclic structure by promoting privileged conformations. Therefore, we did a “proline scanning” on a model peptide sequence (FIVAL), using racemic *endo*- and *exo*-norbornene building blocks. We performed the metathesis reaction using the Hoveyda–Grubbs second generation catalyst (M720) and compared product yields using mass spectrometry. As expected, the location of the proline residue had profound influences on the yield, and *endo*- and *exo*-constructs exhibited different activities (Figure 1C). Interestingly, only position 2 proline insertion exhibited high yields for both isomers, and this backbone structure was chosen for our subsequent studies. We also evaluated a similar proline scanning on a more complex model sequence (WQYRH) and confirmed that position 2 was the best (Figure S1). Three more random sequences were also tested with proline in position 2, and they all showed a reasonable conversion yield (Figure S2).

As discussed above, this reaction contains multiple routes that can lead to different metathesis products. To validate the proposed route, we performed two tests (Figures S3–S5). First, using a tetrazine test, we confirmed that the norbornene residue was transformed during the metathesis reaction. Second, using Edman degradation and mass spectrometry, we confirmed that both allyl groups were transformed during the metathesis. Taken together, these results proved the successful construction of the desired bicyclic structure depicted in Figure 1A.

In order to gain more insight into how the *endo*-*exo* isomerism confers structural diversity, we performed atomistic molecular dynamics simulations with explicit water molecules on the FIVAL model peptide. Here, we saw that all eight isomers divided into two groups based on regioselectivity (Figure 1D) adopted stable conformations within the 1 μs simulation length. More interestingly, each isomer exhibited multiple conformation clusters featuring distinct ring pucker directions (Figure 1E). Further analyses also revealed differential levels of backbone fluctuation (Figure S6). These results underscore the prominent spatial diversity sampled by the bicyclic structure, which supports our design.

Using the conditions identified above, we prepared a bicyclic peptide library through the split-pool method (Figures 2 and S7). We employed the racemic *endo*- and *exo*-norbornene isomers to generate the bicyclic backbone and used 17 canonical amino acids as variable building blocks. We installed an alkyne residue at the N-terminal of the sequences and denoted the library as NTB. To validate the library quality, we randomly picked 42 beads and performed linearization and sequencing. We confirmed that 40 of the beads were sequenceable, supporting the quality of the NTB library (Figures S8–S47).

**Figure 2:**
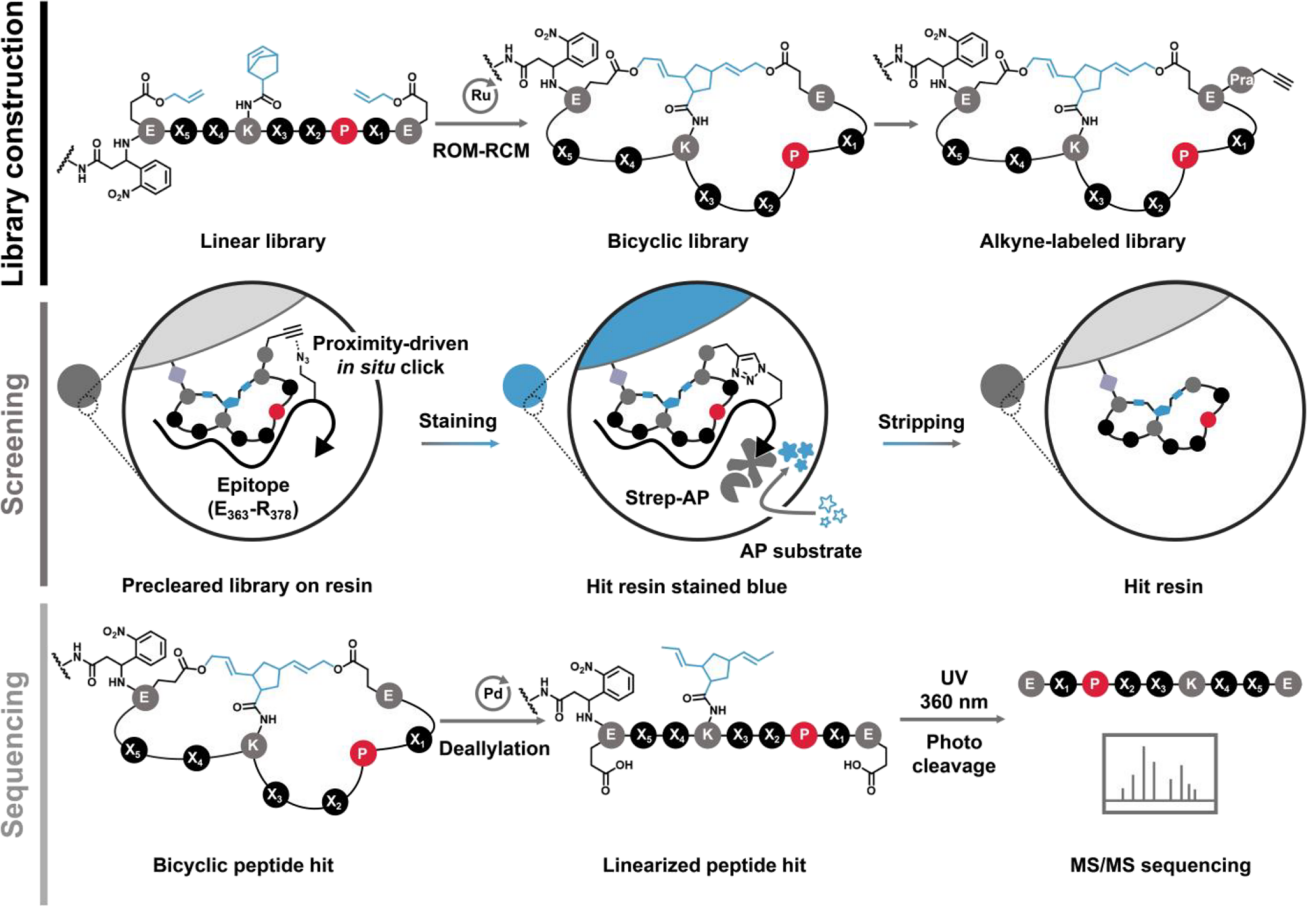
NTB bicyclic peptide library construction, MYC epitope screening, and chemical linearization of hit peptides followed by MS/MS sequencing.

### Generation of MYC-Binding Hits from the NTB Library

We proceeded to perform library screening against the MYC E_363_-R_378_ epitope, following our established protocols (Figures 2 and S7). This epitope is involved in the MYC-MAX-DNA interaction and was used in our NTA study. Here, the epitope was chemically synthesized and modified with a biotin group and an azido-lysine residue (Figures S48 and S49). Precleared NTB library was incubated with the epitope, and a proximity-driven *in situ* click reaction labeled the potential hits with a biotin group, which allowed for hit identification through enzyme-amplified colorimetric reactions.

The screening generated eight hits (Figure S50), corresponding to 64 structures. Using racemic *endo*- and *exo*-norbornene isomer mixtures, we synthesized these hits at milligram-scale quantities (Figures S51–S58) and evaluated their binding to recombinant MYC (Figure S59) by ELISA. As shown in Figure S60, many of the hits exhibited micromolar-level binding affinities to MYC, with NT-B2 reaching a high-nanomolar level (Figure 3A,B). These results were consistent with competitive ELISA results using unlabeled hit sequences (Figures S61–S69). Using protein thermal shift assays, we further validated that NT-B2 was able to bind to recombinant MYC and affect its thermal stability (Figures 3C and S60).

**Figure 3:**
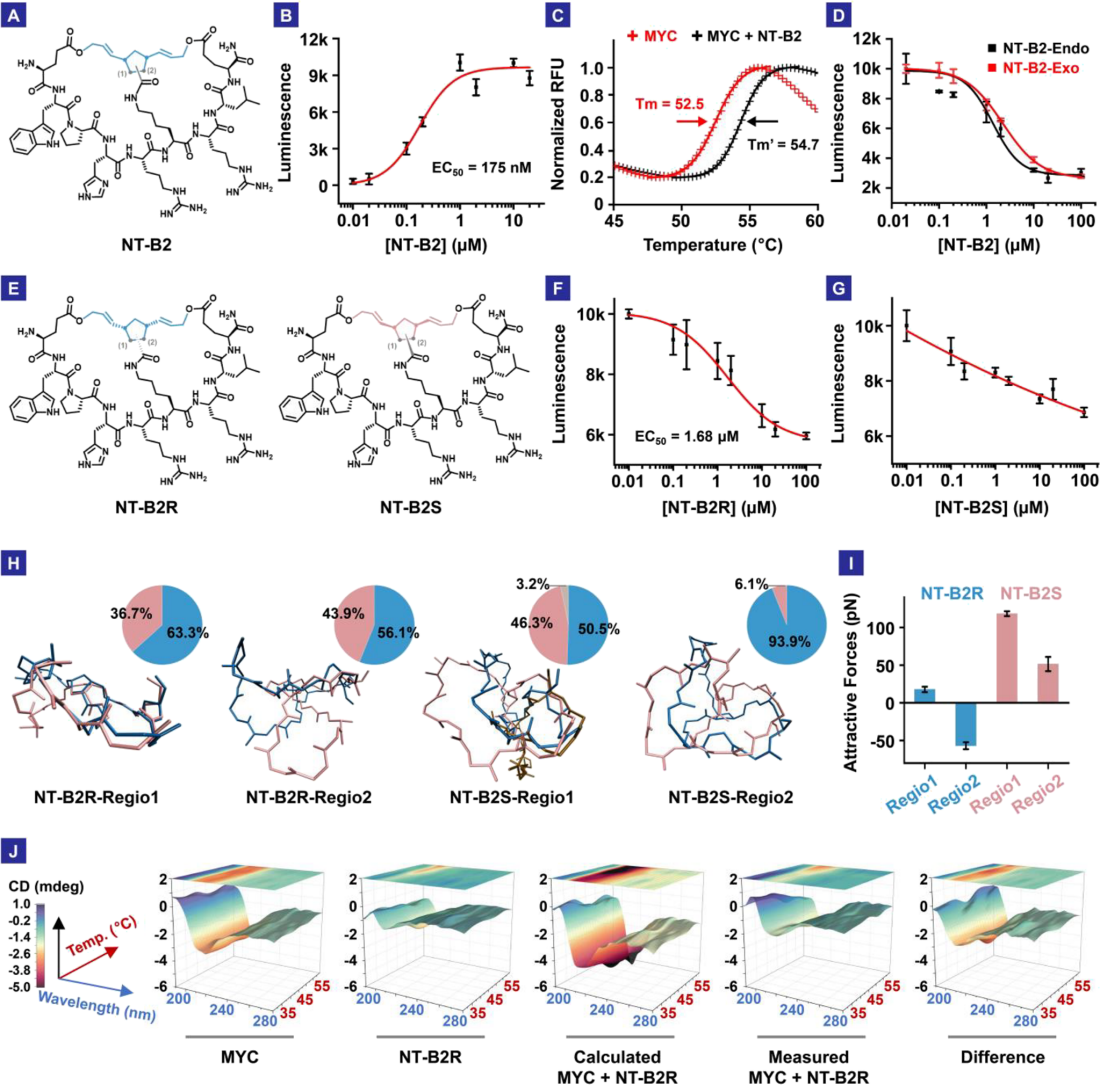
Hit peptide NT-B2 binding analysis and molecular dynamics calculation analysis. (A) The structure of NT-B2 (racemic *endo*- and racemic *exo*-isomers). The gray bond could connect to C-1 or C-2, forming two regioisomers. (B) ELISA results of biotin-PEG5-NT-B2 vs His-tagged MYC protein. (C) Protein thermal shift assay results. Binding with NT-B2 increased MYC melting temperature by 2°. (D) Competitive ELISA results of NT-B2-Endo and NT-B2-Exo vs biotin-PEG5-NT-B2. (E) Structures of NT-B2R and NT-B2S. The gray bond could connect to C-1 or C-2, forming two regioisomers. (F) Competitive ELISA results of NT-B2R (*endo*) vs biotin-PEG5-NT-B2. (G) Competitive ELISA results of NT-B2S (*endo*) vs biotin-PEG5-NT-B2. (H) Molecular dynamics calculation results of the four NT-B2 isomers. The percent population of each conformation cluster is shown in the pie charts with corresponding colors. (I) Attractive force analysis of NT-B2R and NT-B2S. (J) Circular dichroism (CD) spectra of MYC only, NT-B2R only, the calculated and measured mixture of MYC and NT-B2R, and the difference between the calculated and measured mixture spectra.

We synthesized NT-B2-Endo (racemic *endo*-isomers) and NT-B2-Exo (racemic *exo*-isomers) and evaluated their MYC-binding abilities using competitive ELISA (Figures 3D and S70 and S71). The two groups of peptides showed similar binding affinities, while NT-B2-Endo performed slightly better. Considering that the synthesis of the enantiopure *endo*-norbornene precursor was less challenging, we proceeded with preparing NT-B2-Endo isomers (NT-B2R and NT-B2S, Figures S72–S75) shown in Figure 3E.

Interestingly, the NT-B2R synthesis produced two isomers with distinct retention times (Figure S73A). Based on MALDI signatures, we reasoned that the two peaks corresponded to NT-B2R regioisomers. Through tandem HPLC purifications, we were able to obtain the dominant isomer (Figure S73B), although its exact regioidentity was elusive. On the contrary, the two NT-B2S isomers were not separatable under our HPLC conditions (Figure S75). Hereafter, we performed subsequent studies using this seemingly pure NT-B2R isomer and the mixture of NT-B2S isomers.

The two groups of isomers, *R* vs *S*, exhibited drastically different binding abilities to MYC (Figures 3F,G and S76). To understand this difference, we again performed molecular dynamics simulations and postanalysis. The results showed that the NT-B2R group was considerably more stable than the NT-B2S group, evidenced by its fewer conformation clusters and more prominent intra-ring attractive forces (Figure 3H,I). Interestingly, the levels of backbone RMSD fluctuation were not significantly different (Figure S77).

To further study the nature of the interaction between NT-B2R and MYC, we leveraged temperature-dependent circular dichroism (CD) spectroscopy. Here, if no conformation change was involved, the CD spectra of the MYC/NT-B2R mixture would be the addition of each component’s spectra (Figure 3J, calculated). However, we observed drastically different CD spectra (Figure 3J, measured and difference). The results here validated the direct interaction between NT-B2R and MYC and indicated that the interaction led to significant changes in the conformation landscape.

### NT-B2R Binds to MYC and Inhibits MYC Activities In Vitro

**Figure 4:**
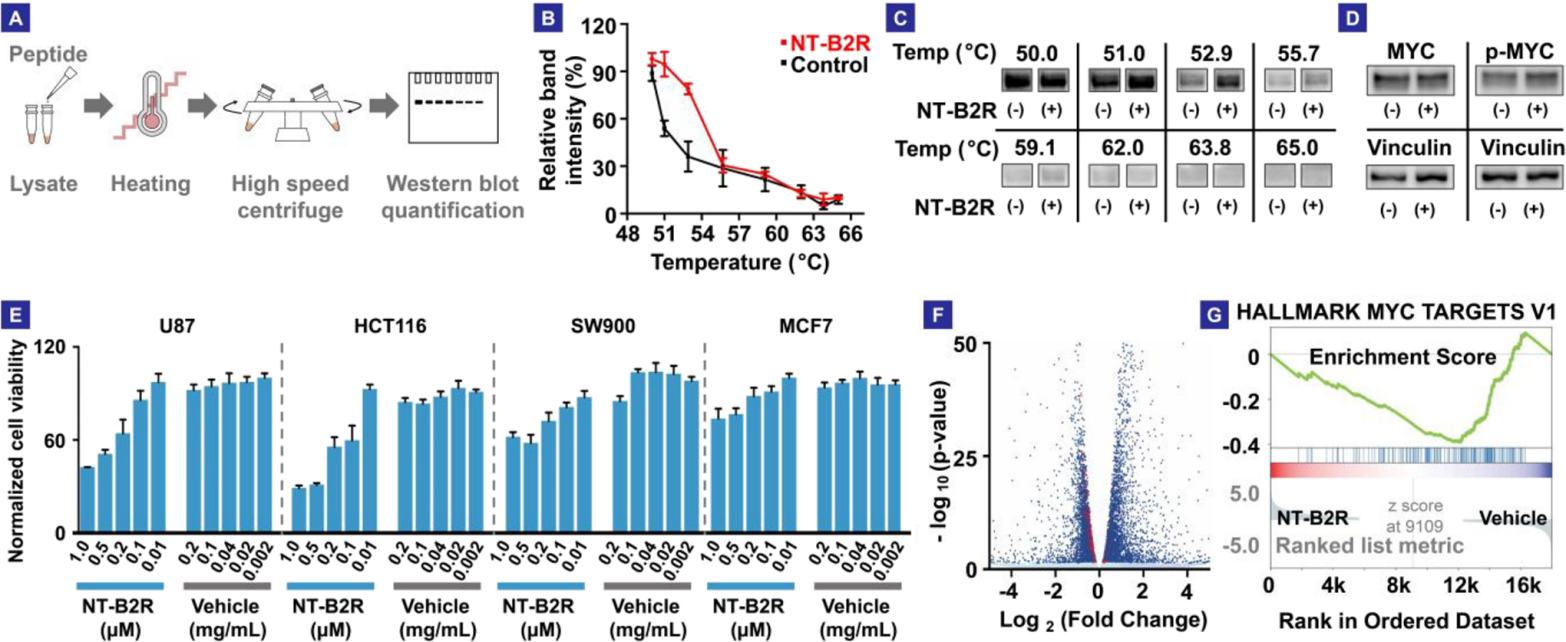
(A) Workflow of the cellular thermal shift assay (CETSA). (B) CETSA results of NT-B2R versus control with U87 cell lysate. The MYC melting temperature increased by ∼2°, proving the binding between NT-B2R and MYC. (C) Western blot results of CETSA. (D) Western blot results showing unchanged MYC and phosphor-MYC (p-MYC, T58) levels in U87 cells treated with NT-B2R. (E) Viability results of U87, SW900, HCT116, and MCF7 cells. NT-B2R-loaded liposomes were used to treat the cells. Corresponding concentrations of empty liposomes were used as vehicle controls. (F) Volcano plot showing differentially expressed genes in NT-B2R-treated U87 cells, compared to vehicle-treated cells. Genes with adjusted *p*-values >0.05 are colored light blue. Genes with adjusted p-values <0.05 are colored dark blue. Genes from the HALLAMRK MYC TARGETS V1 gene set are colored red. (G) Gene set enrichment analysis (GESA) result showing that the HALLAMRK MYC TARGETS V1 gene signatures were significantly enriched in vehicle-treated cells. NES, normalized enrichment score: −5.955; *p-*value: < 0.001; FDR, false discovery rate: 0.005.

To validate that NT-B2R could bind to MYC in complex biological environments, we performed cellular thermal shift assays using U87 cell lysates^34^ (Figure 4A). U87 is a human glioblastoma cell line with known dependence on MYC activities.^35−37^ As expected (Figures 4B,C and S78), NT-B2R significantly affected the thermal properties of MYC, supporting direct interactions between them in a more complex binding environment.

We then evaluated the biological effects of NT-B2R in cells. Because NT-B2R was not cell-permeable, we leveraged liposomes as delivery vehicles (Figure S79). We first confirmed that the NT-B2R treatment did not alter the expression and phosphorylation levels of MYC (Figure 4D), which was consistent with the expected mechanism of action. We observed that NT-B2R treatment led to decreased metabolic activity and proliferation in U87 cells (Figure 4E). We also obtained similar results using HCT116, SW900, and MCF7 cell lines (Figures 4E and S80).

To test whether NT-B2R directly affected MYC activity, we performed RNA-seq on the U87 cells and evaluated the differentially expressed genes using DESeq. As shown in Figure 4F, NT-B2R treatment caused significant changes in the transcriptome, with 704 genes significantly downregulated and 1322 genes upregulated, consistent with MYC’s master transcription factor role. A caveat here is that a global shift of transcription activities will not be observable by this analysis, due to the embedded normalization procedure.^38^ Nevertheless, gene set enrichment analysis (GSEA) results highlighted that MYC target gene sets were enriched with high statistical significance and low false discovery probabilities, supporting that MYC was NT-B2R’s main target (Figure 4G).

## Conclusions

In summary, we constructed a stereodiversified bicyclic peptide library (NTB) using a norbornene residue and tandem ROM-RCM reactions. This library highlighted the absence of triazole rings and more hydrophobic backbones, providing access to a new chemical space. Admittedly, identifying and obtaining optically pure hits can be challenging, which may confound downstream medicinal chemistry campaigns. Nevertheless, this limitation does not detriment the value of the NTB library for three reasons. First, stereomixtures can become useful pharmaceutics if the undesired isomers do not induce adverse effects. Second, the stereomixtures provide the starting point for tackling undruggable targets. Third, with the advancement of chiral separation methods and stereoselective synthetic strategies, elucidating the exact stereoidentity can become feasible.

Using the NTB library, we identified a MYC-targeting bicyclic peptide, NT-B2R, which could directly inhibit MYC transcription activities without affecting MYC expression or phosphorylation levels. We envision that further iterations of NT-B2R, as well as the orthogonal chemical space accessible to additional multicyclic peptide libraries, could lead to more potent MYC inhibitors for biomedical studies.

## Supporting Information Available

Procedures for bicyclic peptide synthesis and chemical linearization, examples of randomly picked sequences from the bicyclic peptide library, cell culture, and other experimental details (PDF)
PDB files of the MD calculation results (ZIP)
