# Supplementary material for: MYC-Targeting Inhibitors Generated from a Stereodiversified Bicyclic Peptide Library
Source: J Am Chem Soc. 2024 Jan 3;146(2):1356–63. doi: 10.1021/jacs.3c09615 (PMC10797614; doi:10.1021/jacs.3c09615)
Supplement: Supplementary file 1 — ja3c09615_si_001.pdf [file ja3c09615_si_001.pdf]

MYC-targeting inhibitors generated from a stereodiversified bicyclic peptide library

Zhonghan Li[†,*], Yi Huang[†], Ta I Hung[†], Jianan Sun[‡], Desiree Aispuro[‡], Boxi Chen[†], Nathan Guevara[†], Fei Ji[†], Xu Cong[†], Lingchao Zhu[†], Siwen Wang[‡], Zhili Guo[†], Chia-en Chang[†,‡,*] and Min Xue[†,‡,*]

† Department of Chemistry, University of California, Riverside, Riverside, California 92521, United States.

‡ Environmental Toxicology Graduate Program, University of California, Riverside, Riverside, California 92521, United States

Supporting Information

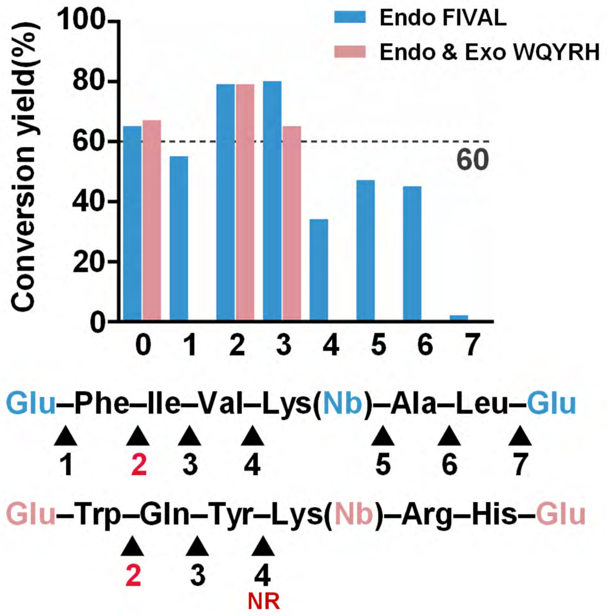

Figure S1. Proline scan for a hindered sequence WQYRH prepared with endo- and exo-mixed norbornene. For the WQYRH sequence. Positions 0, 2, 3, 4 were selected based on the FIVAL sequence results. Position 2 showed excellent conversion yield here, while position 4 showed no reaction, which is consistent with the observed low yield at this position in the FIVAL sequences.

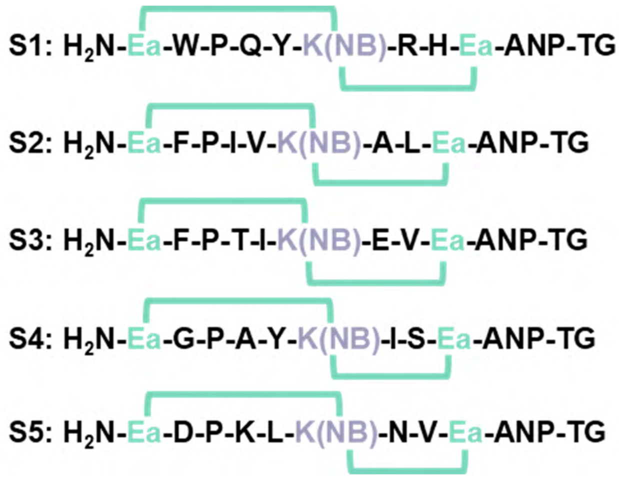

Conversion yield of the ROM-RCM reaction (%)	
S1:	85
S2:	83
S3:	90
S4:	88
S5:	80

Figure S2. Model sequences for ROM-RCM reaction evaluation with proline inserted at position 2. NB represents endo-/exo-mixed norbornene. ANP represents the 3-Amino-3-(2-nitro-phenyl)propionic acid linker. TG represents TantaGel resin. All sequences showed conversion yield above 80% (semi quantification by mass spectrometry).

Experiment 1: On resin reaction with tetrazine acid (red) and photocleavage for mass spectrometry

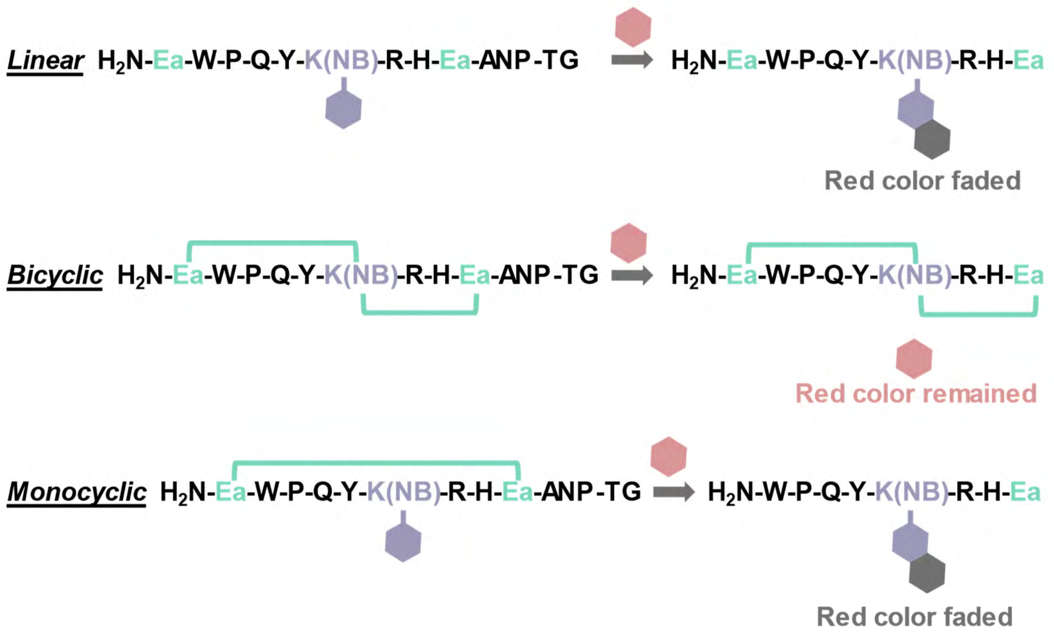

Experiment 1 results

	Color	Mass with tetrazine adduct
Linear-S1	Faded	Found. (Dominant)
Linear-S2	Faded	Found. (Dominant)
Linear-S3	Faded	Found. (Dominant)
Linear-S4	Faded	Found. (Dominant)
Linear-S5	Faded	Found. (Dominant)
Cyclic-S1	Remained	Found. (Trace amount)
Cyclic-S2	Remained	Not found.
Cyclic-S3	Remained	Found. (Trace amount)
Cyclic-S4	Remained	Found. (Trace amount)
Cyclic-S5	Remained	Not found.

Figure S3. To confirm successful bi-cyclization reactions, a fast-click reaction between tetrazine acid (red) and norbornene was used. In this reaction, the red color of the tetrazine acid solution turns colorless upon reacting with norbornene. If norbornene is involved in the ROM-RCM cyclization, the tetrazine acid will remain intact and the red color will remain. Conversely, if the cyclization is RCM-only (norbornene is not involved in the cyclization), the red color will fade. All starting materials with intact norbornene led to faded color, albeit with different kinetics. The cyclization products led to a persistent red color, indicating successful ROM-RCM reactions. These results were also confirmed by mass spectrometry.

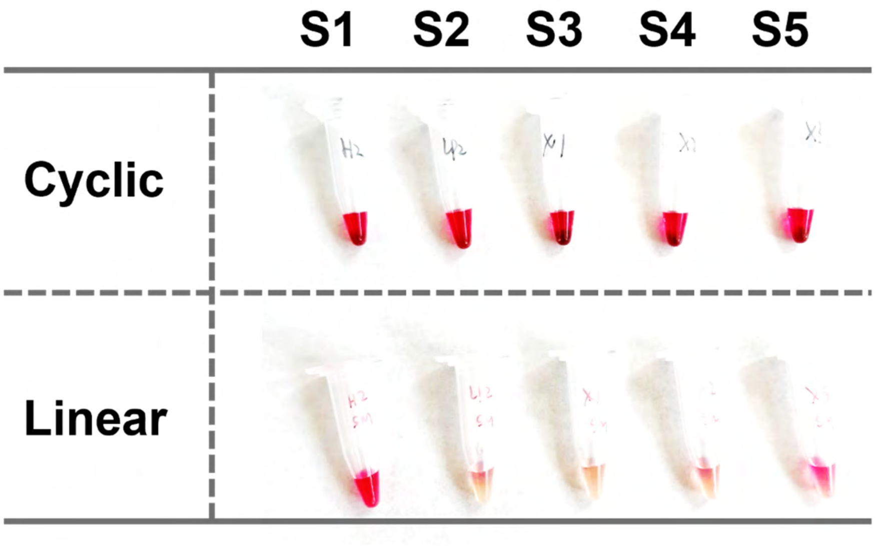

Figure S4. Tetrazine assay results. The red color persisted for all cyclization products, supporting the ROM-RCM reaction mechanism and proving the norbornene participation.

Experiment 2: Edman degradation followed by deallylation and photo cleavage

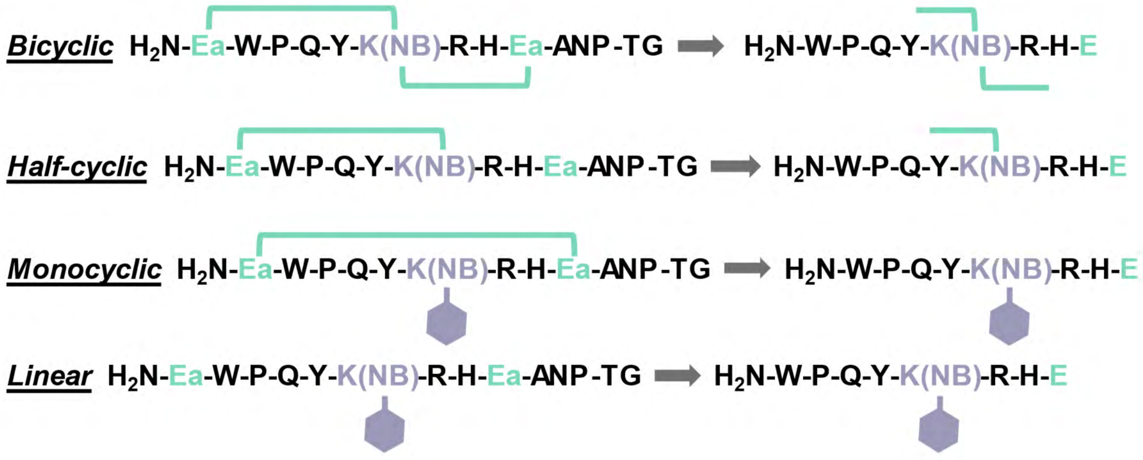

Experiment 2 results

	Bicycle mass	Half-cycle mass	Mono(linear) mass	MSMS confirmed
S1	Main peak	Not found	Not Found	Yes ✓
S2	Main peak	Trace	Not Found	Yes ✓
S3	Main peak	Not found	Not Found	Yes ✓
S4	Main peak	Not found	Not Found	Yes ✓
S5	Main peak	Not found	Not Found	Yes ✓

Figure S5. Successful bicyclic structure formation can be confirmed by various methods. After undergoing Edman degradation and de-allylation, bicyclic products will display distinct MS and MS/MS patterns with di-propenyl norbornene adducts (MW: 304 or 302). In contrast, an intermediate product (head-to-middle half-cyclization, norbornene-involved cyclization) would reveal a fragment with a propenyl norbornene adduct (MW: 288), while an unsuccessful cyclization (starting material, norbornene not involved) or mono-cyclization (head-to-tail cyclization, norbornene not involved) would demonstrate a norbornene fragment (MW: 248). For ROM-RCM (bi-cyclization and half-cyclization) and RCM (mono-cyclization) reactions, the molecular weight (MW) will decrease by 28 (ethylene released).

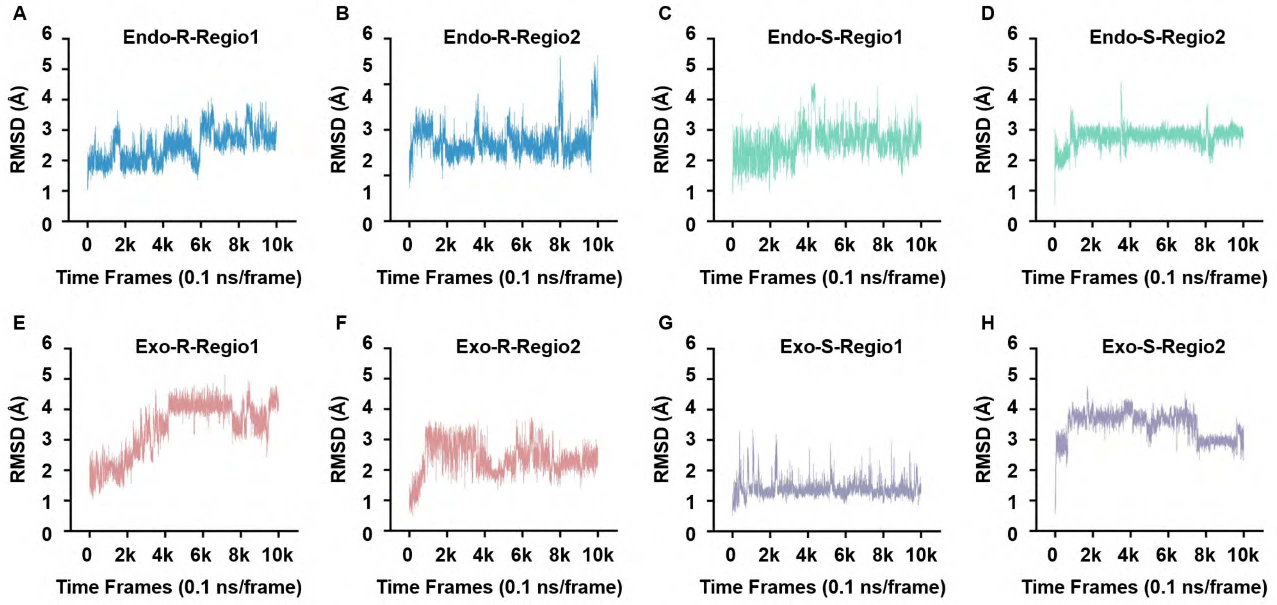

Figure S6. Backbone RSMD calculation for FIVAL isomers.

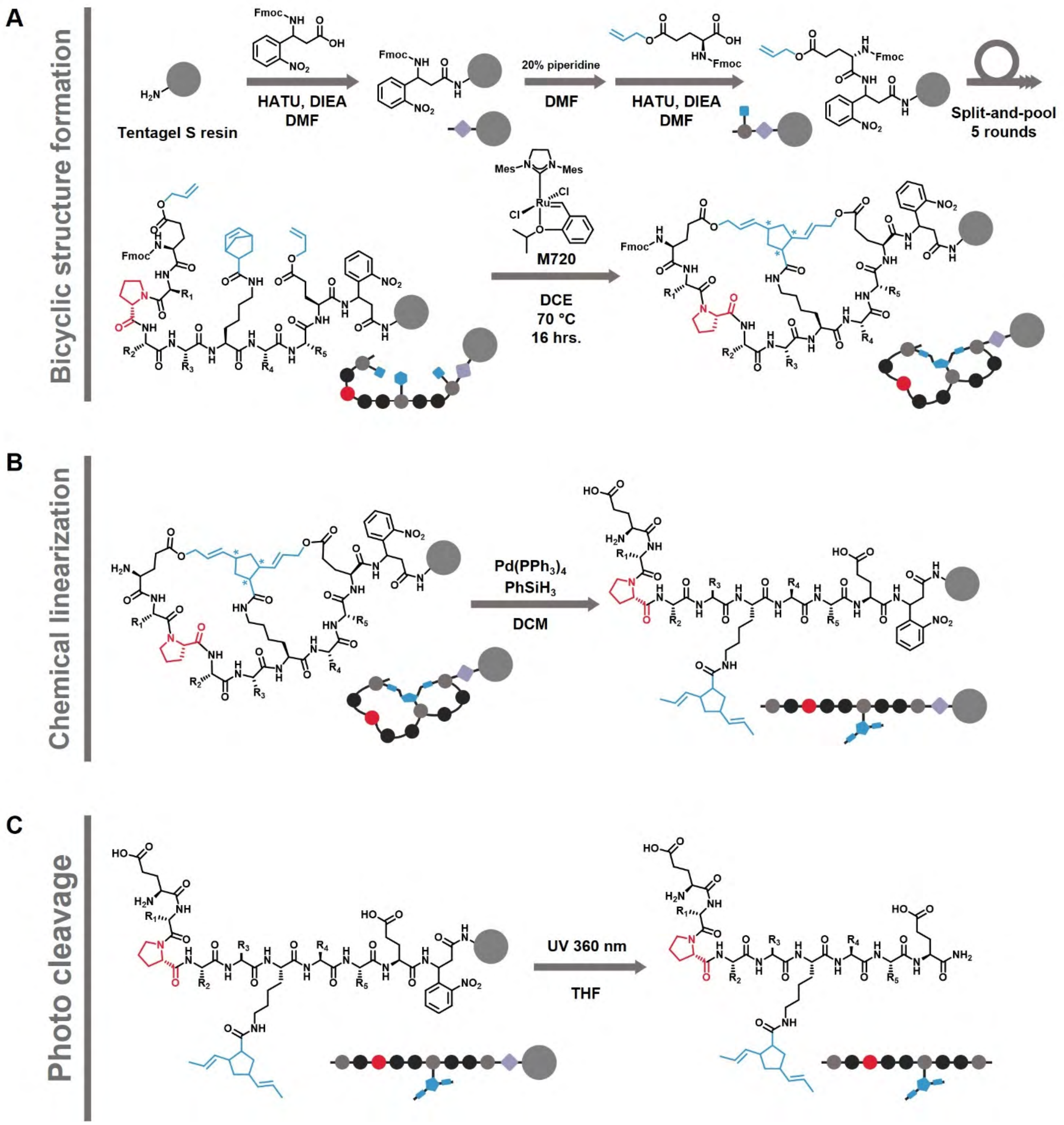

Figure S7. Synthesis of the NTB bicyclic peptide library. (A) Bicyclic structure formation through a one-step ROM-RCM reaction. (B) Chemical linearization of the bicyclic peptide for MSMS sequencing with one-step de-allylation. (C) Photo cleavage.

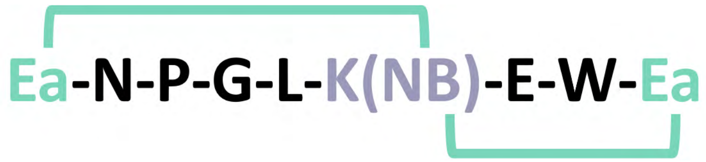

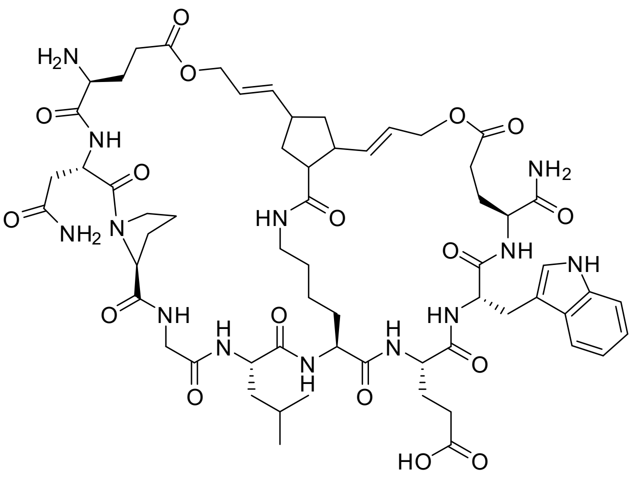

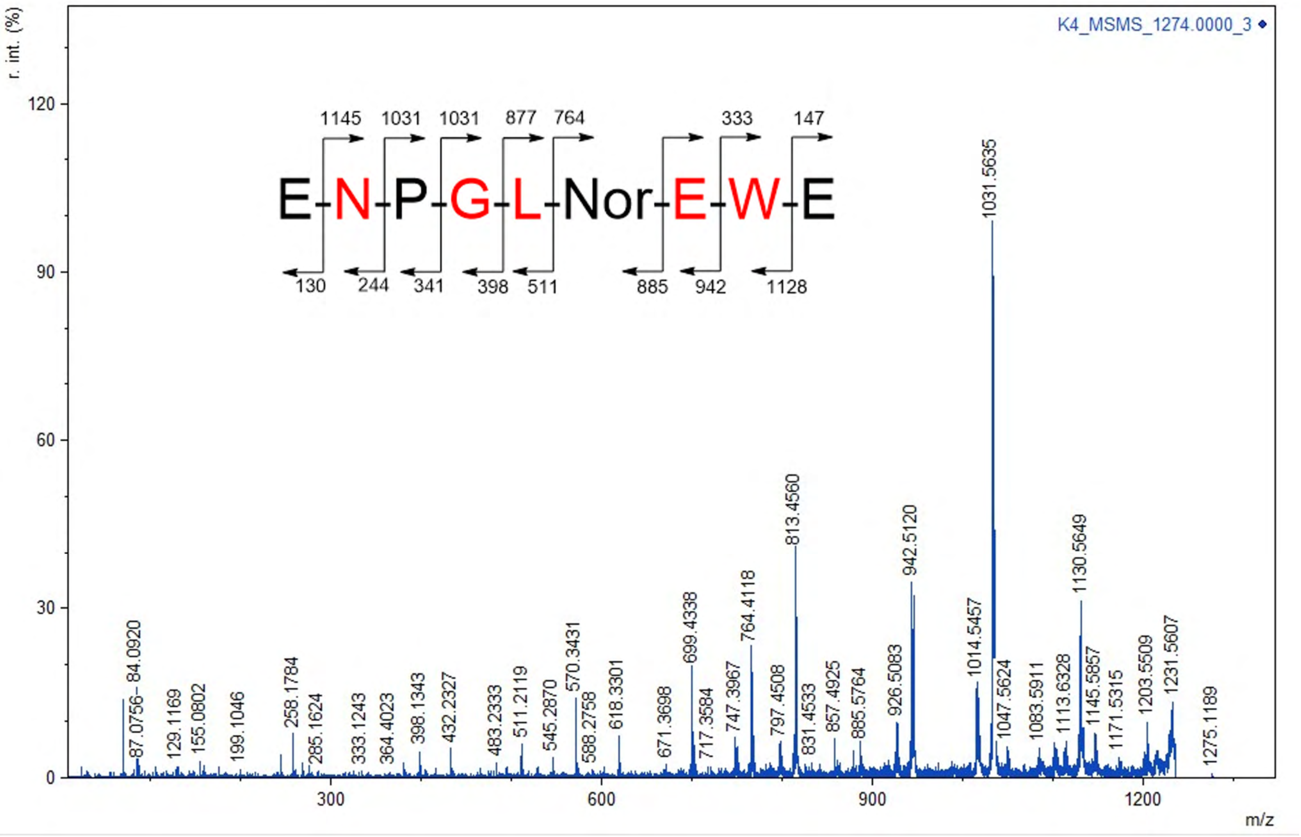

Figure S8. MS/MS of a random bead from the library, N-G-L-E-W. Parent ion: 1274.00.

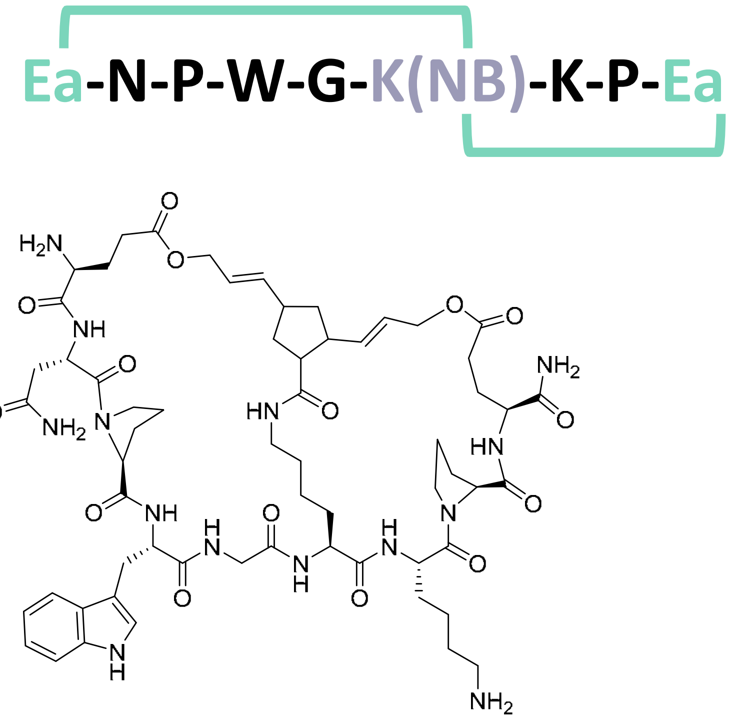

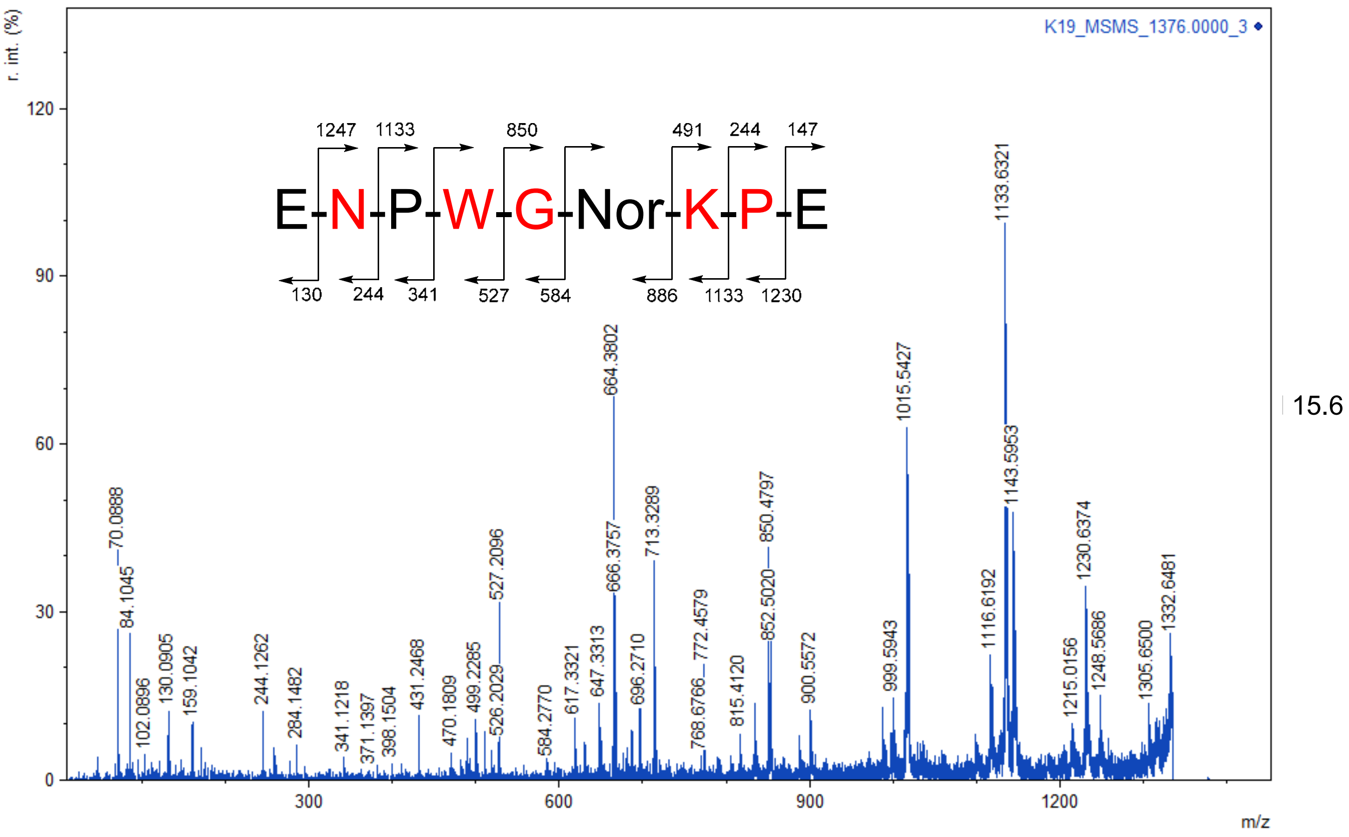

Figure S9. MS/MS of a random bead from the library, N-W-G-K-P. Parent ion: 1376.00.

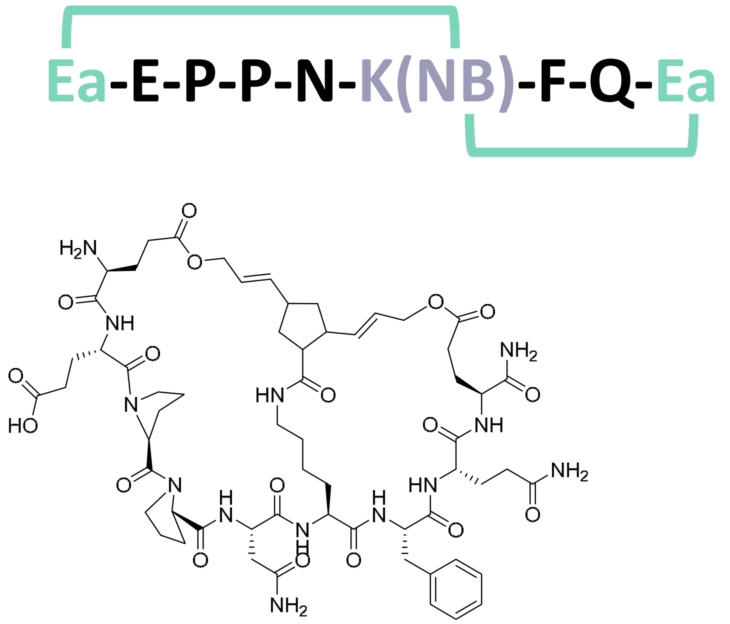

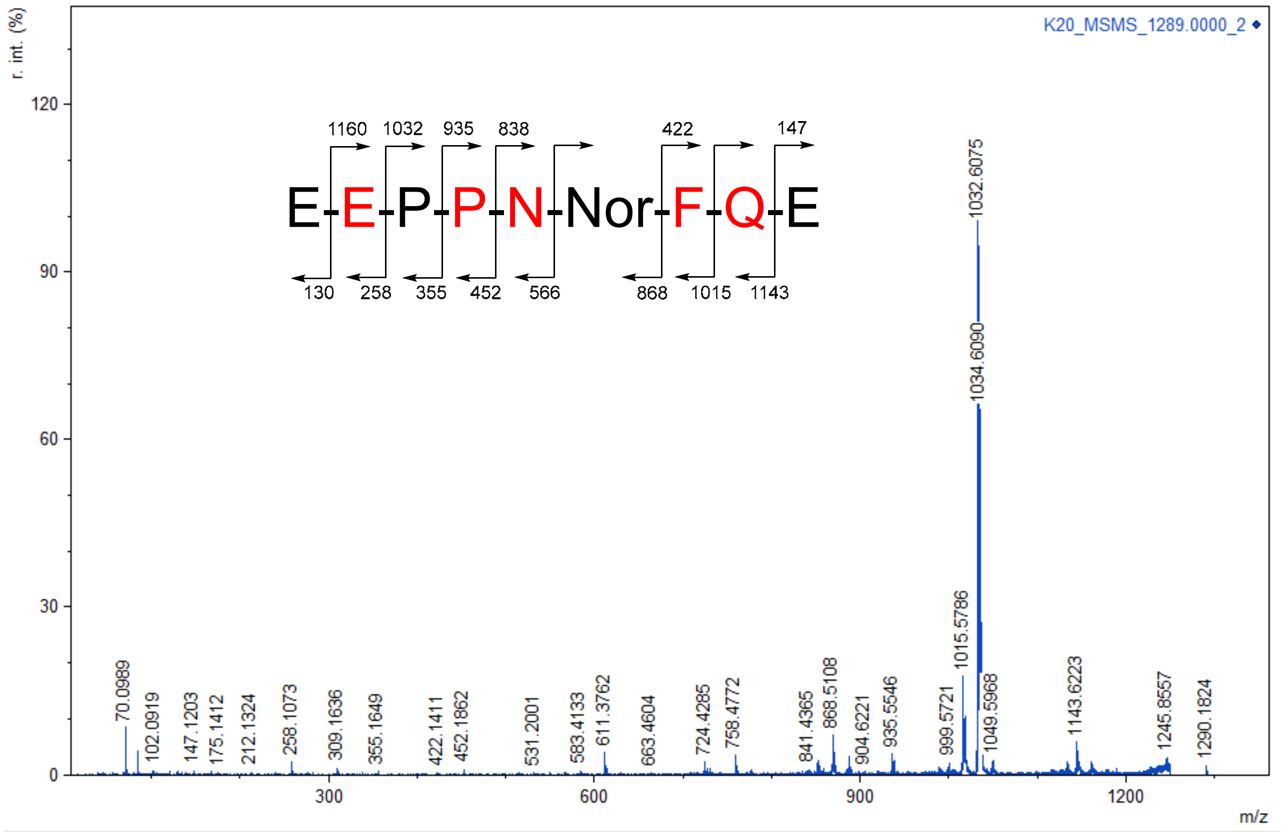

Figure S10. MS/MS of a random bead from the library, E-P-N-F-Q. Parent ion: 1289.00.

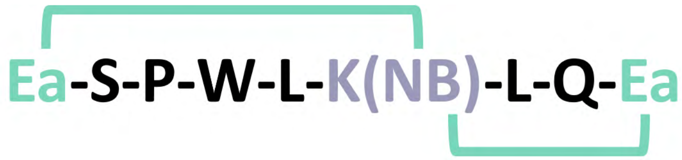

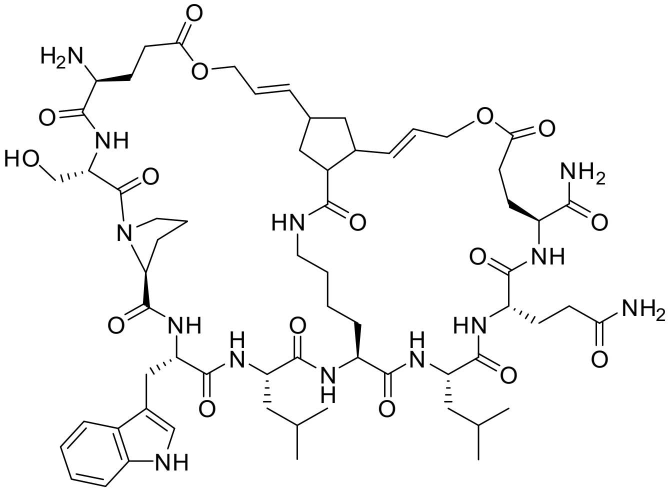

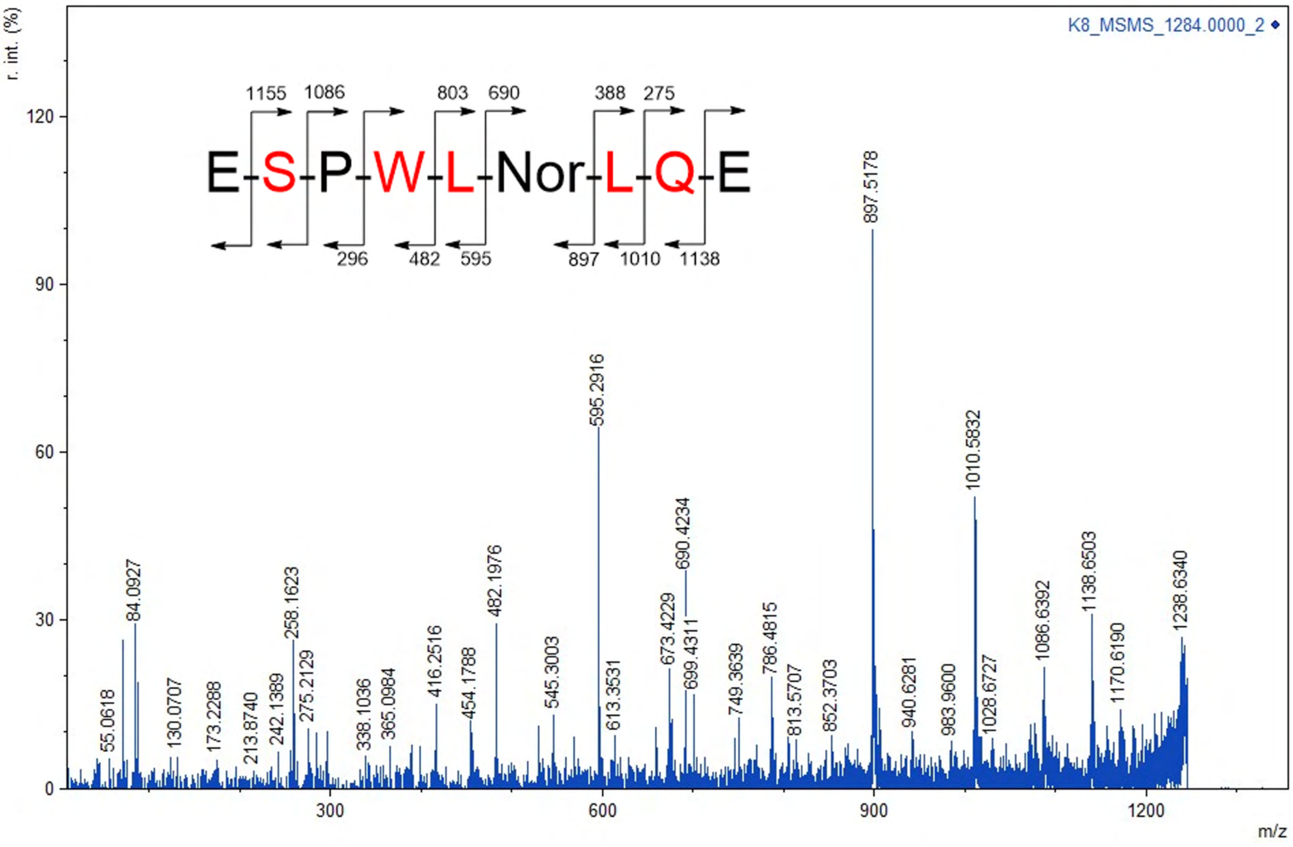

Figure S11. MS/MS of a random bead from the library, S-W-L-L-Q. Parent ion: 1284.00.

Ea-E-P-V-D-K(NB)-P-H-Ea

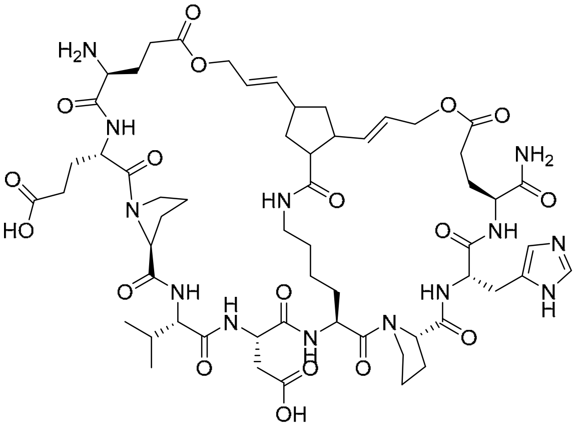

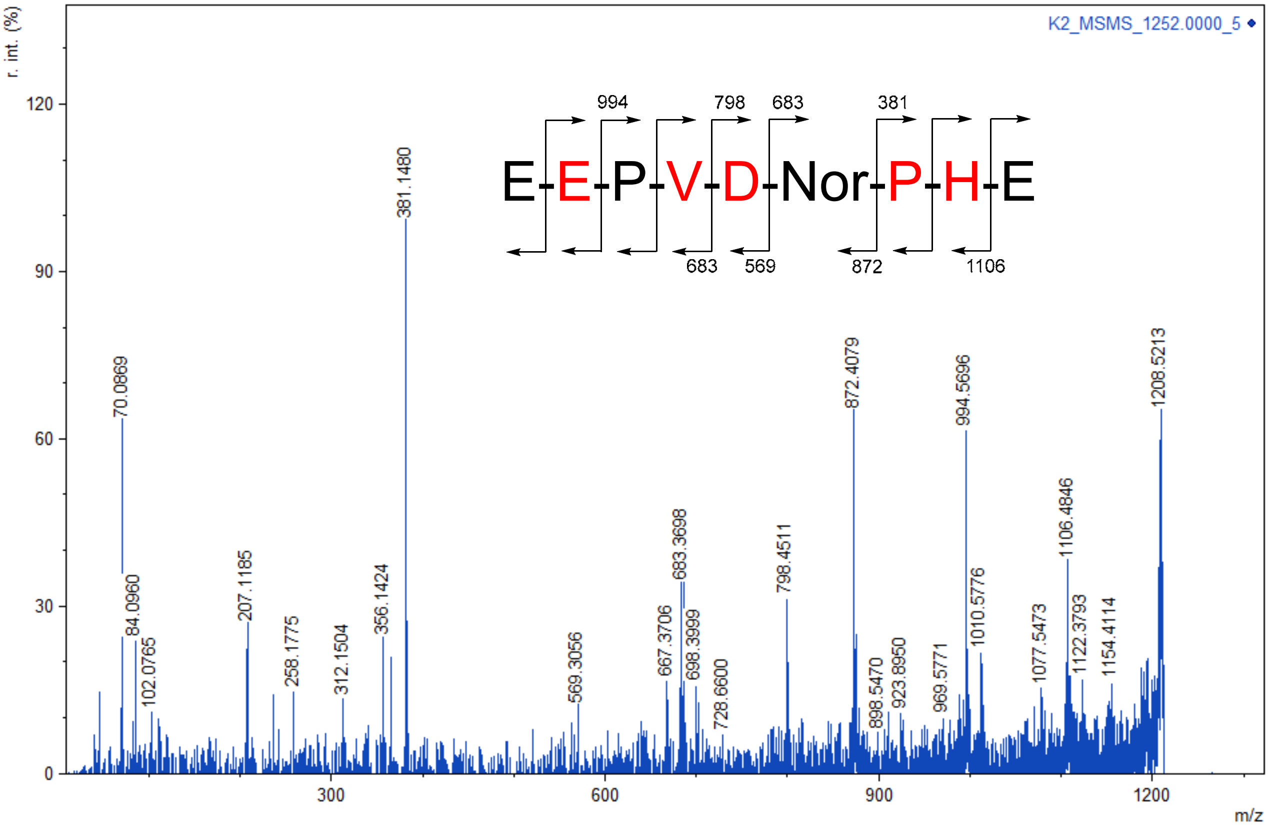

Figure S12. MS/MS of a random bead from the library, E-V-D-P-H. Parent ion: 1252.00.

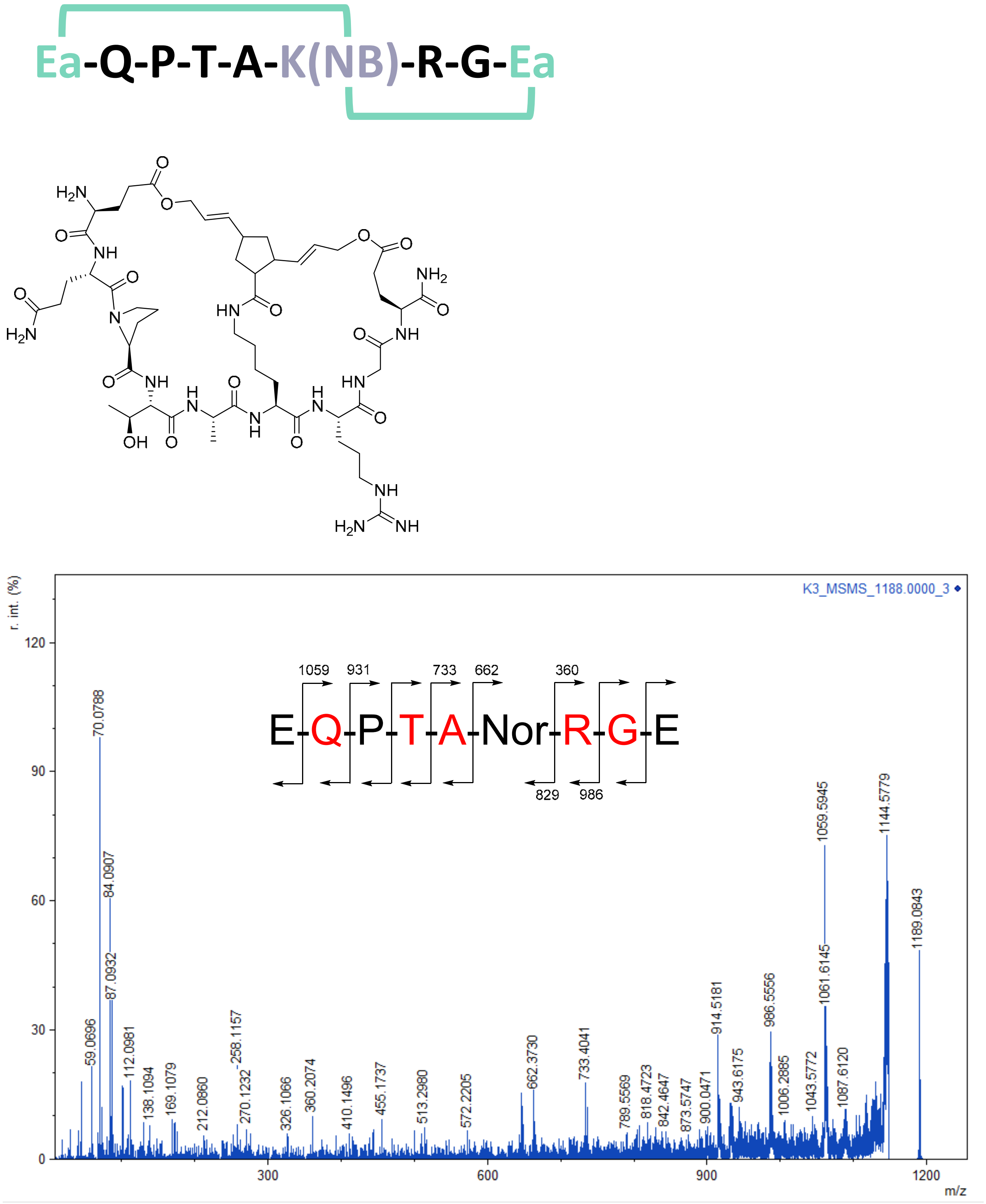

Figure S13. MS/MS of a random bead from the library, Q-T-A-R-G. Parent ion: 1188.00.

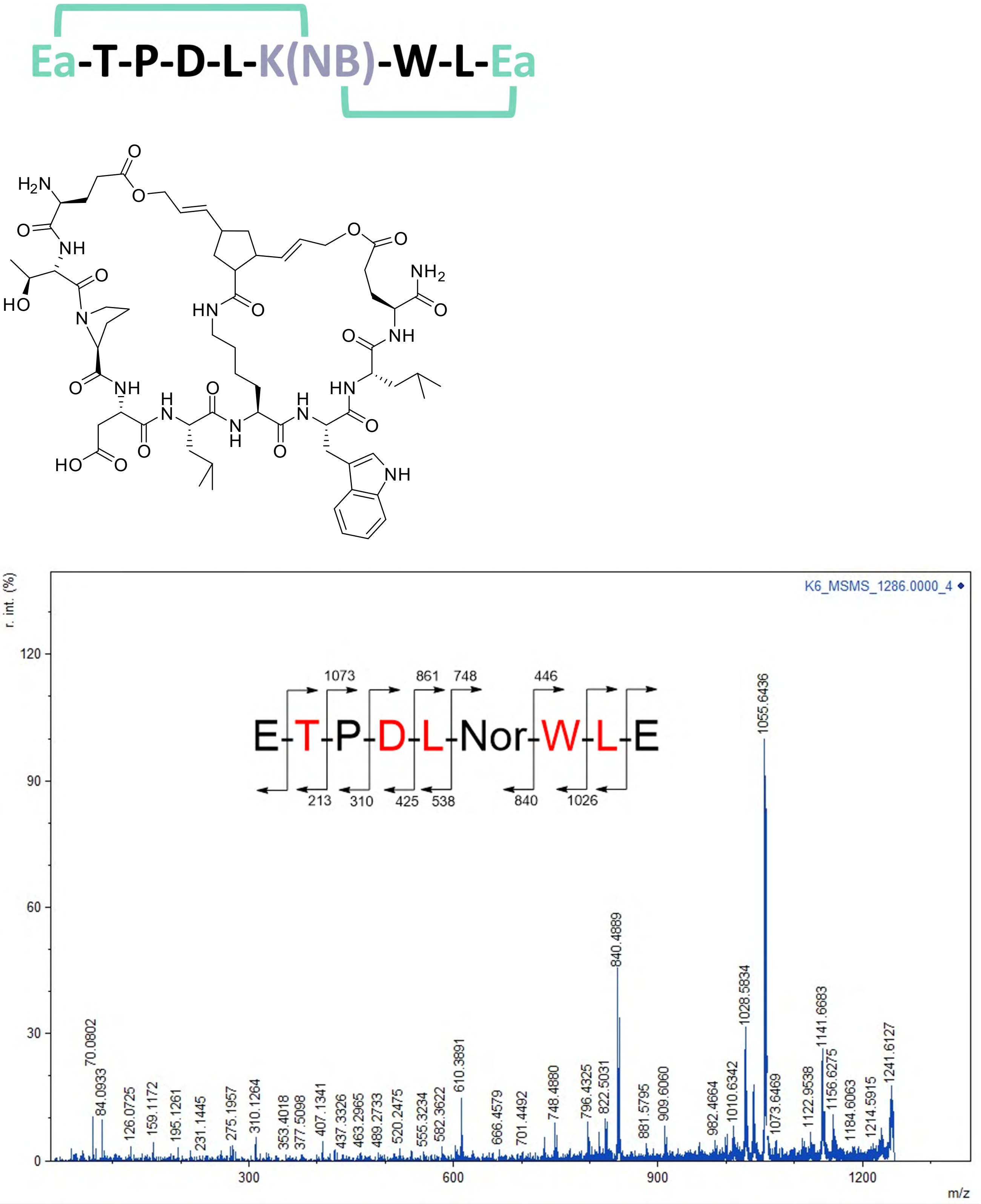

Figure S14. MS/MS of a random bead the from library, T-D-L-W-L. Parent ion: 1286.00.

Ea-F-P-D-A-K(NB)-D-D-Ea

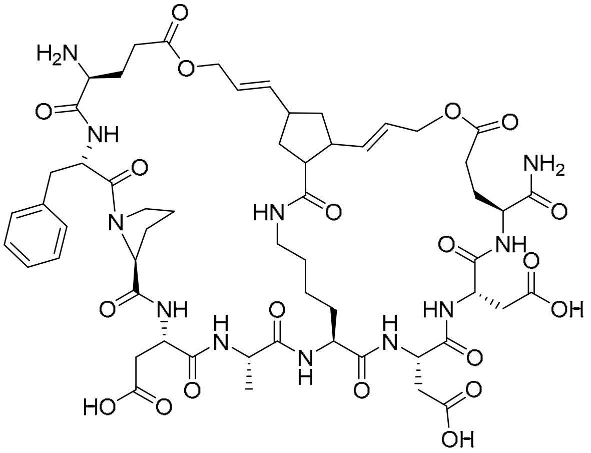

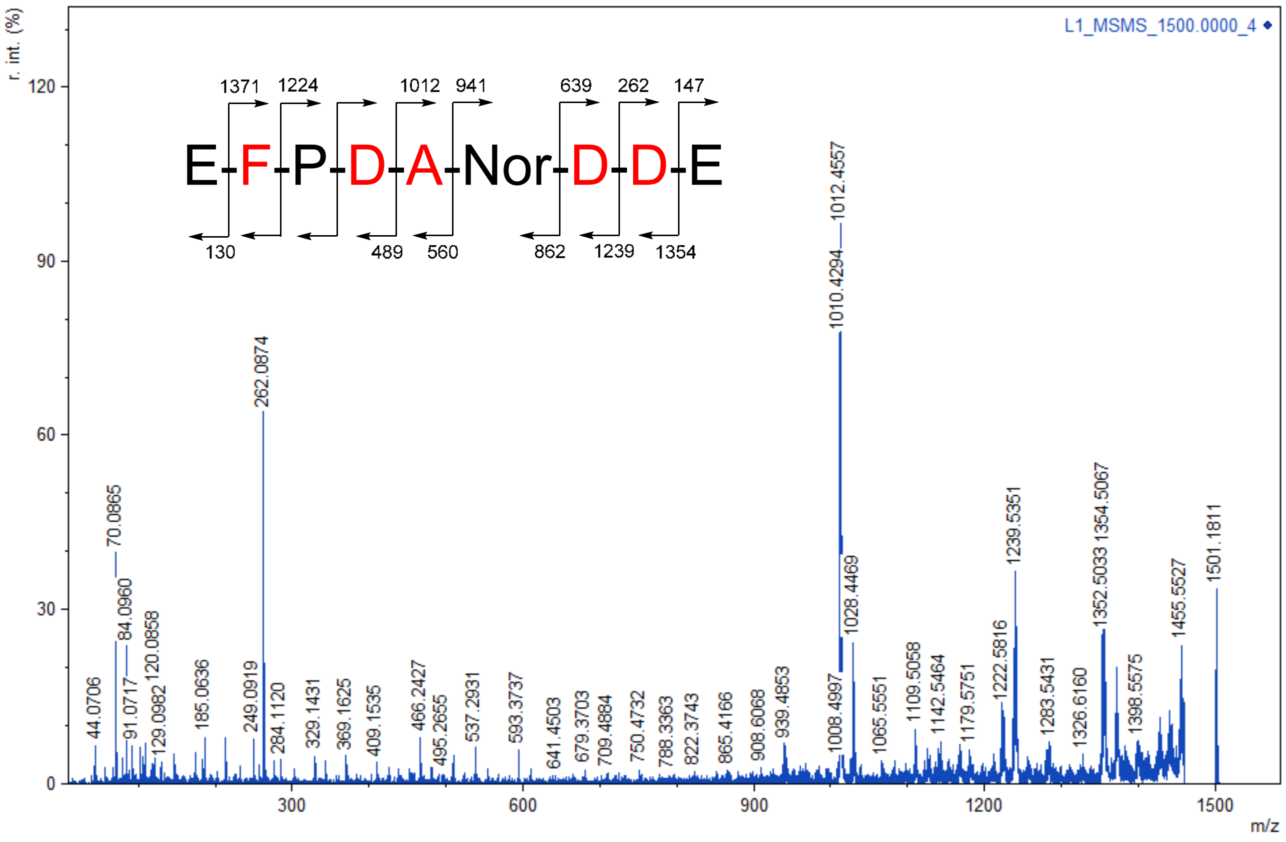

Figure S15. MS/MS of a random bead from the library, F-D-A-D-D. Parent ion: 1500.00.

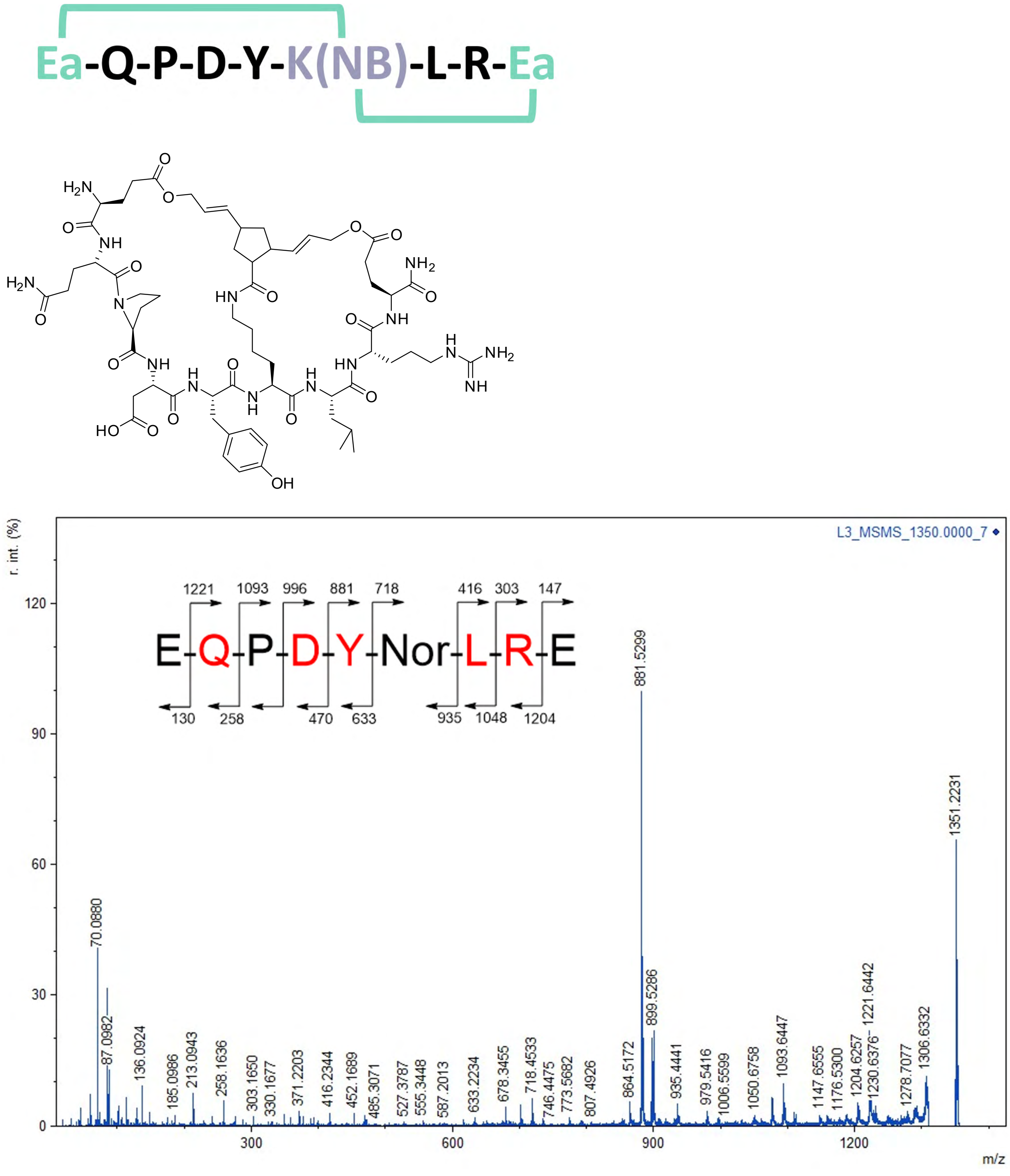

Figure S16. MS/MS of a random bead from the library, Q-D-Y-L-R. Parent ion: 1350.00.

Ea-D-P-P-K-K(NB)-S-E-Ea

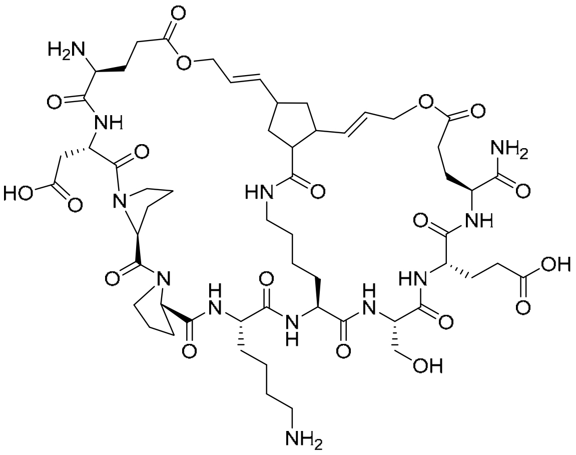

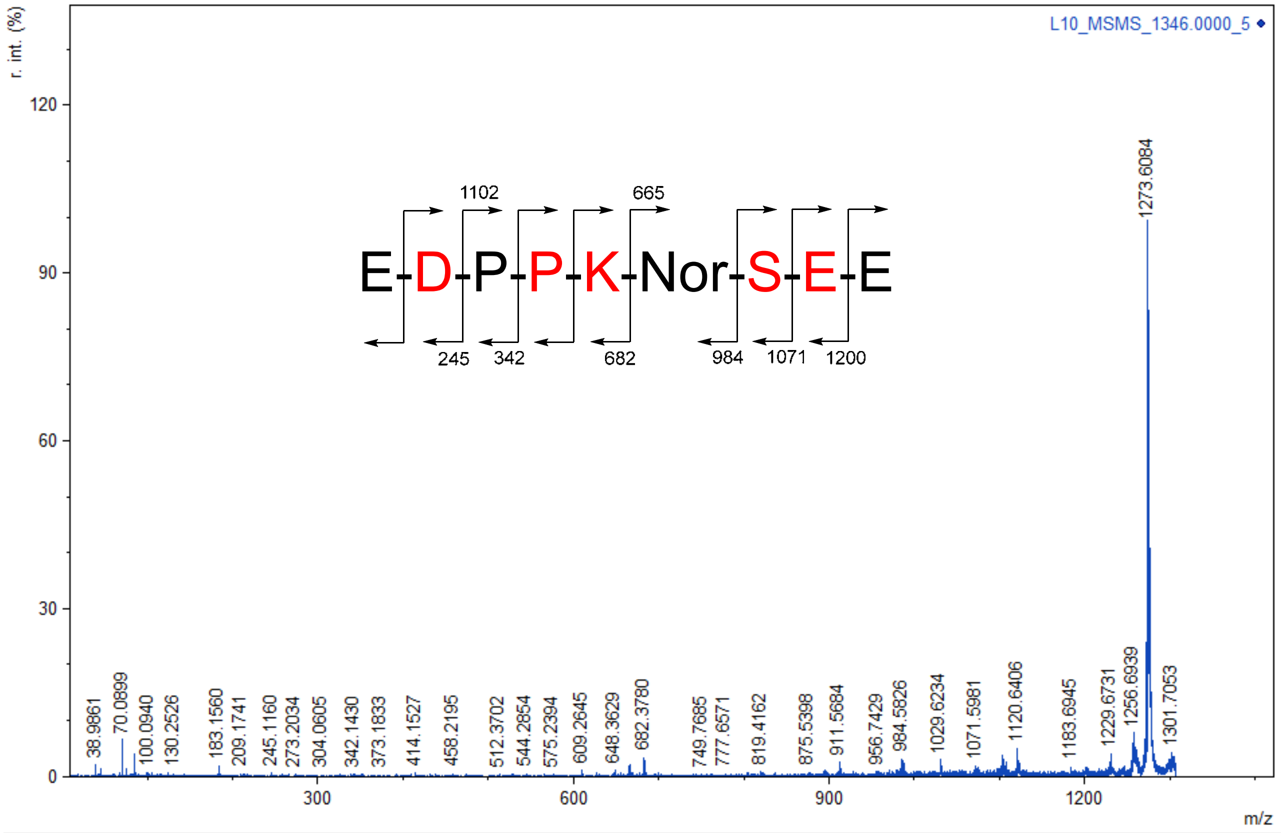

Figure S17. MS/MS of a random bead from the library, D-P-K-S-E. Parent ion: 1346.00.

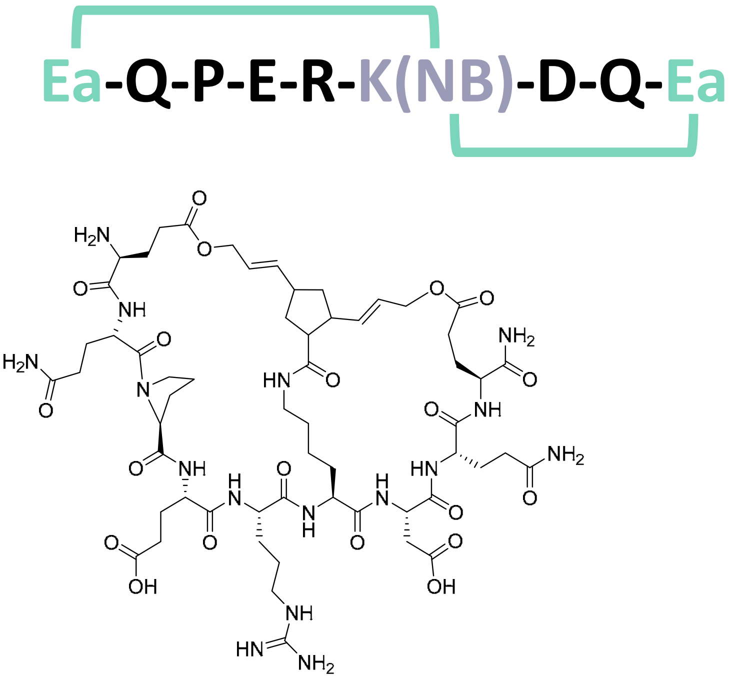

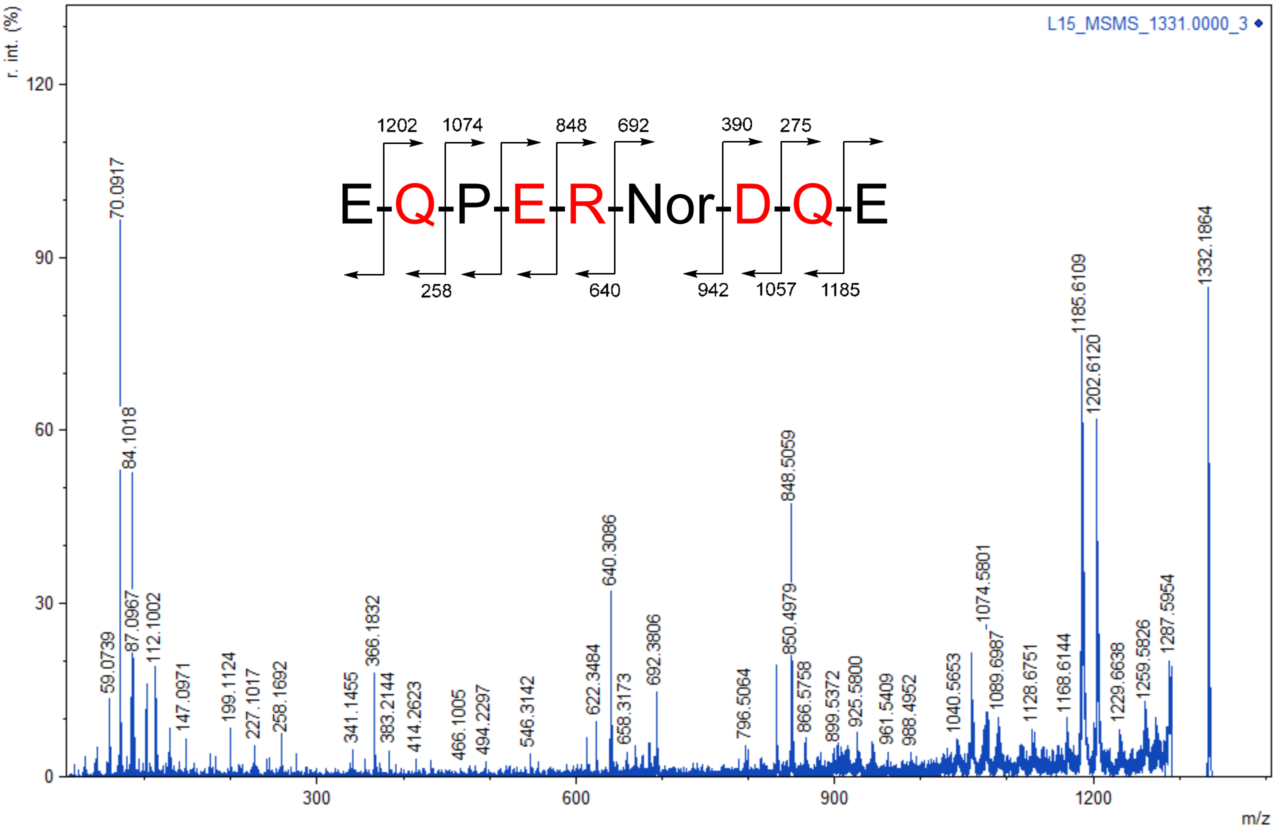

Figure S18. MS/MS of a random bead from the library, Q-E-R-D-Q. Parent ion: 1331.00.

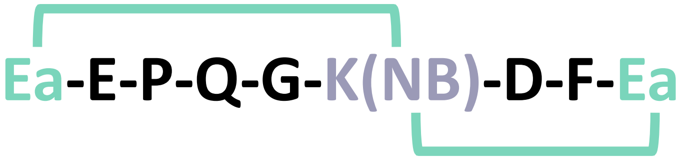

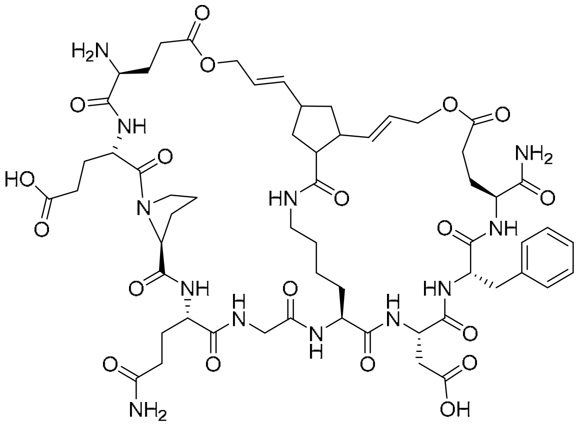

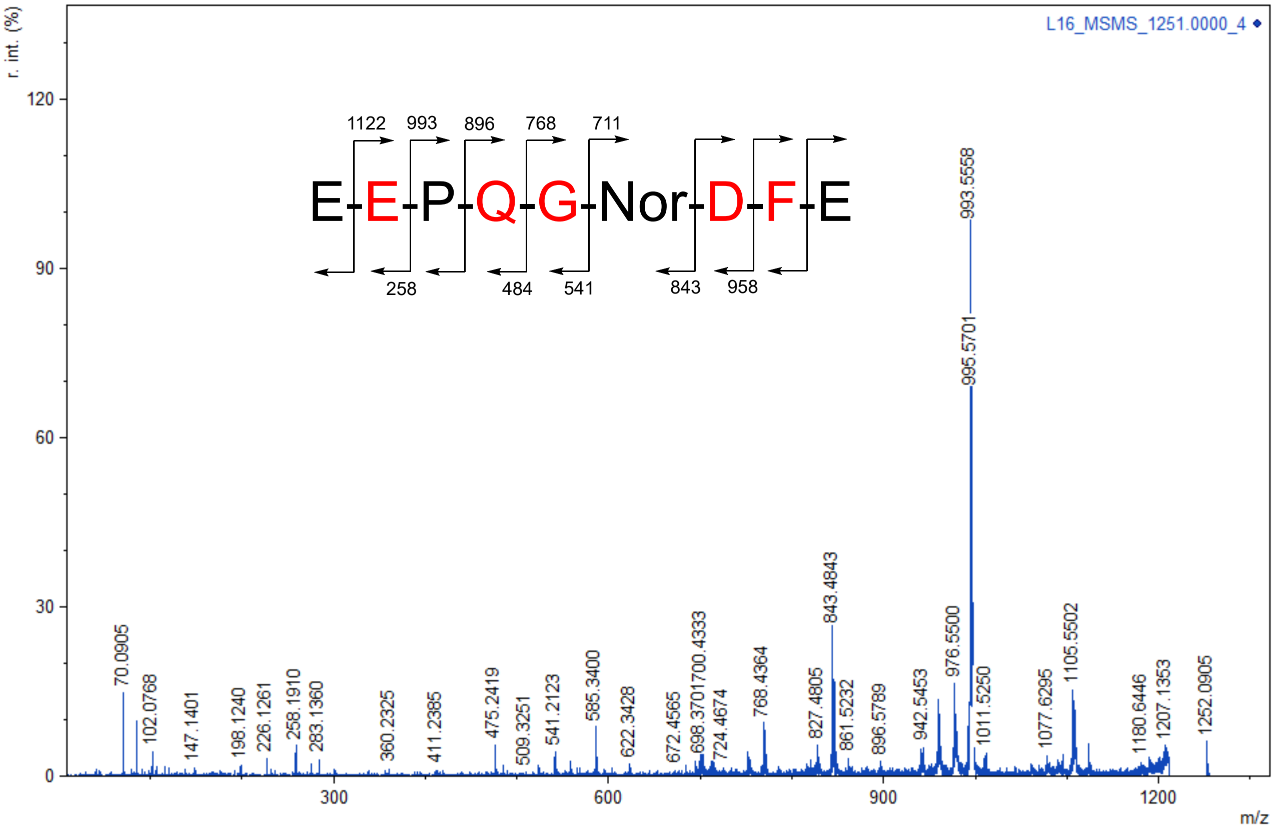

Figure S19. MS/MS of a random bead from the library, E-Q-G-D-F. Parent ion: 1251.00.

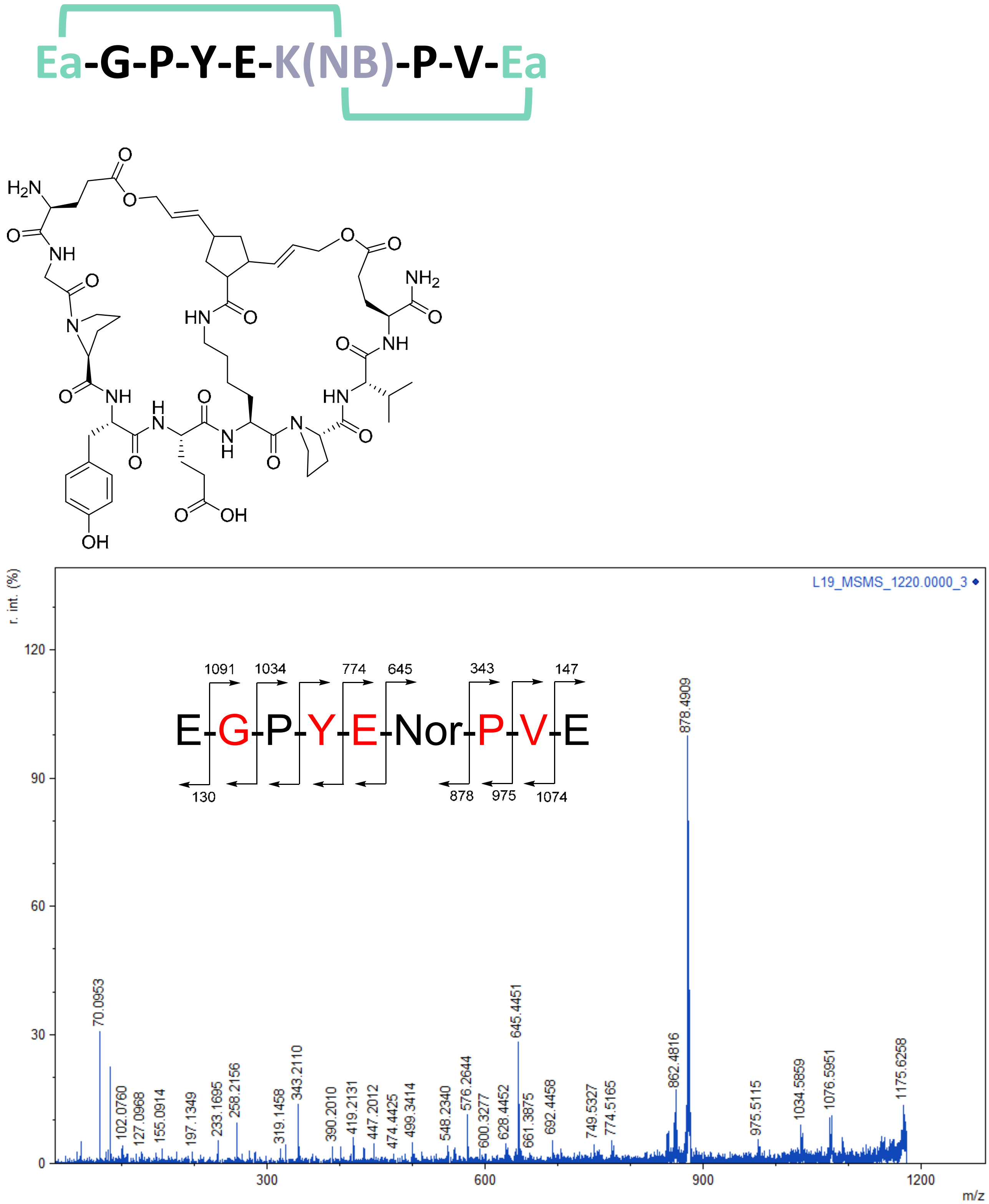

Figure S20. MS/MS of a random bead from the library, G-Y-E-P-V. Parent ion: 1220.00.

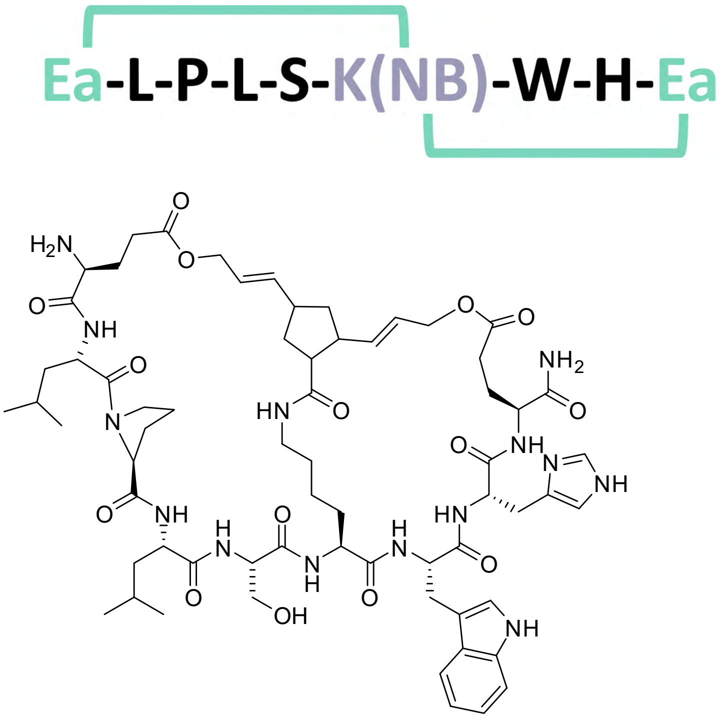

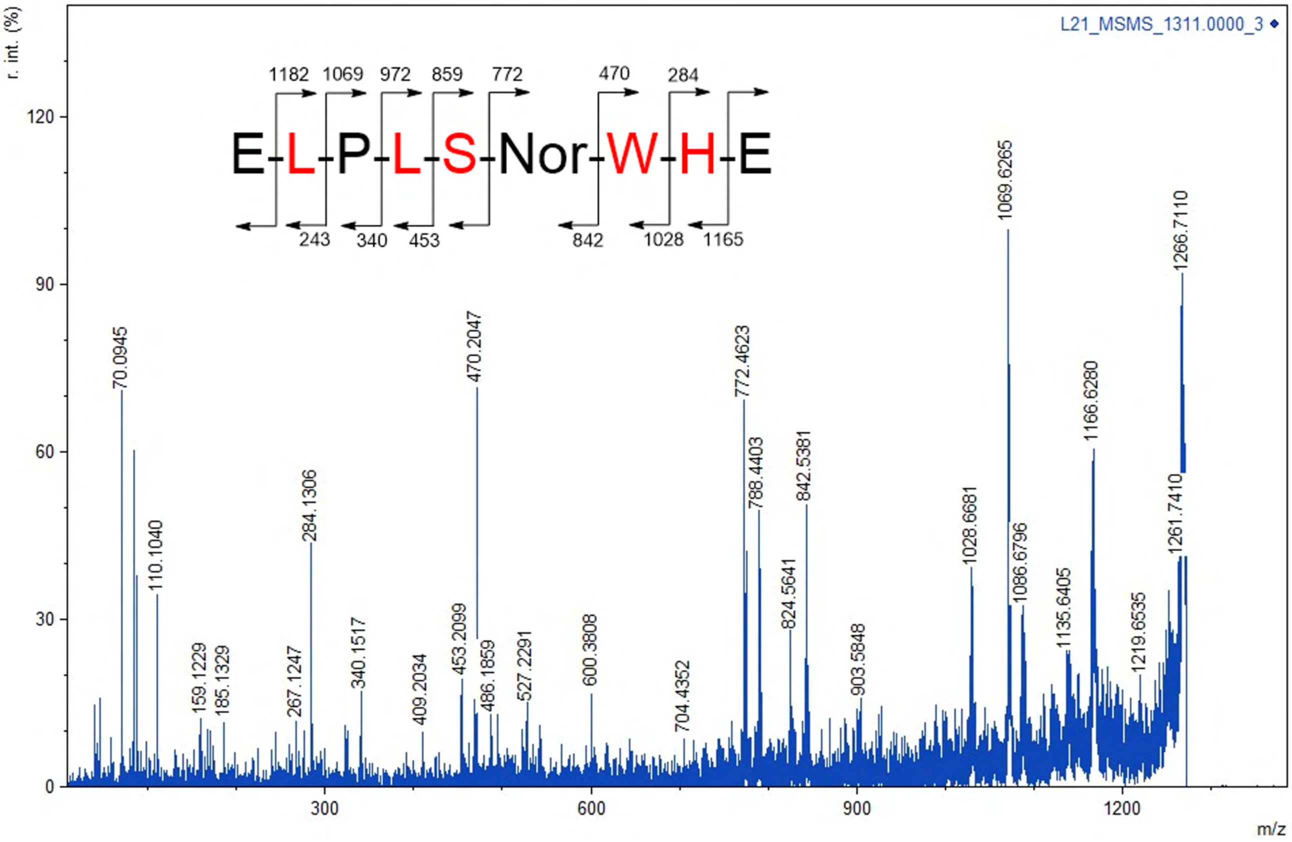

Figure S21. MS/MS of a random bead from the library, L-L-S-W-H. Parent ion: 1311.00.

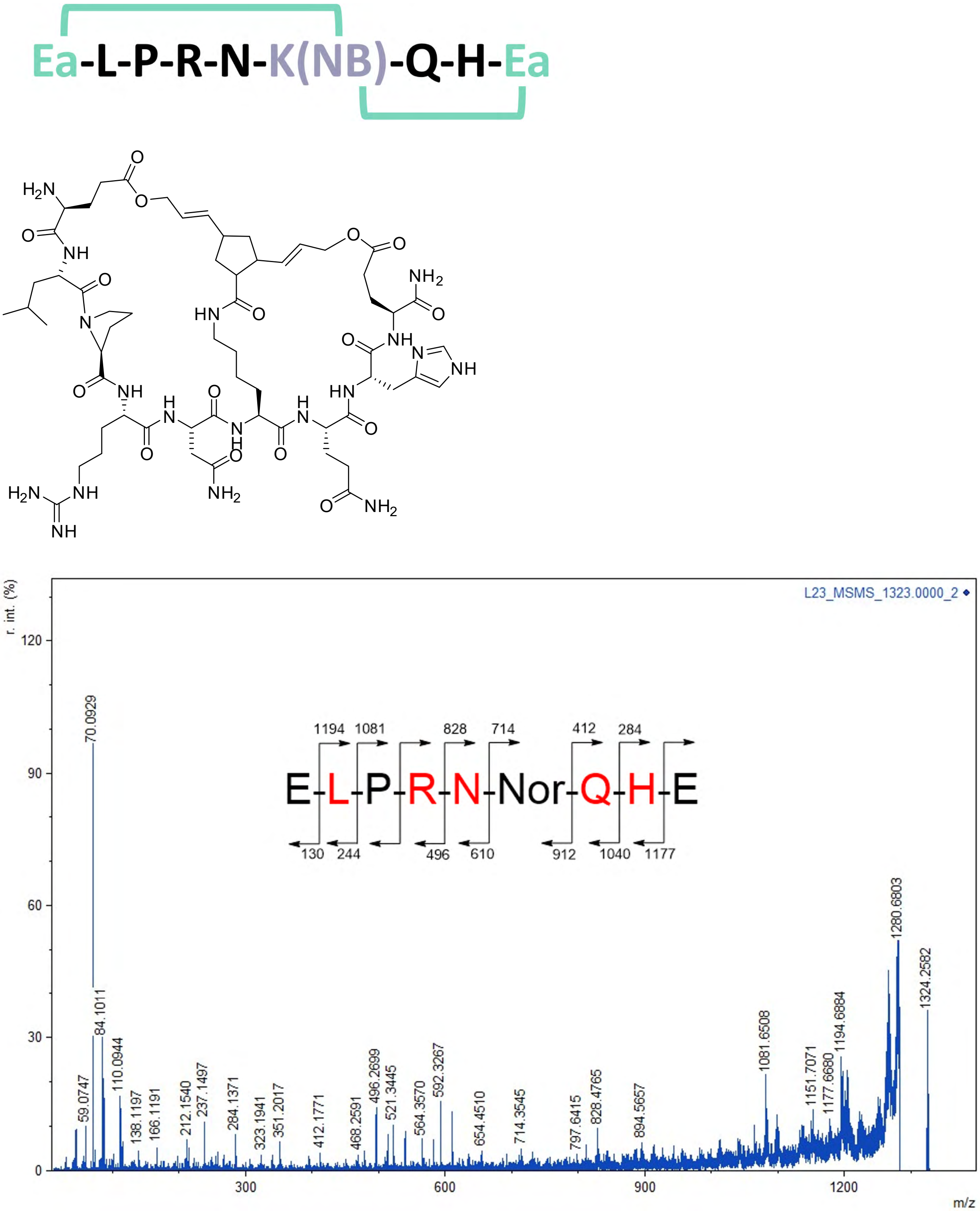

Figure S22. MS/MS of a random bead from the library, L-R-N-Q-H. Parent ion: 1323.00.

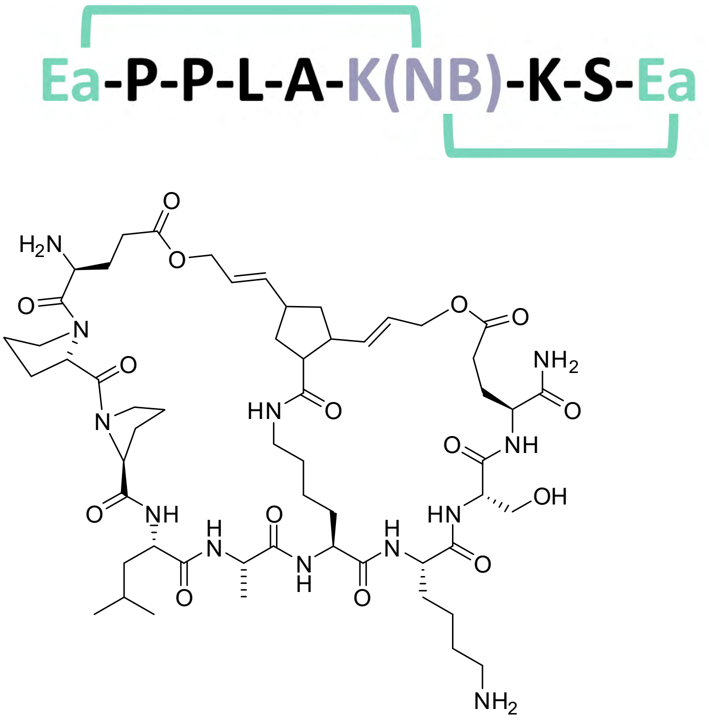

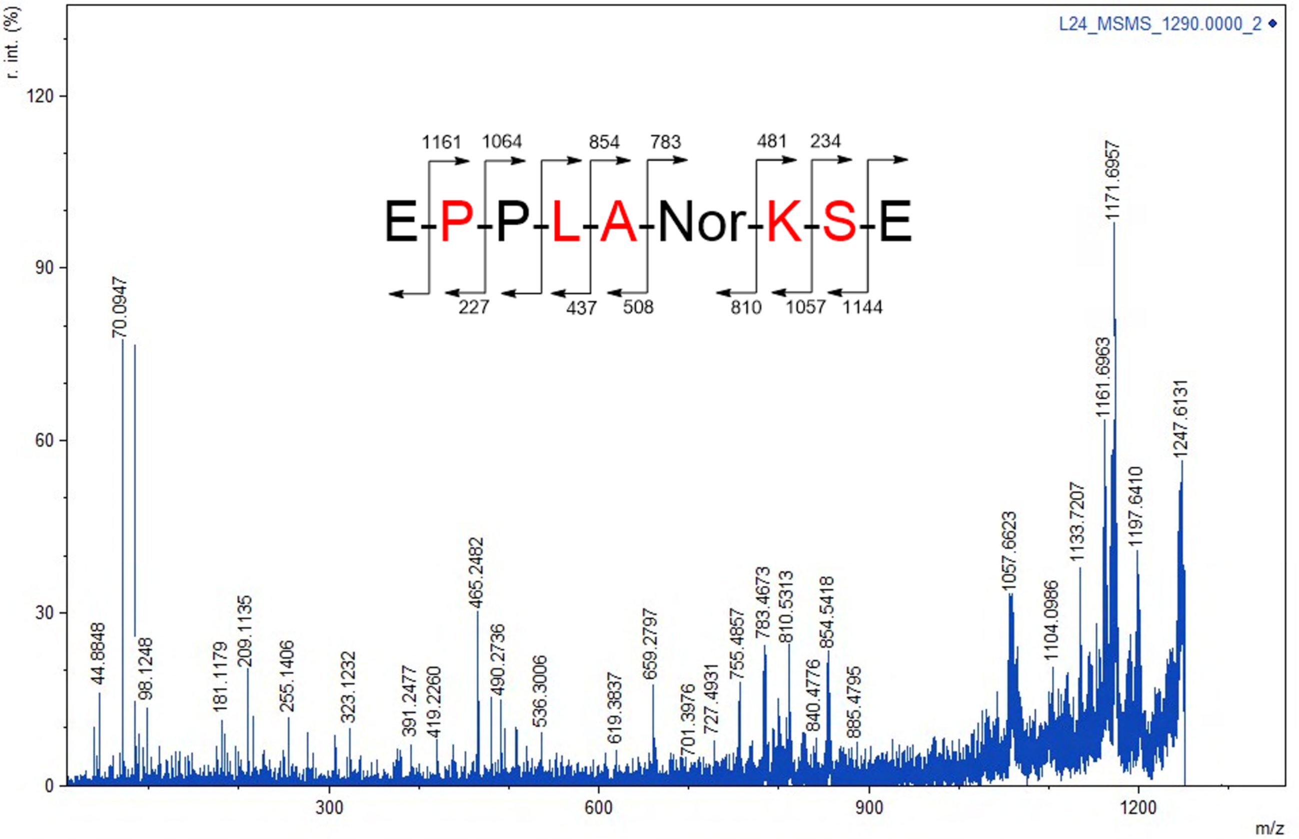

Figure S23. MS/MS of a random bead from the library, P-L-A-K-S. Parent ion: 1290.00.

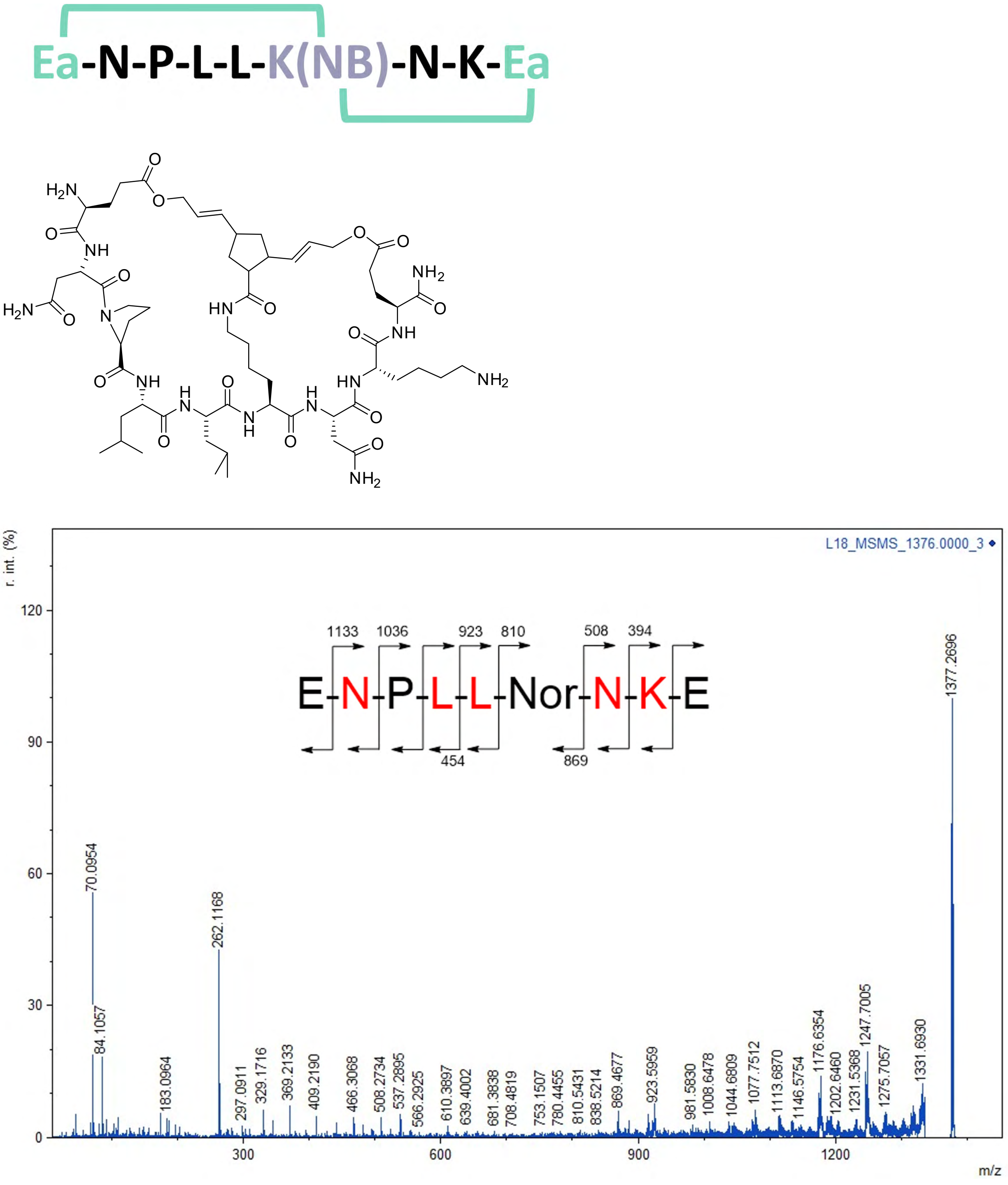

Figure S24. MS/MS of a random bead from the library, N-L-L-N-K. Parent ion: 1376.00.

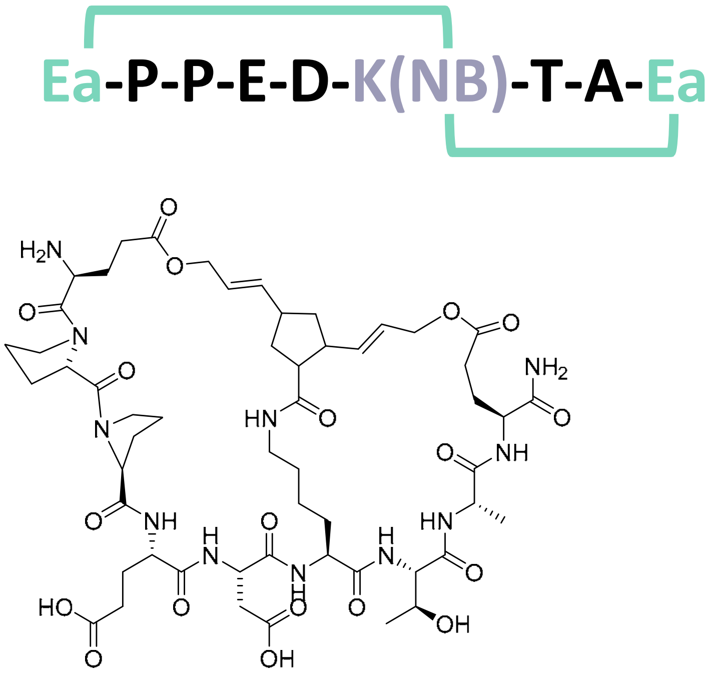

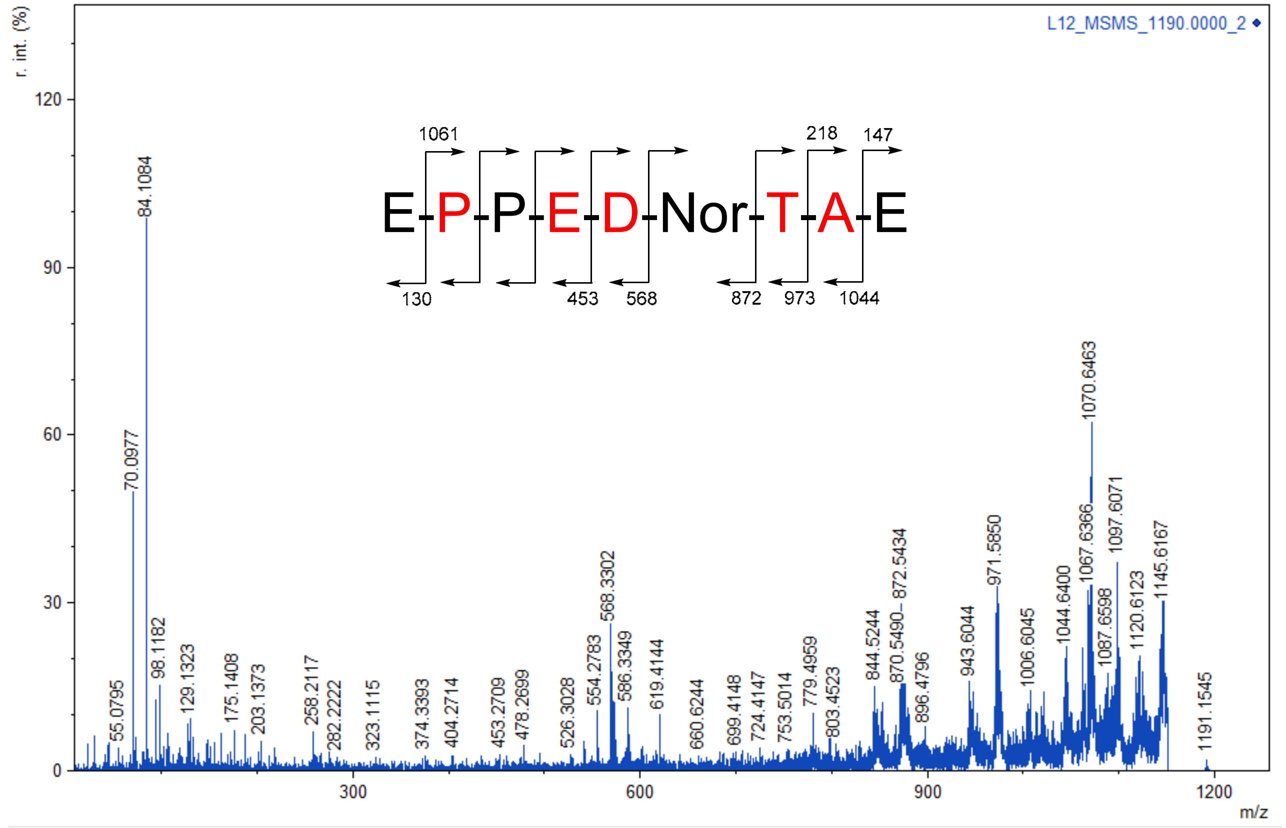

Figure S25. MS/MS of a random bead from the library, P-E-D-T-A. Parent ion: 1190.00.

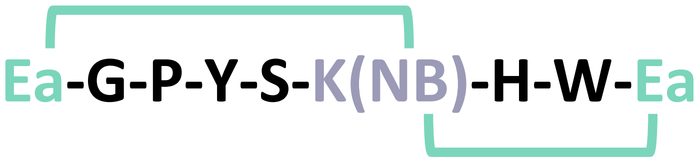

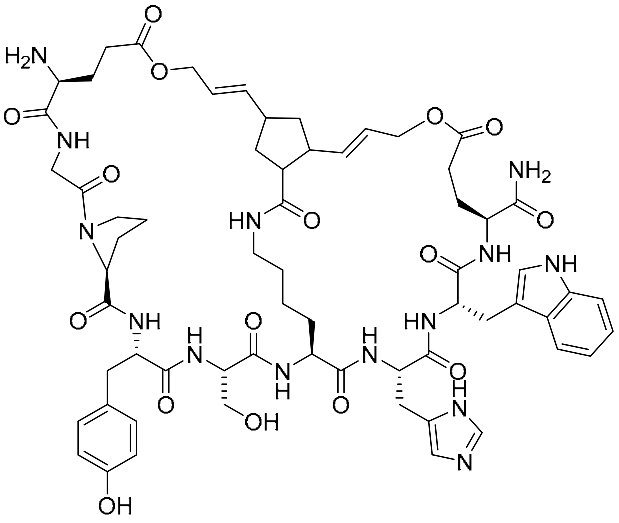

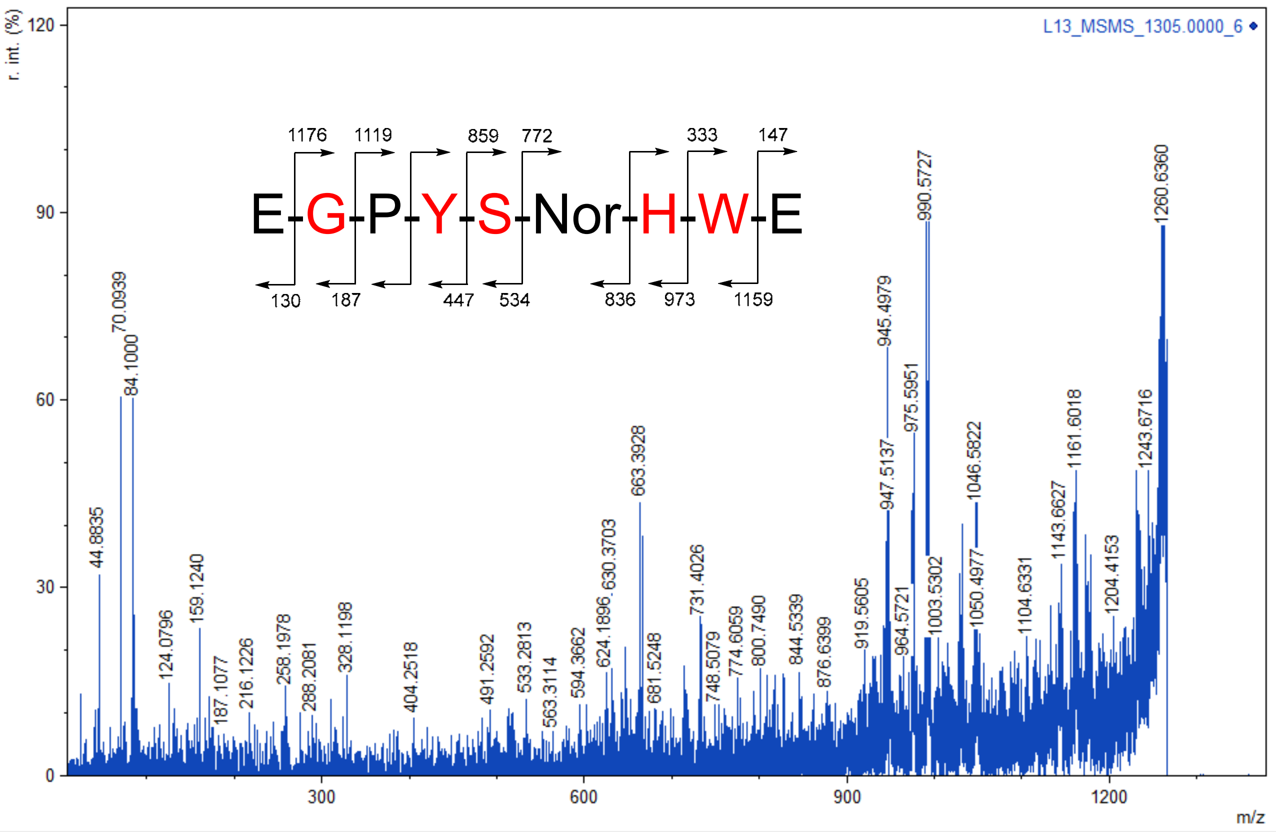

Figure S26. MS/MS of a random bead from the library, G-Y-S-H-W. Parent ion: 1305.00.

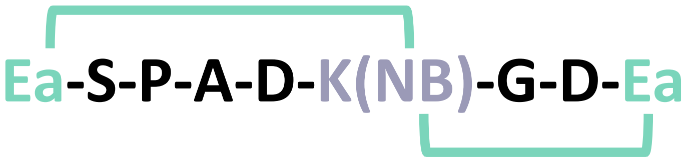

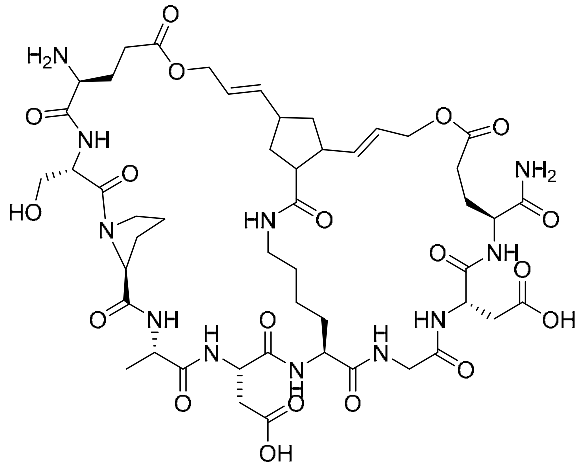

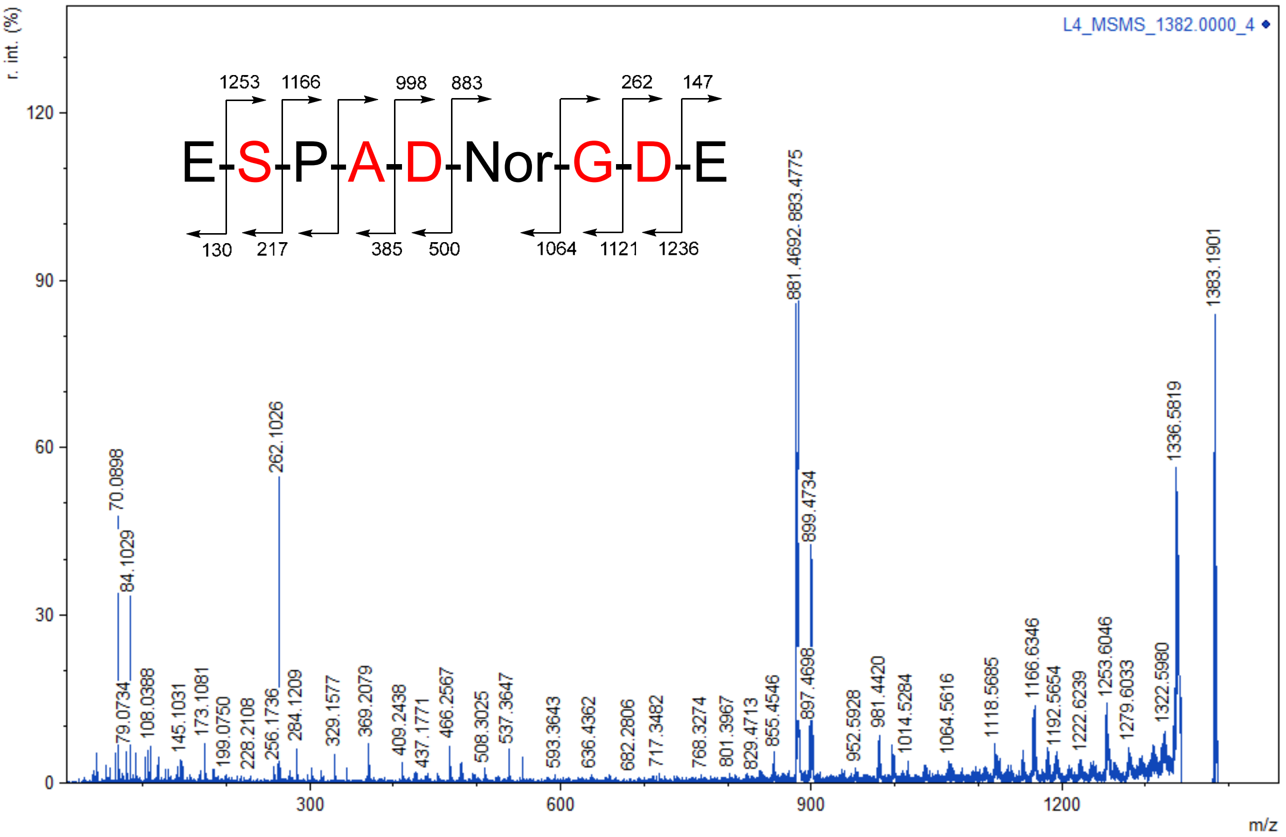

Figure S27. MS/MS of a random bead from the library, S-A-D-G-D. Parent ion: 1382.00.

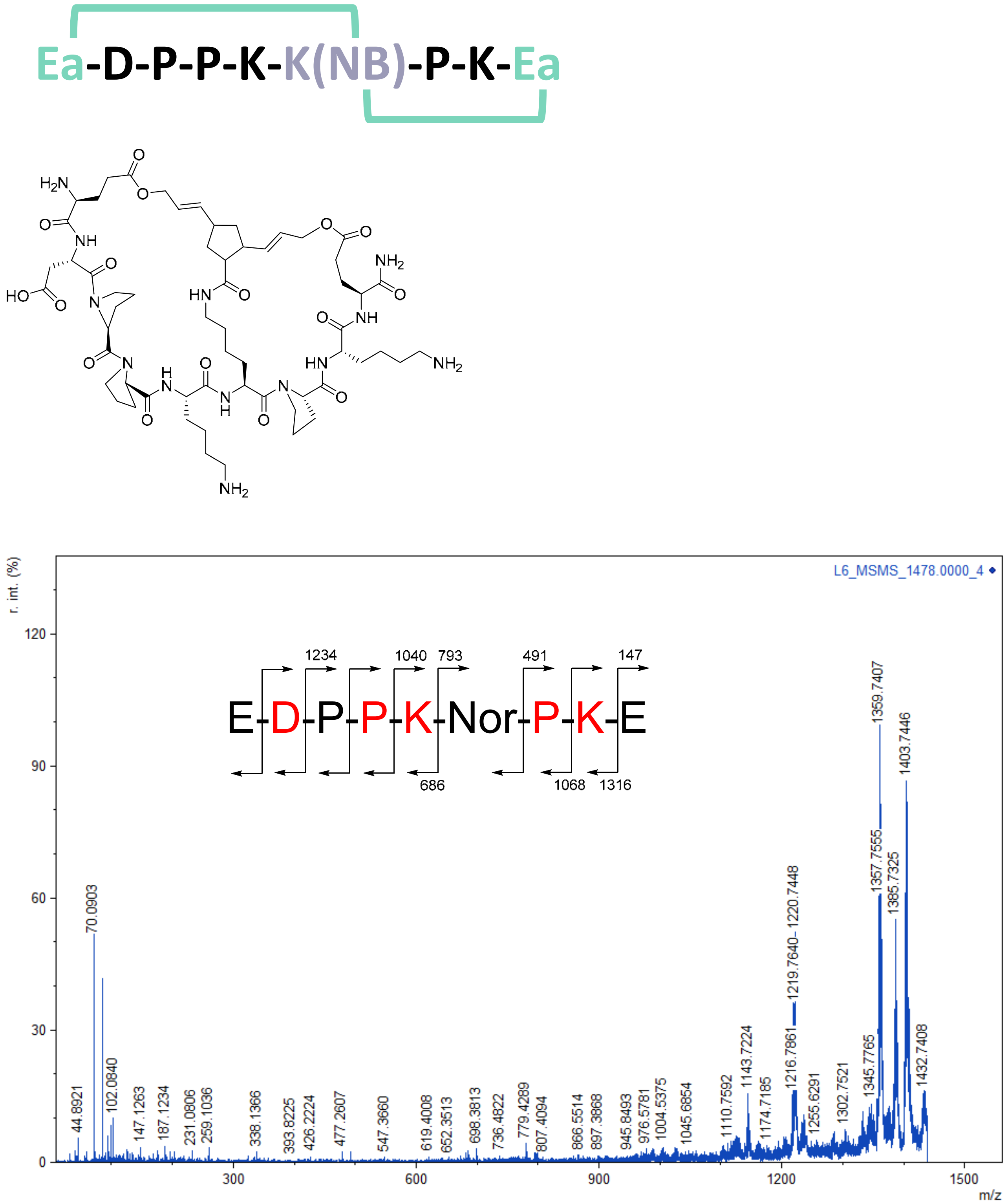

Figure S28. MS/MS of a random bead from the library, D-P-K-P-K. Parent ion: 1478.00.

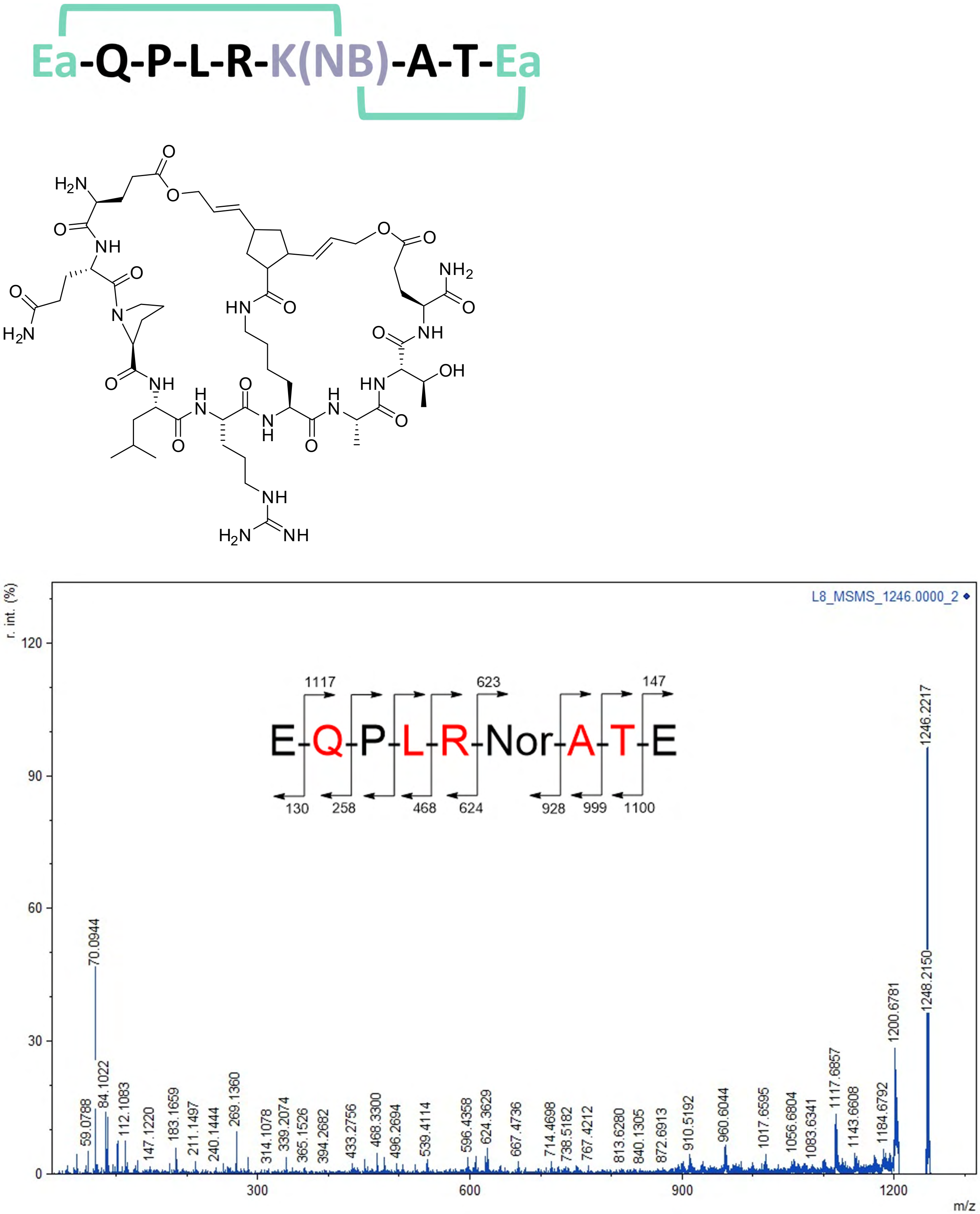

Figure S29. MS/MS of a random bead from the library, Q-L-R-A-T. Parent ion: 1246.00.

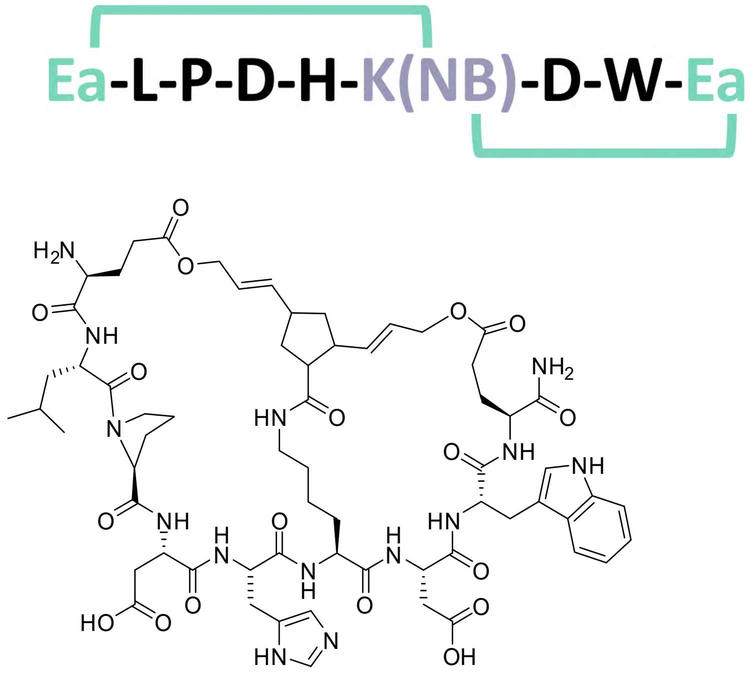

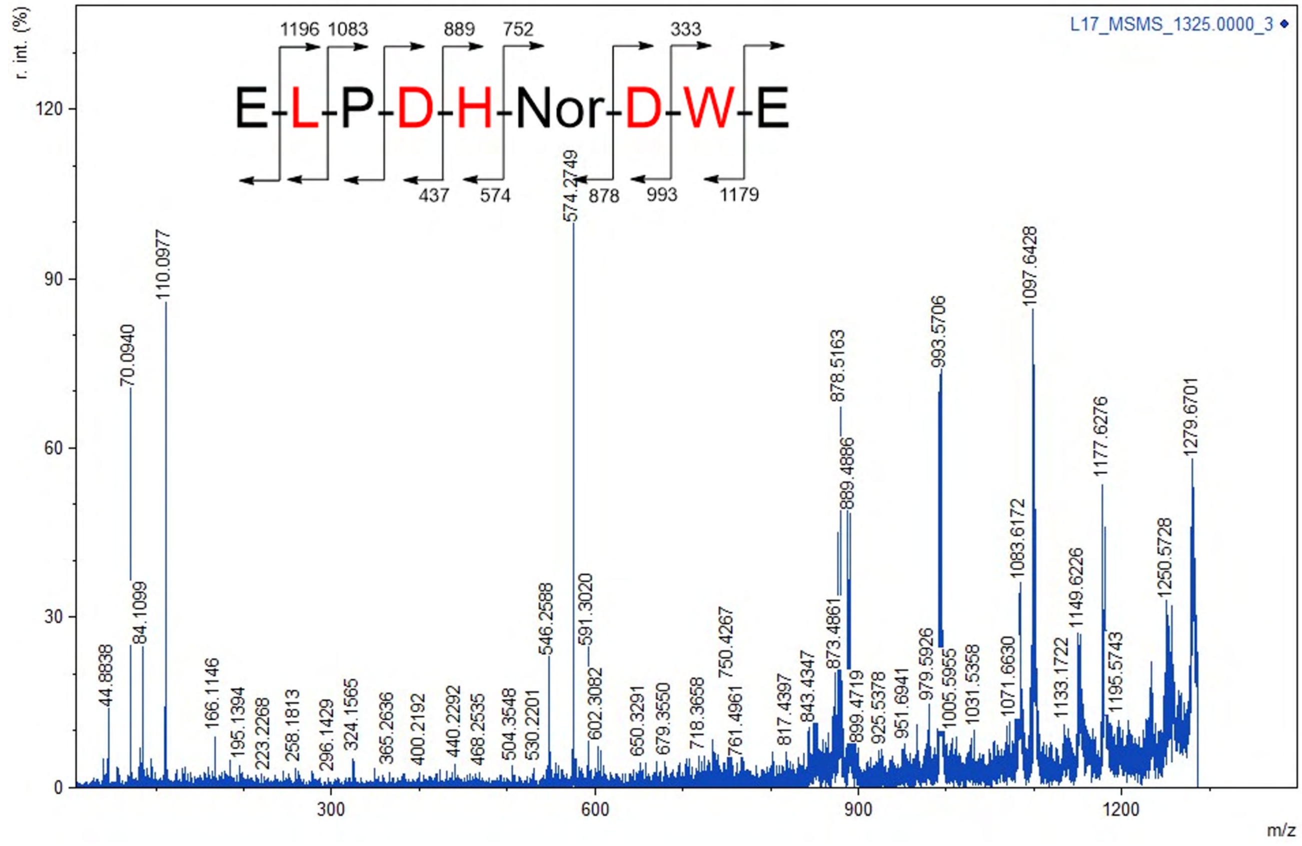

Figure S30. MS/MS of a random bead from the library, L-D-H-D-W. Parent ion: 1325.00.

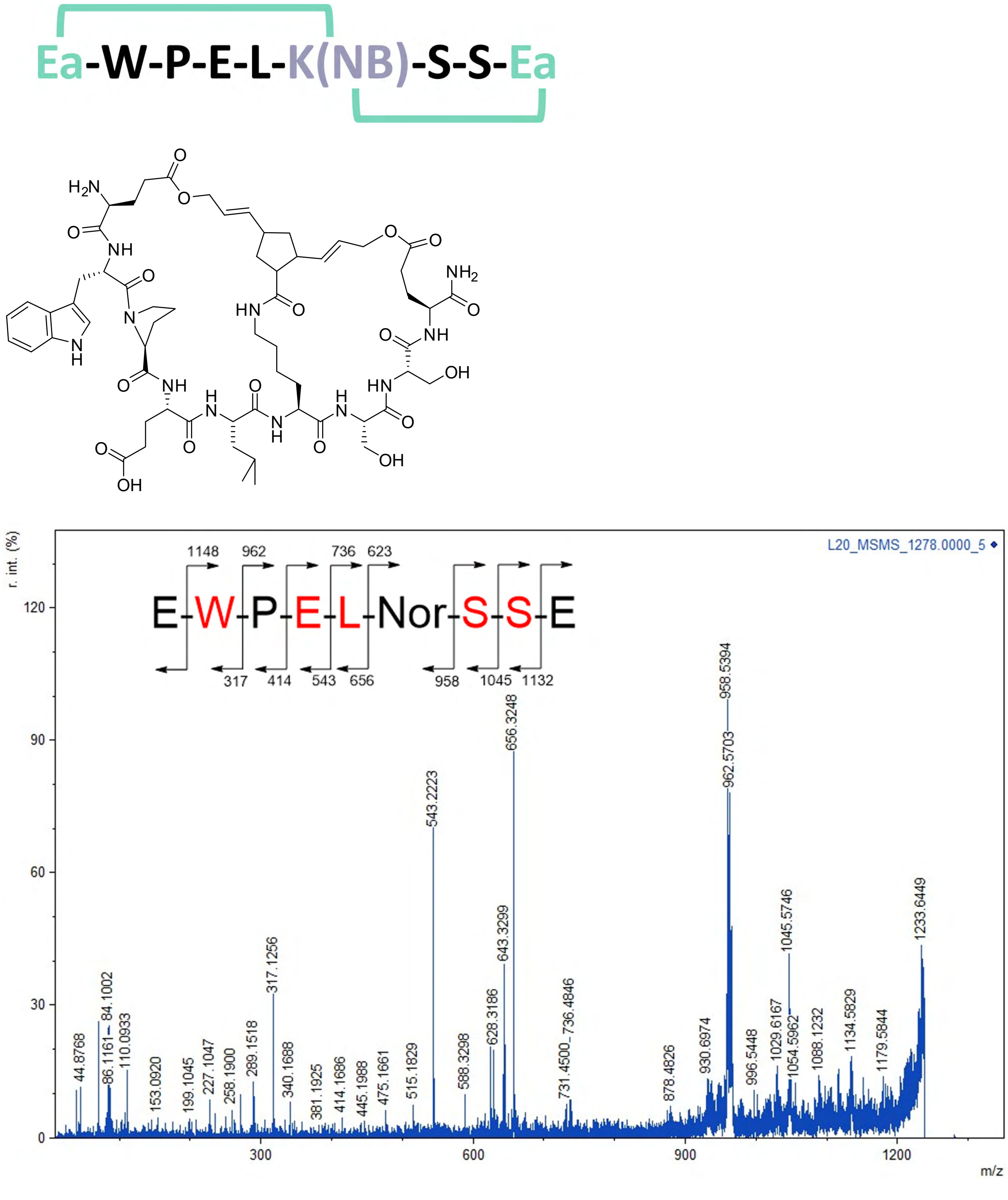

Figure S31. MS/MS of a random bead from the library, W-E-L-S-S. Parent ion: 1278.00.

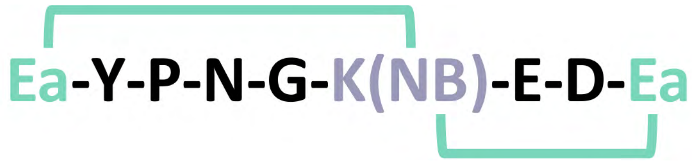

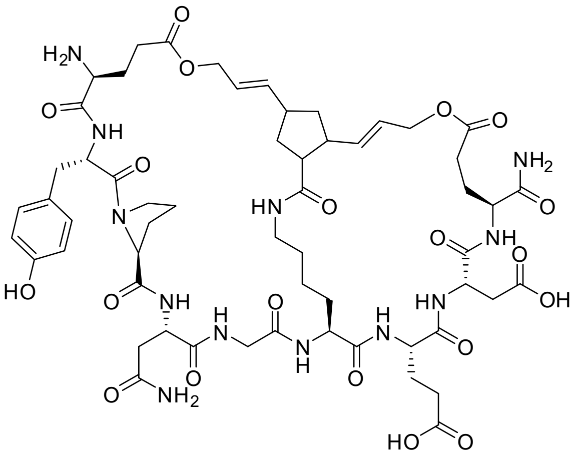

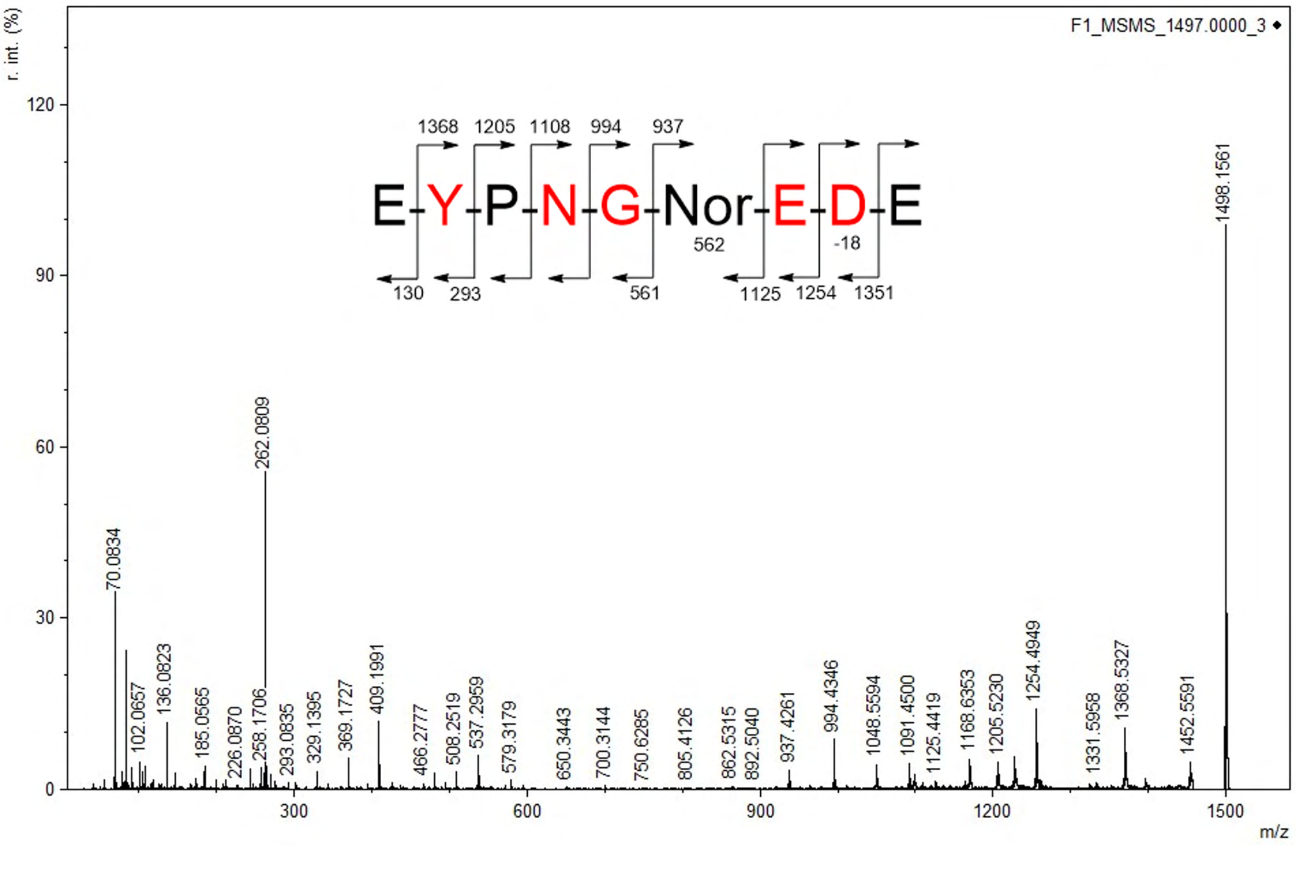

Figure S32. MS/MS of a random bead from the library, Y-N-G-E-D. Parent ion: 1498.15.

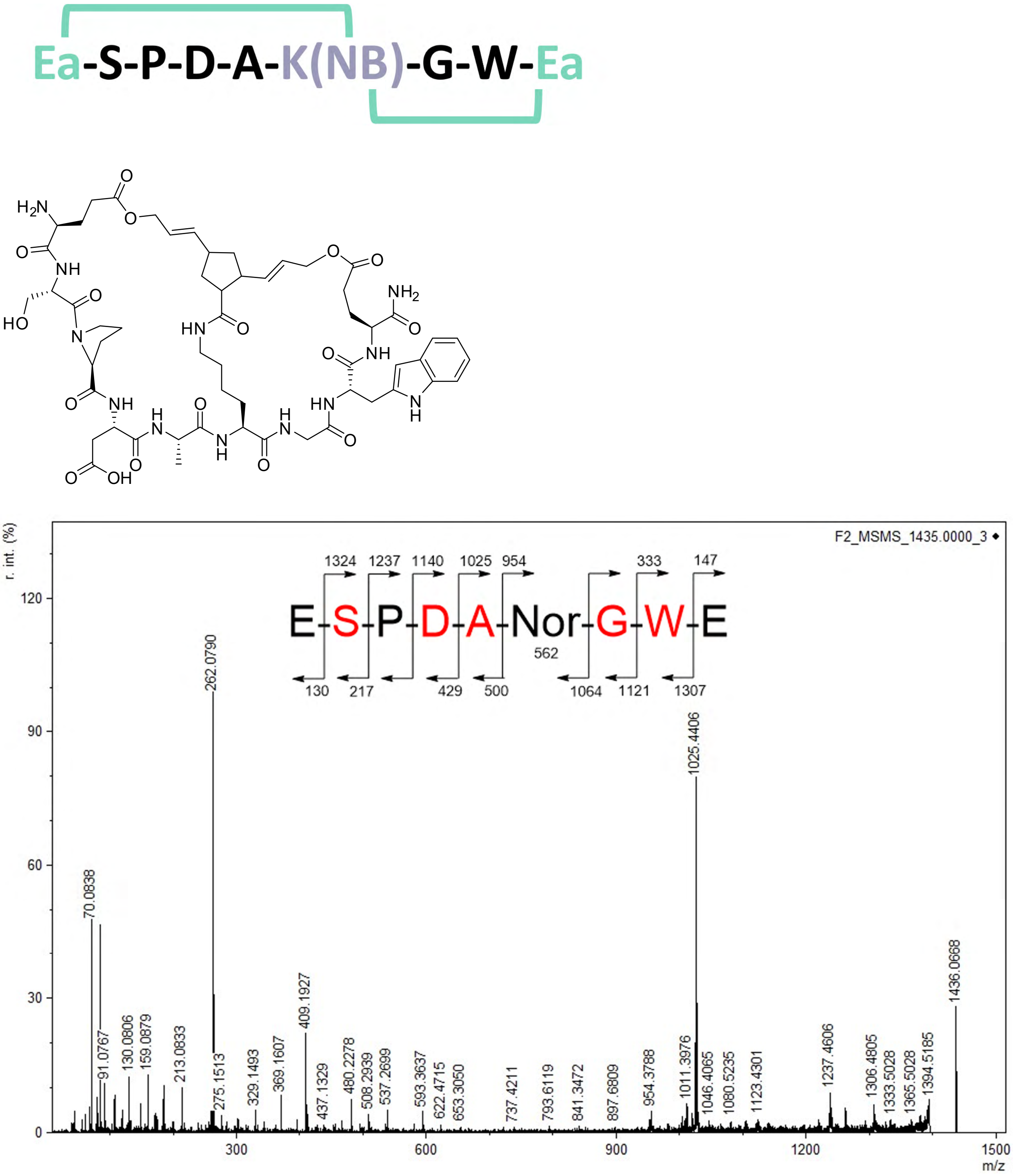

Figure S33. MS/MS of a random bead from the library, S-D-A-G-W. Parent ion: 1436.07.

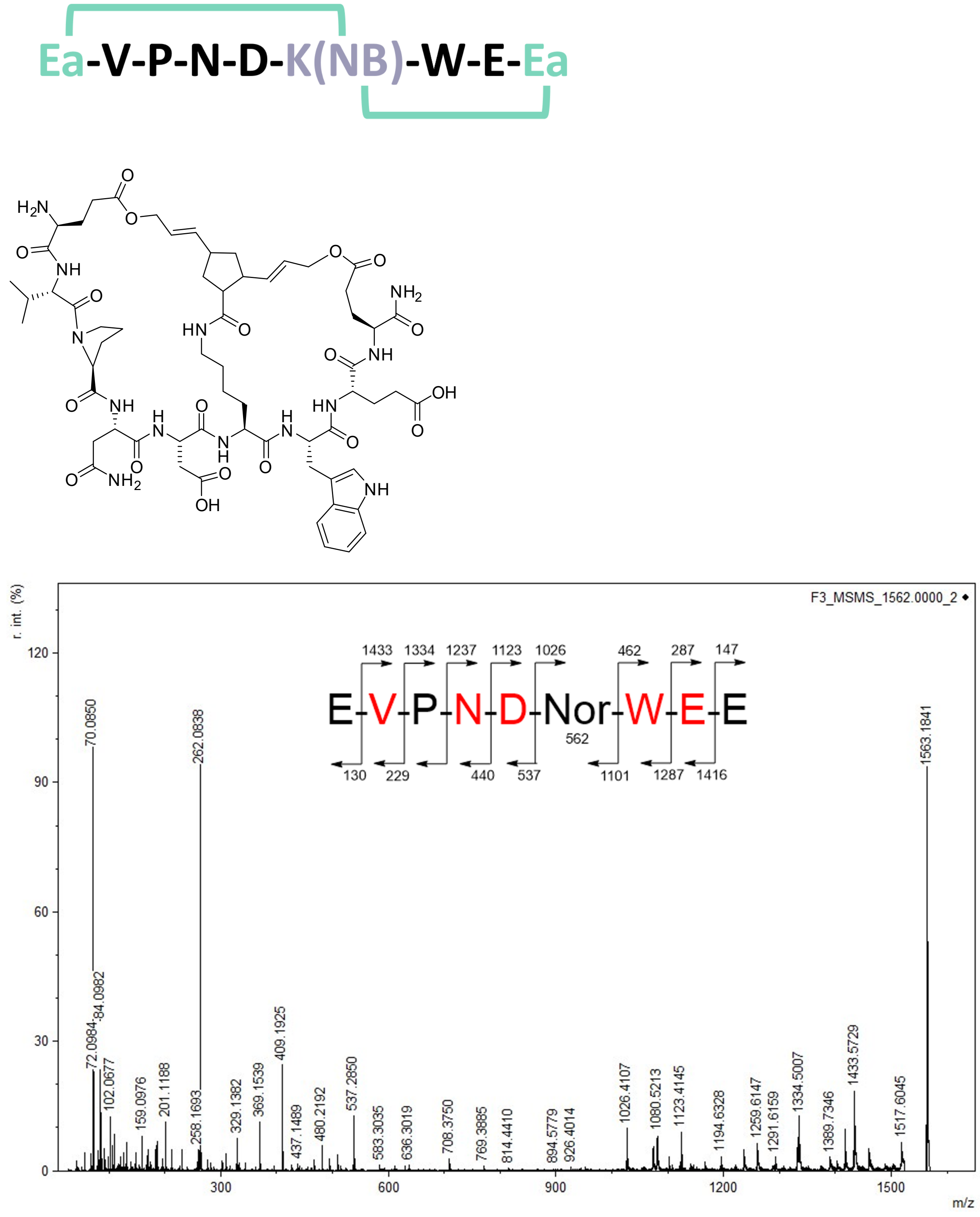

Figure S34. MS/MS of a random bead from the library, V-N-D-W-E. Parent ion: 1563.18.

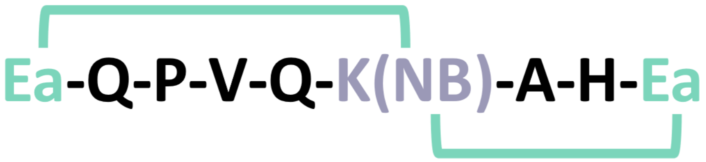

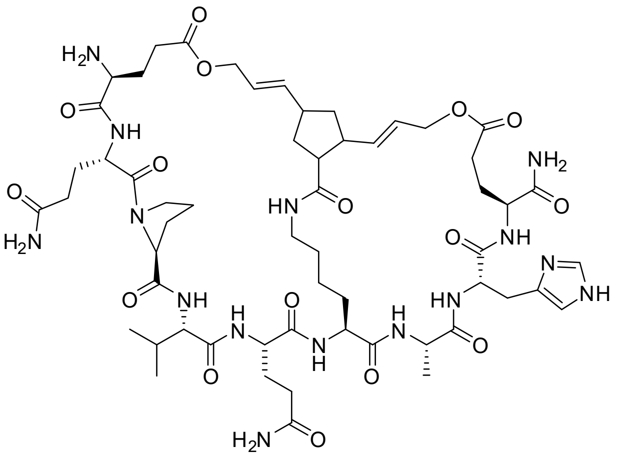

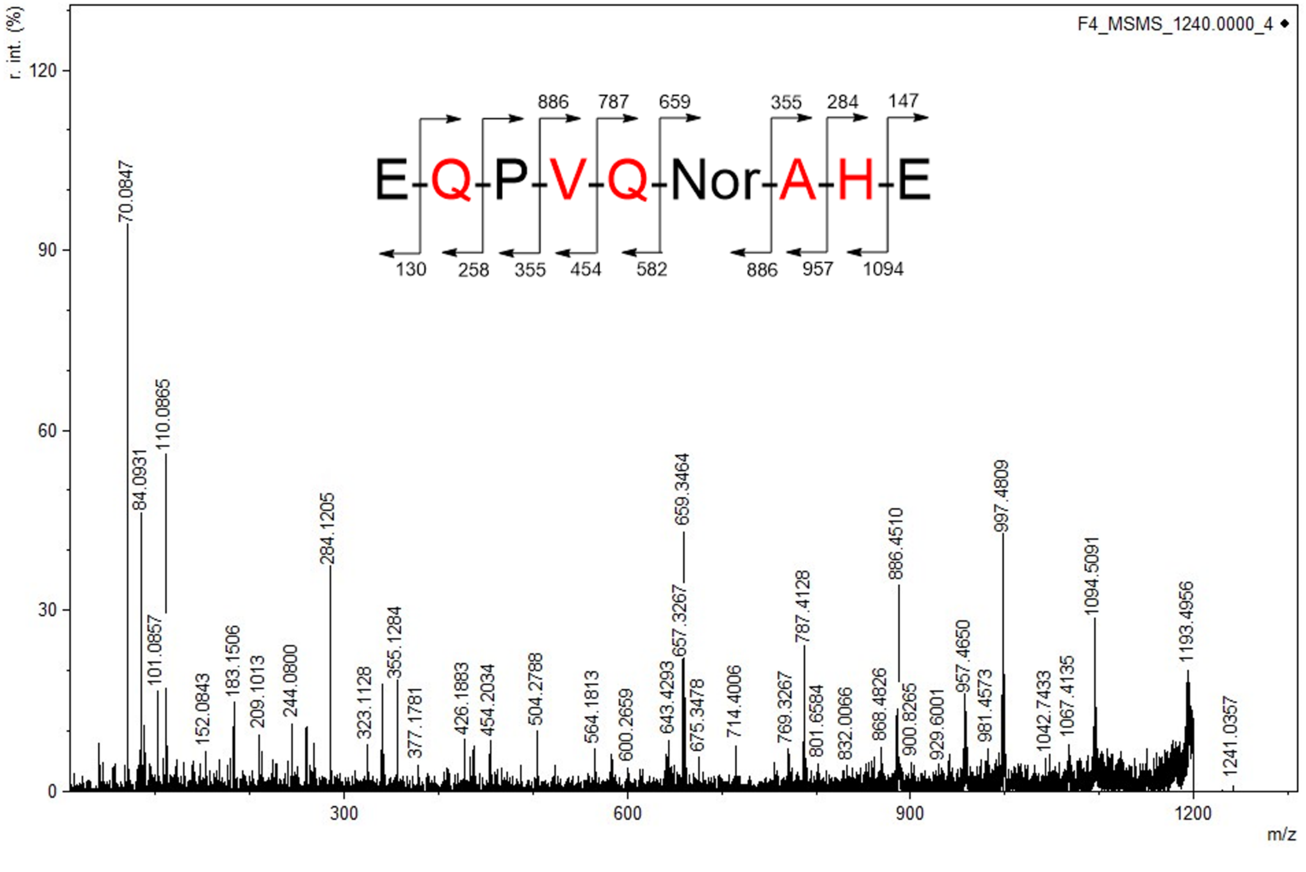

Figure S35. MS/MS of a random bead from the library, Q-V-Q-A-H. Parent ion: 1240.00.

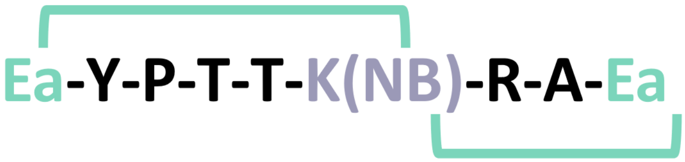

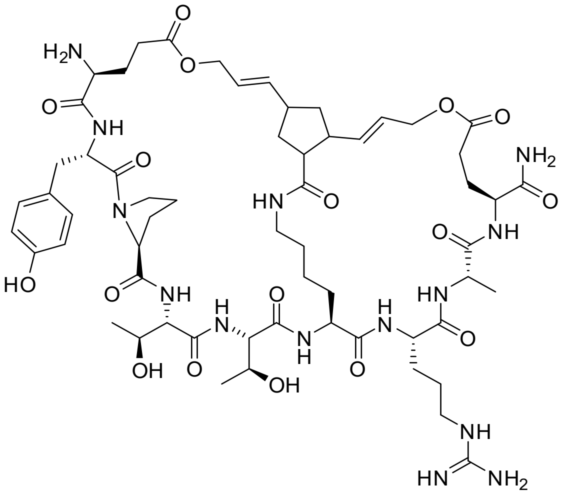

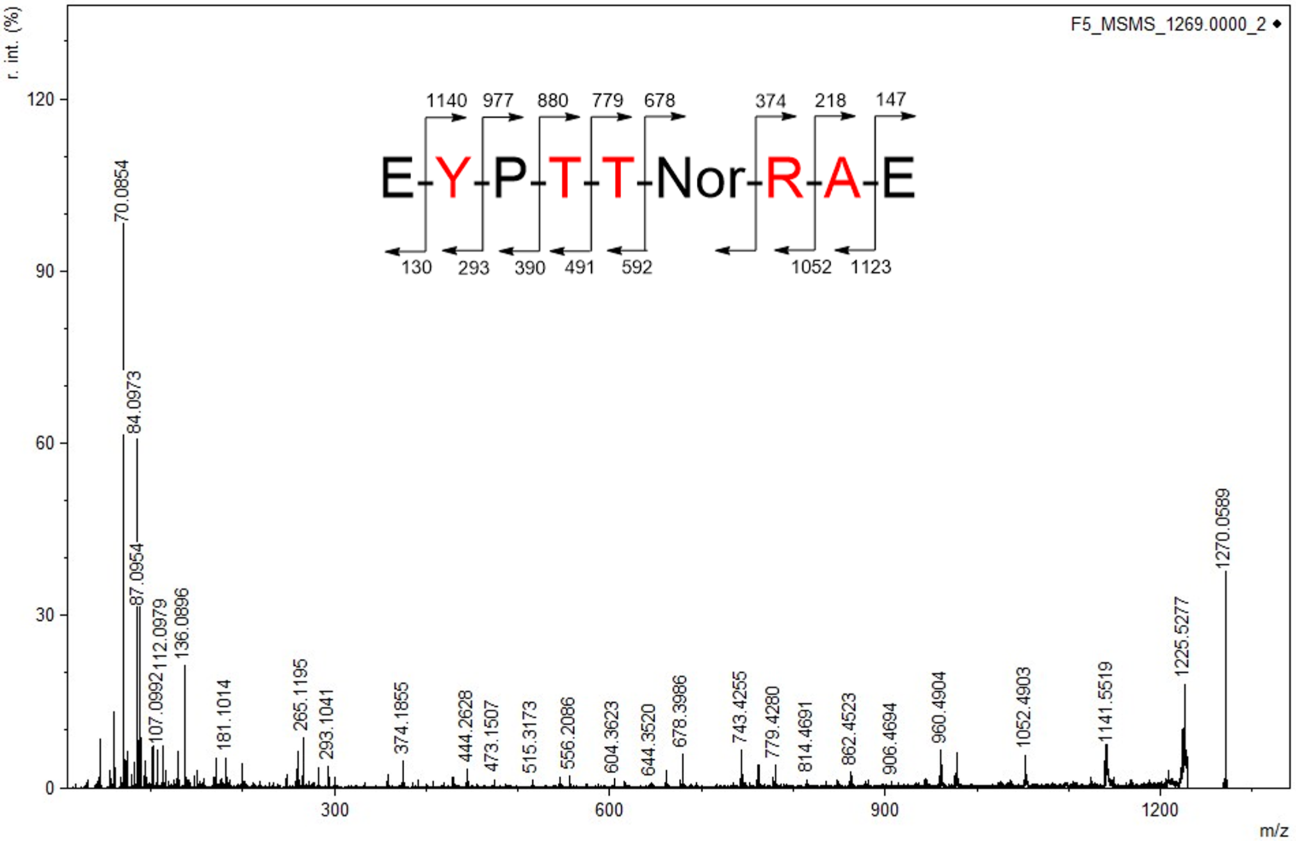

Figure S36. MS/MS of a random bead from the library, Y-T-T-R-A. Parent ion: 1269.00.

Ea-E-P-D-G-K(NB)-N-D-Ea

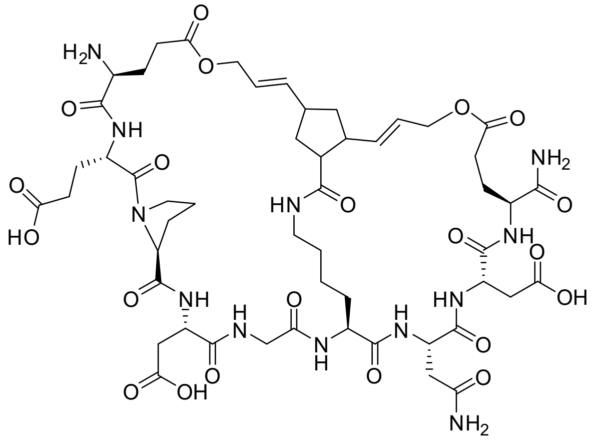

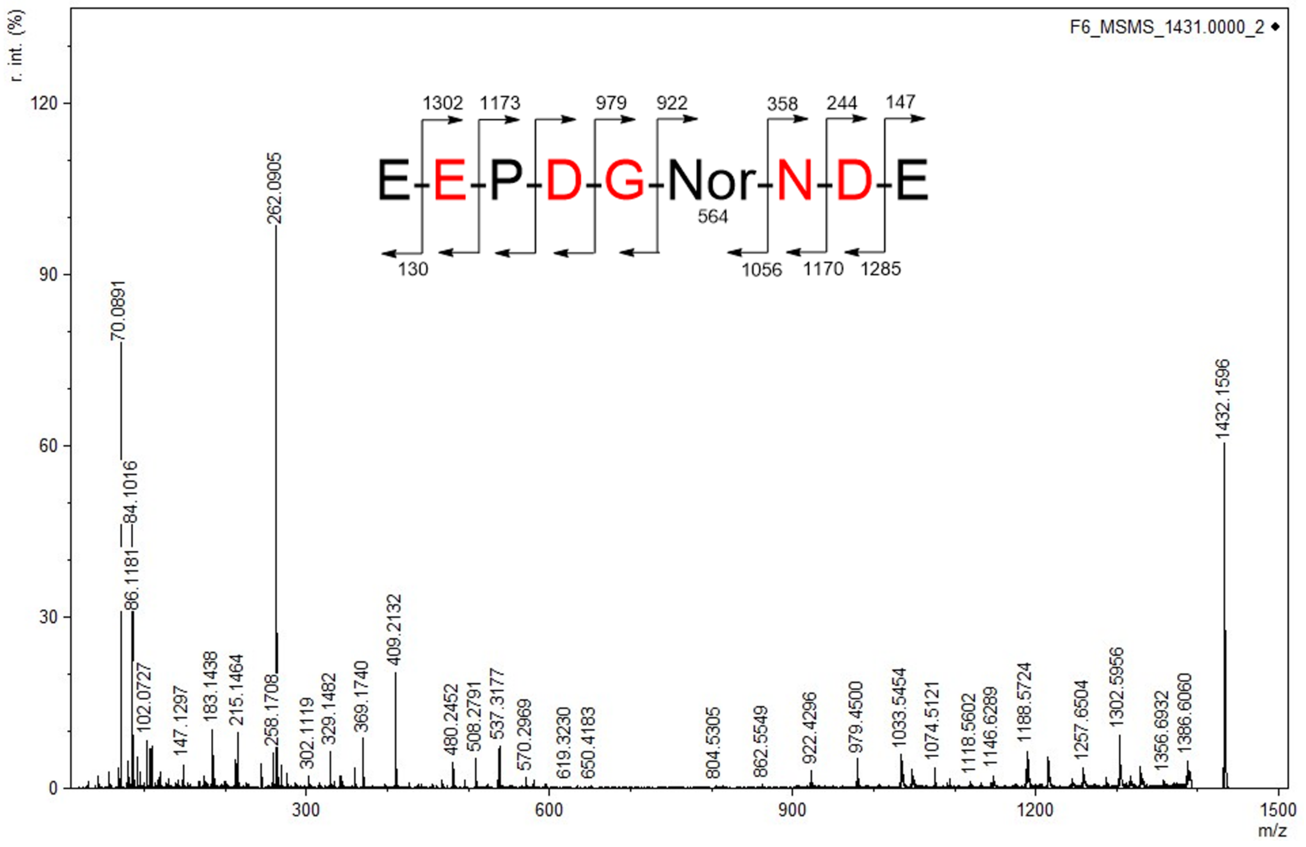

Figure S37. MS/MS of a random bead from the library, E-D-G-N-D. Parent ion: 1431.00.

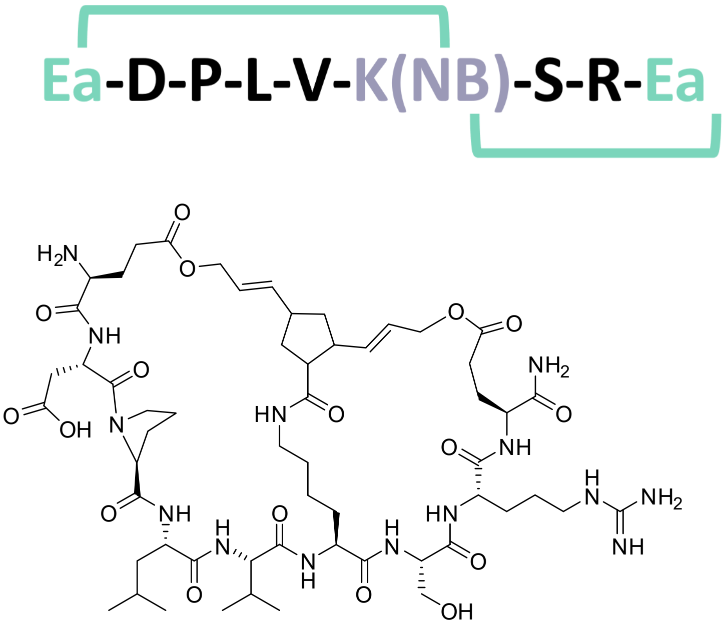

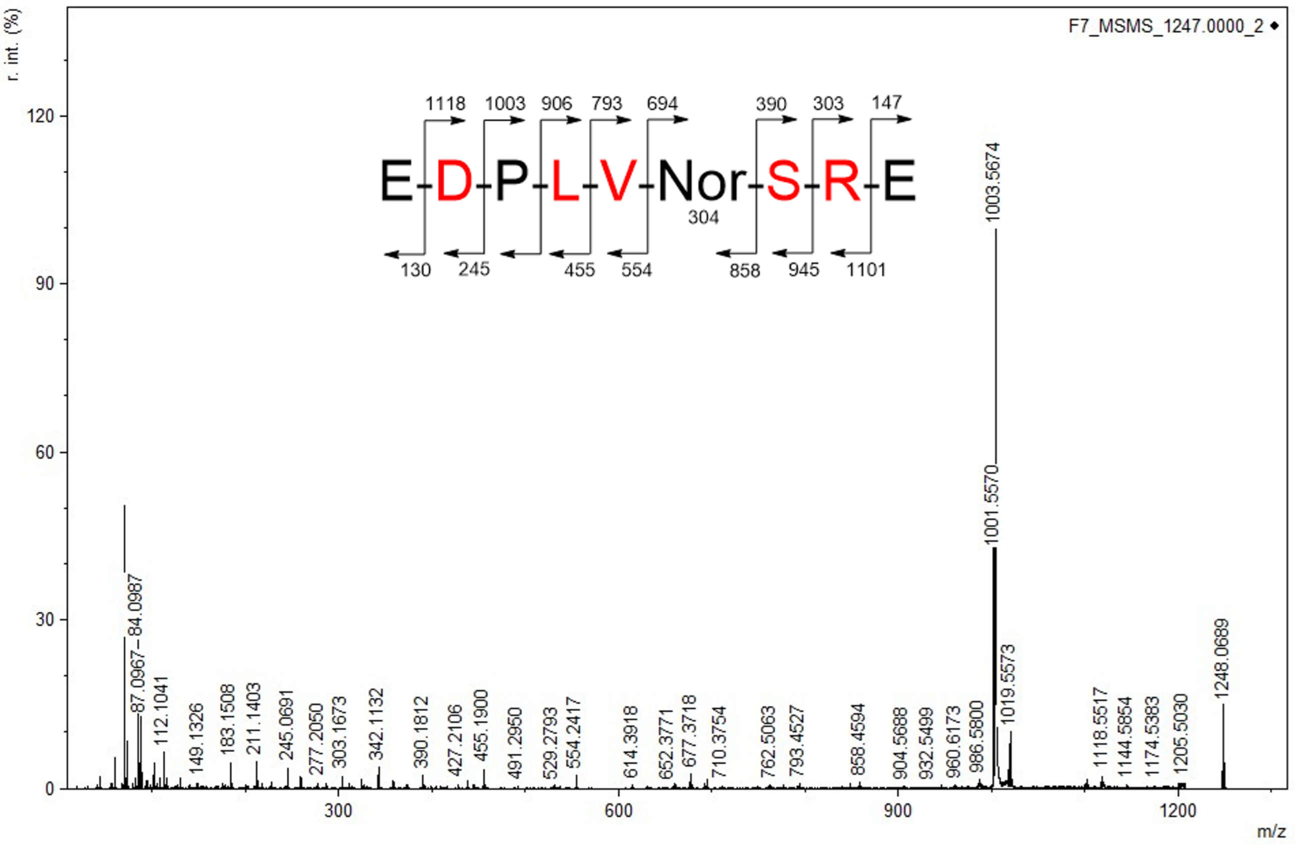

Figure S38. MS/MS of a random bead from the library, D-L-V-S-R. Parent ion: 1247.00.

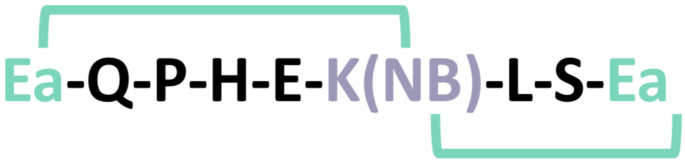

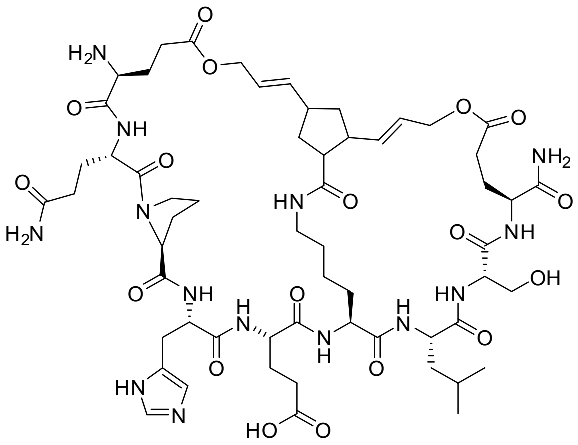

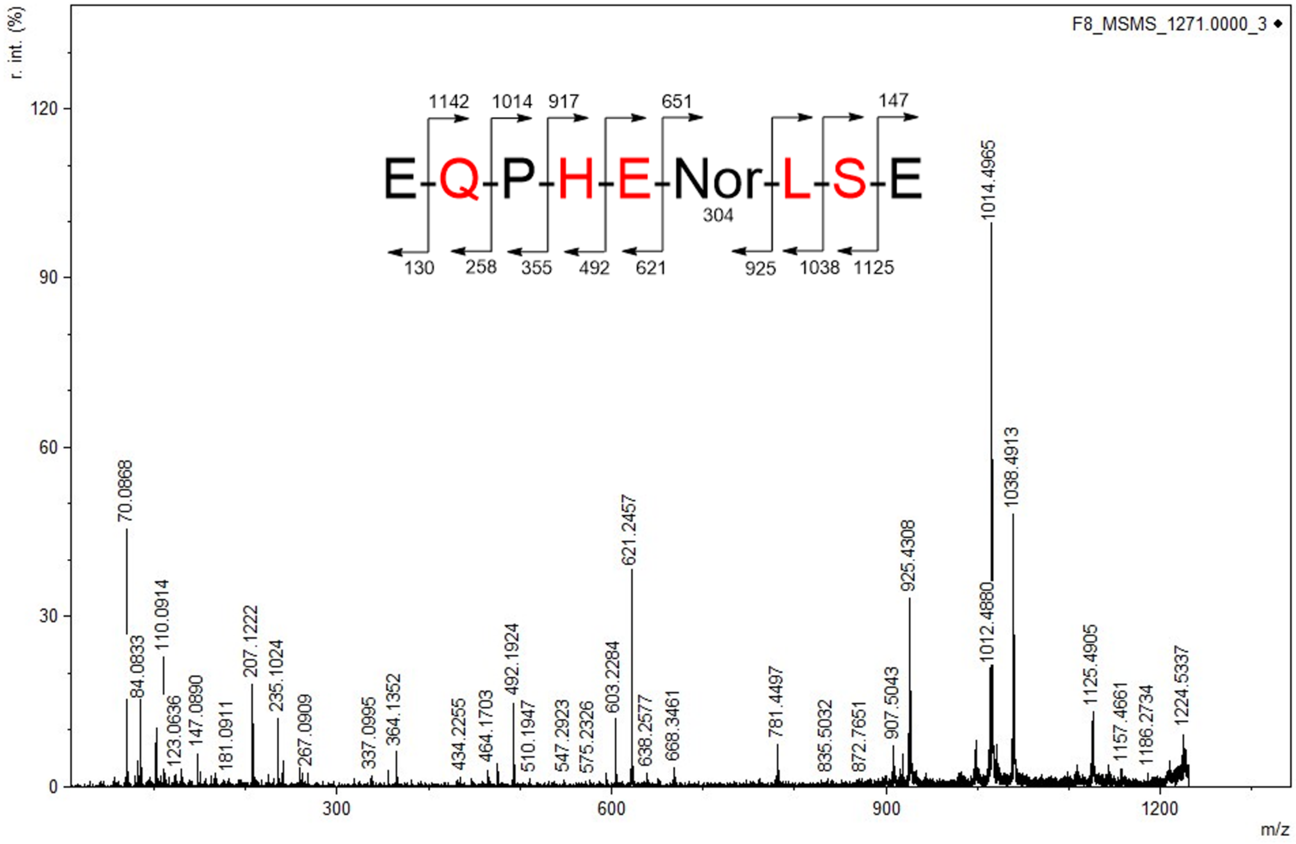

Figure S39. MS/MS of a random bead from the library, Q-H-E-L-S. Parent ion: 1271.00.

Ea-W-P-L-E-K(NB)-W-D-Ea

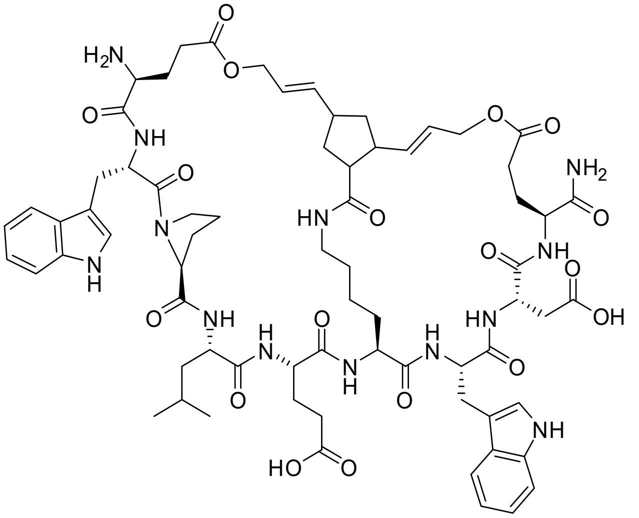

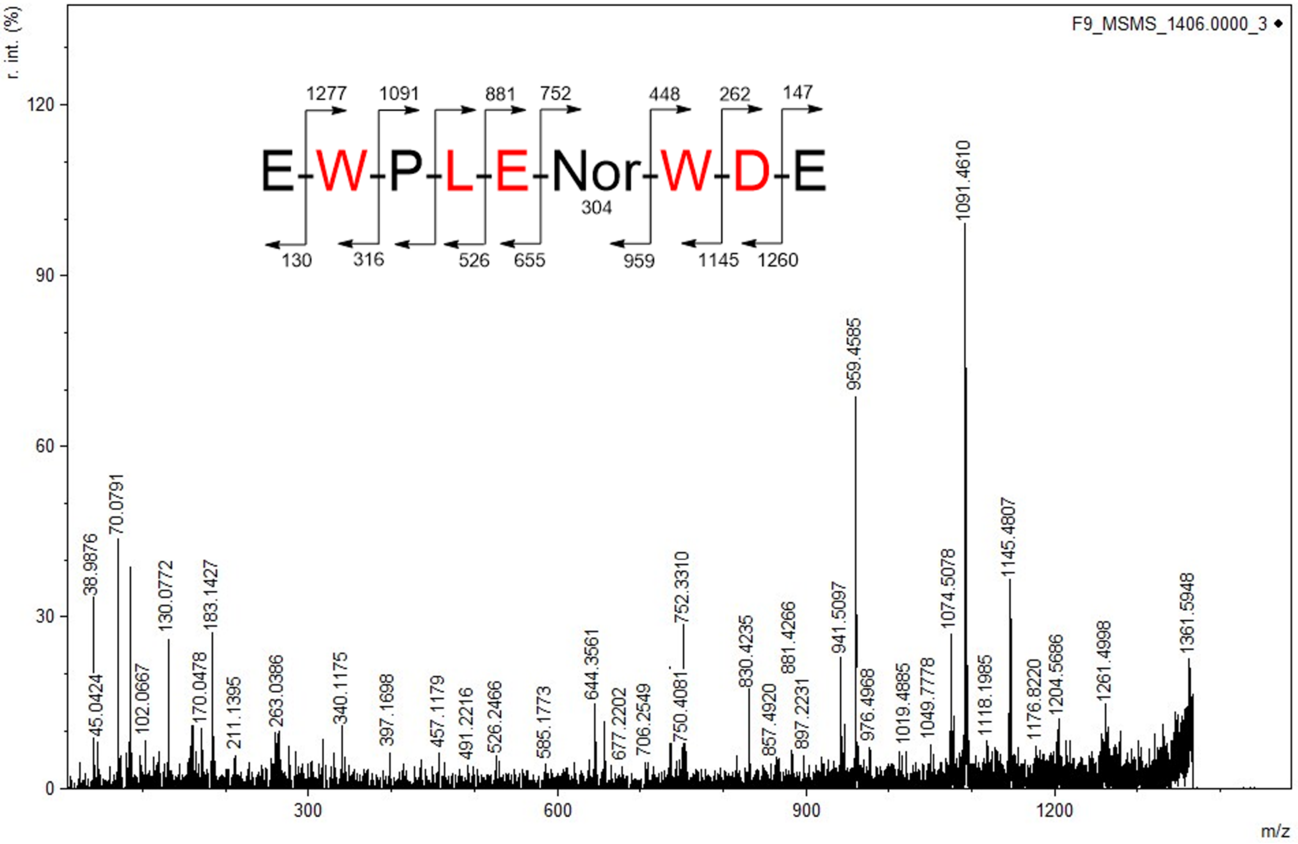

Figure S40. MS/MS of a random bead from the library, W-L-E-W-D. Parent ion: 1406.00.

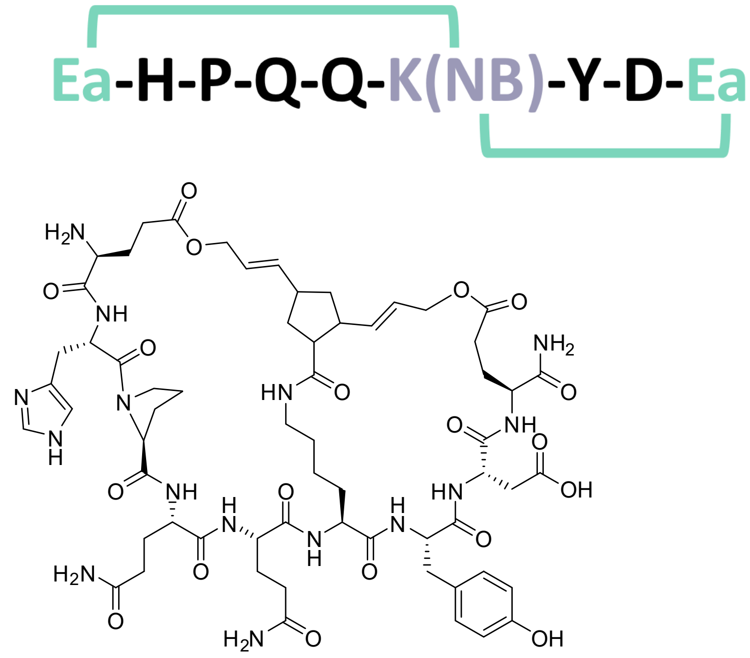

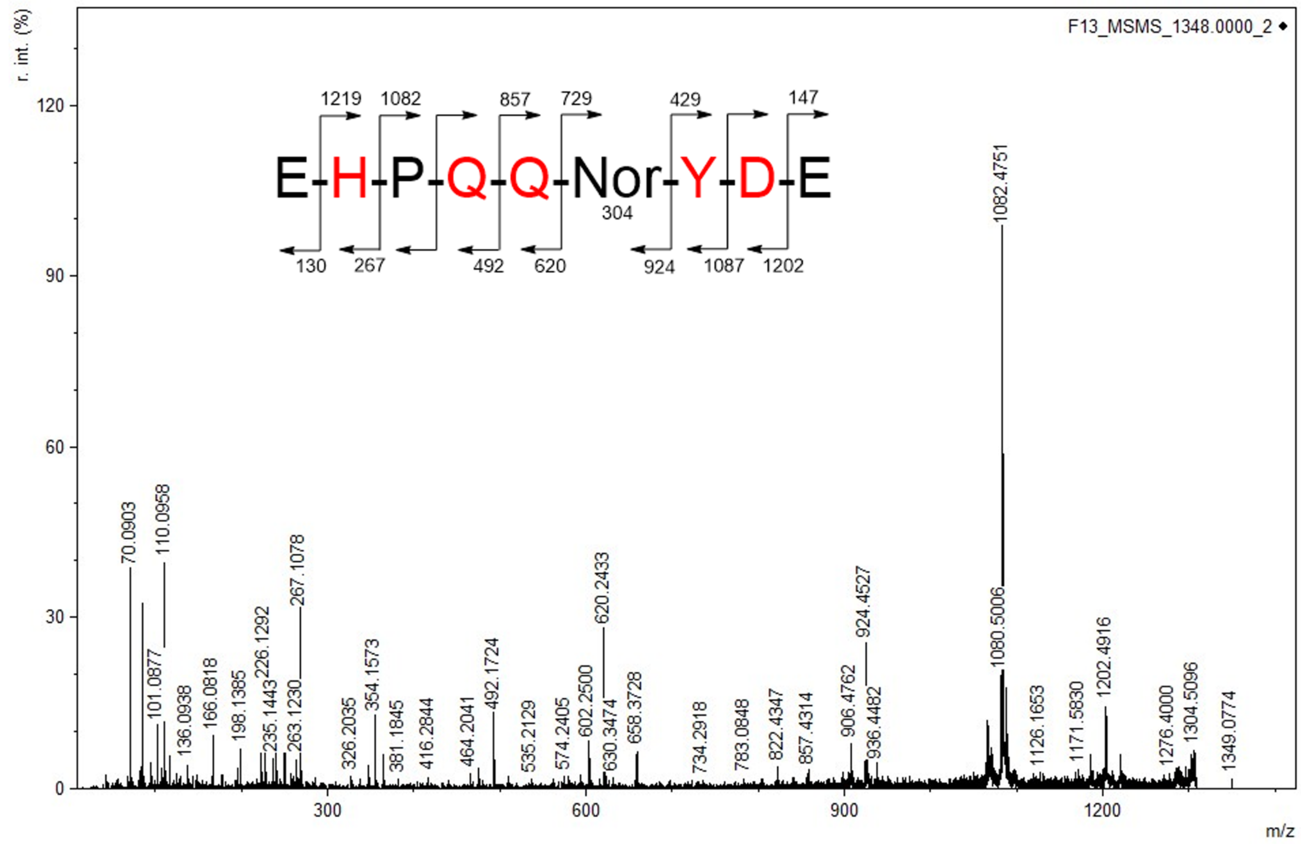

Figure S41. MS/MS of a random bead from the library, H-Q-Q-Y-D. Parent ion: 1348.00.

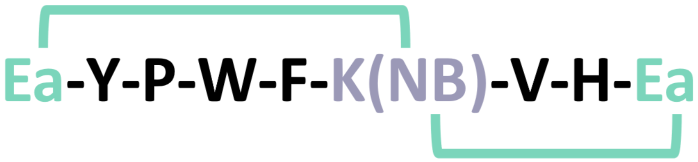

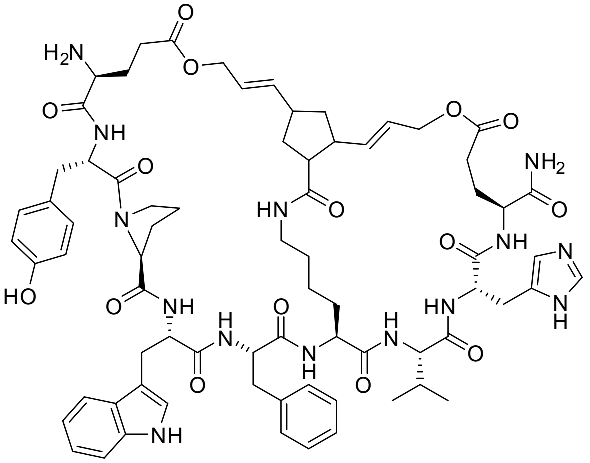

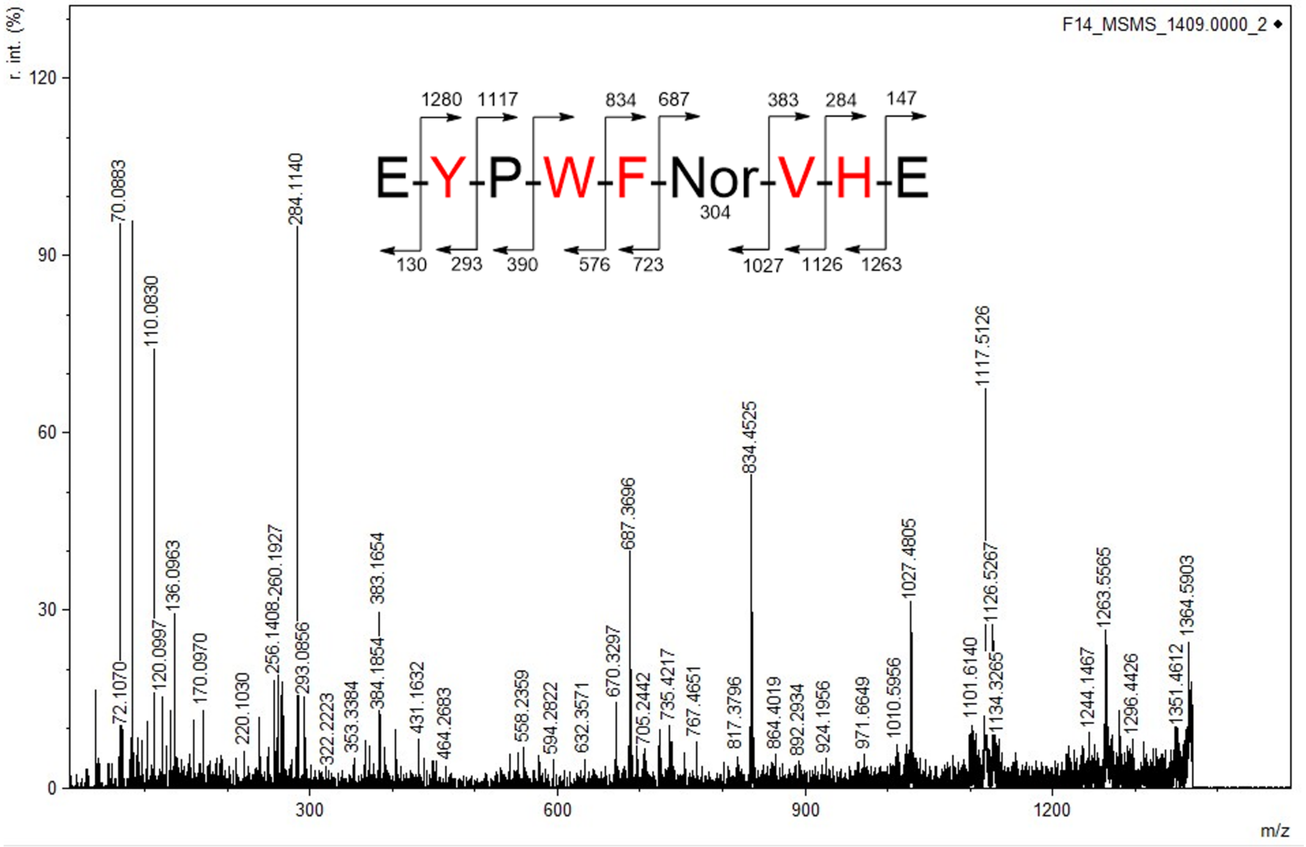

Figure S42. MS/MS of a random bead from the library, Y-W-F-V-H. Parent ion: 1409.00.

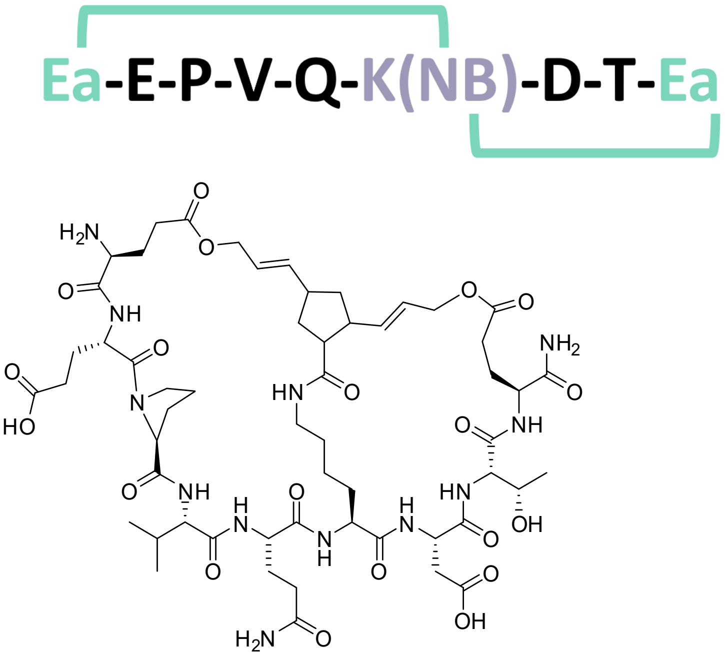

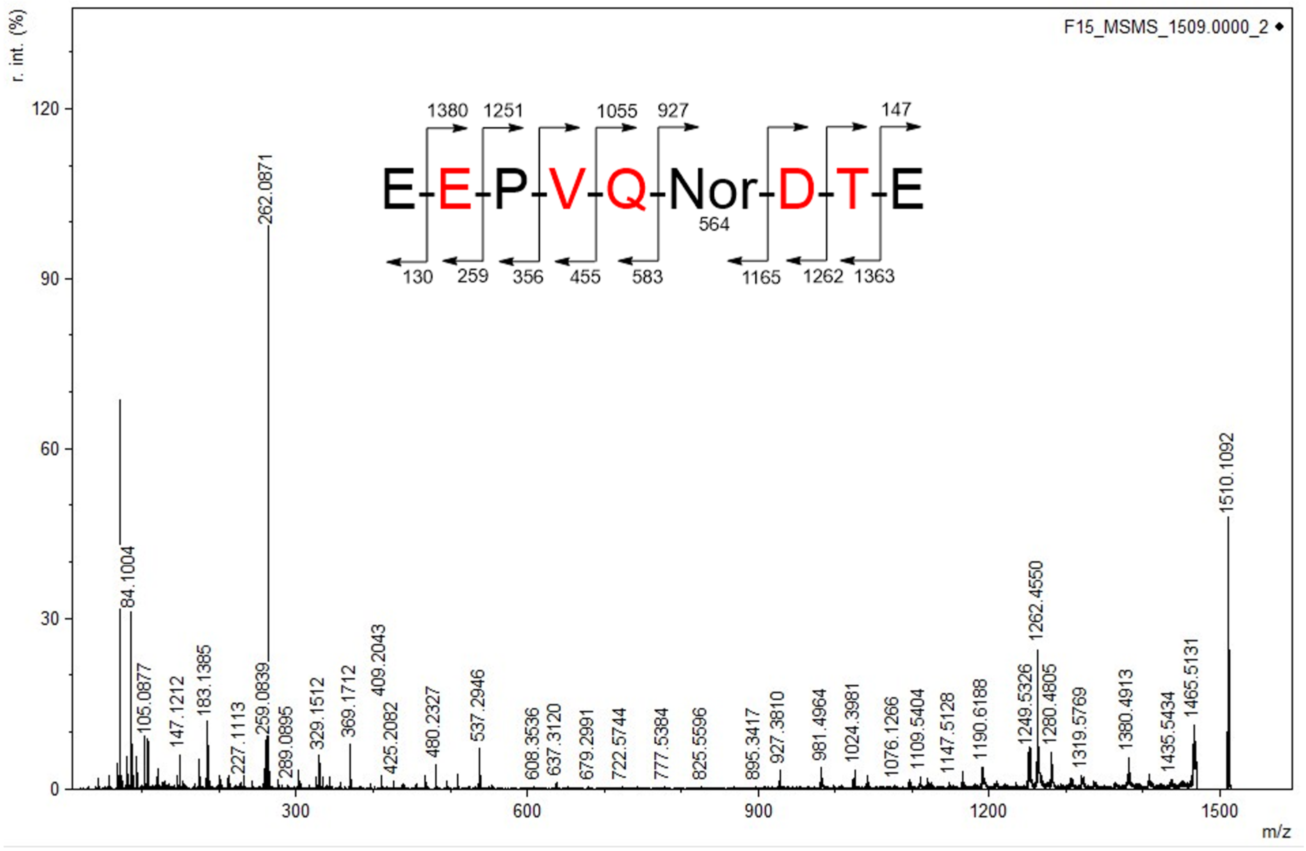

Figure S43. MS/MS of a random bead from the library, E-V-Q-D-T. Parent ion: 1509.00.

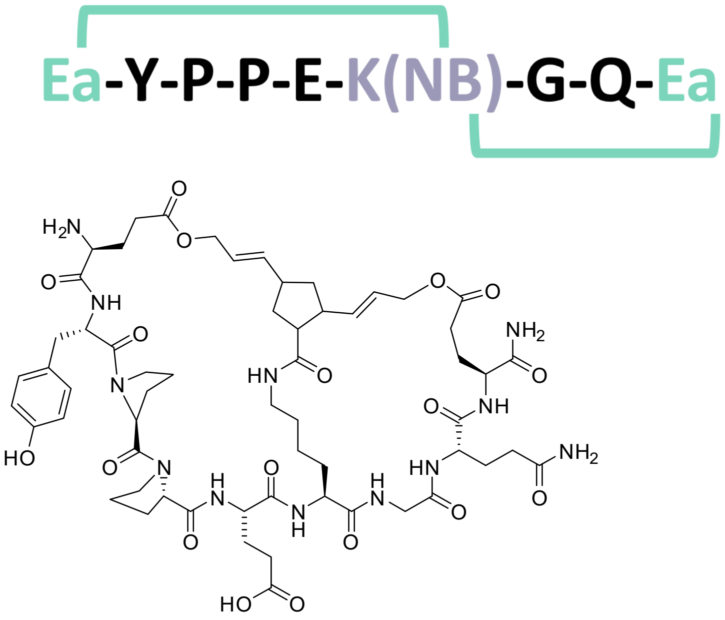

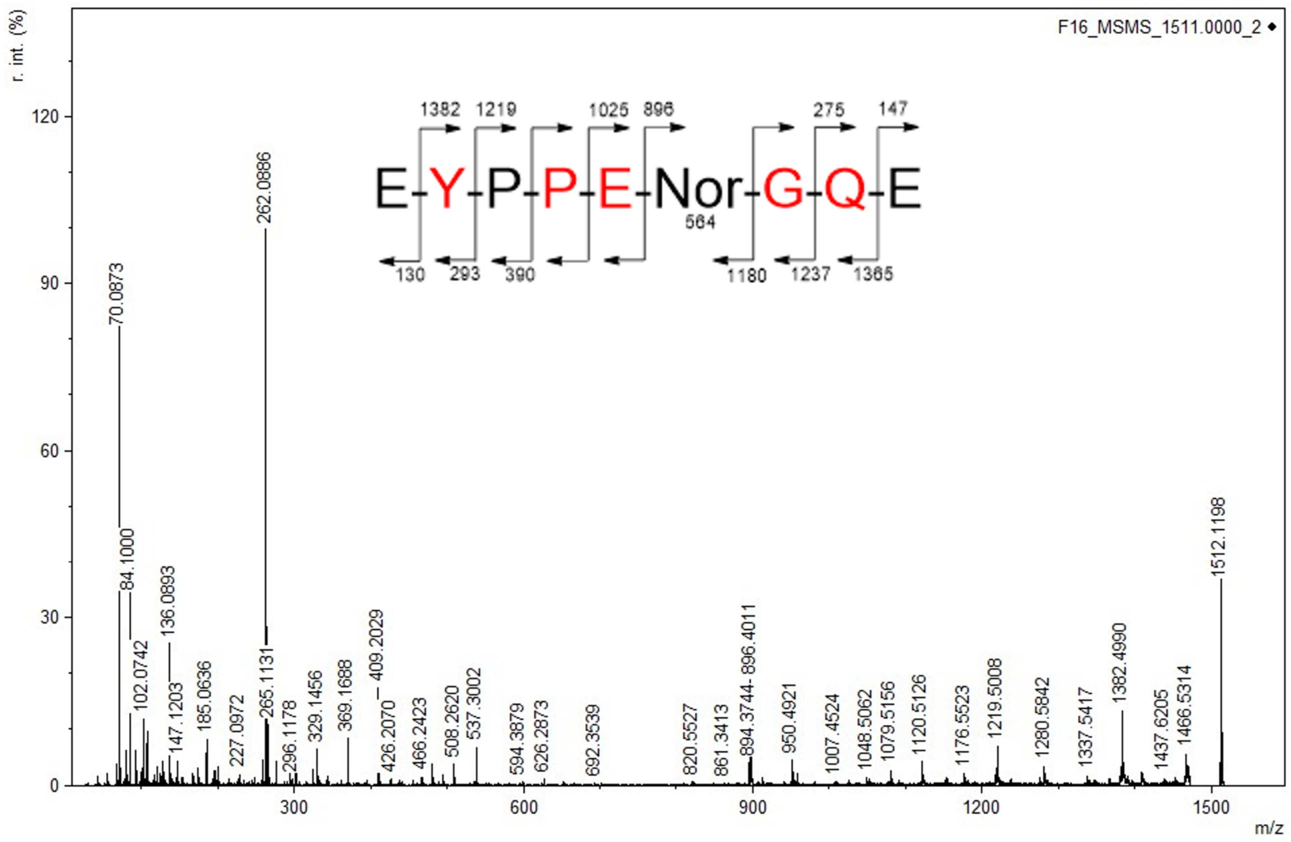

Figure S44. MS/MS of a random bead from the library, Y-P-E-G-Q. Parent ion: 1511.00.

Ea-N-P-E-F-K(NB)-H-D-Ea

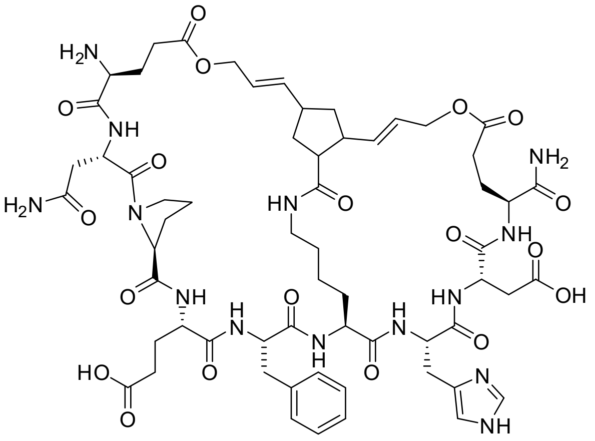

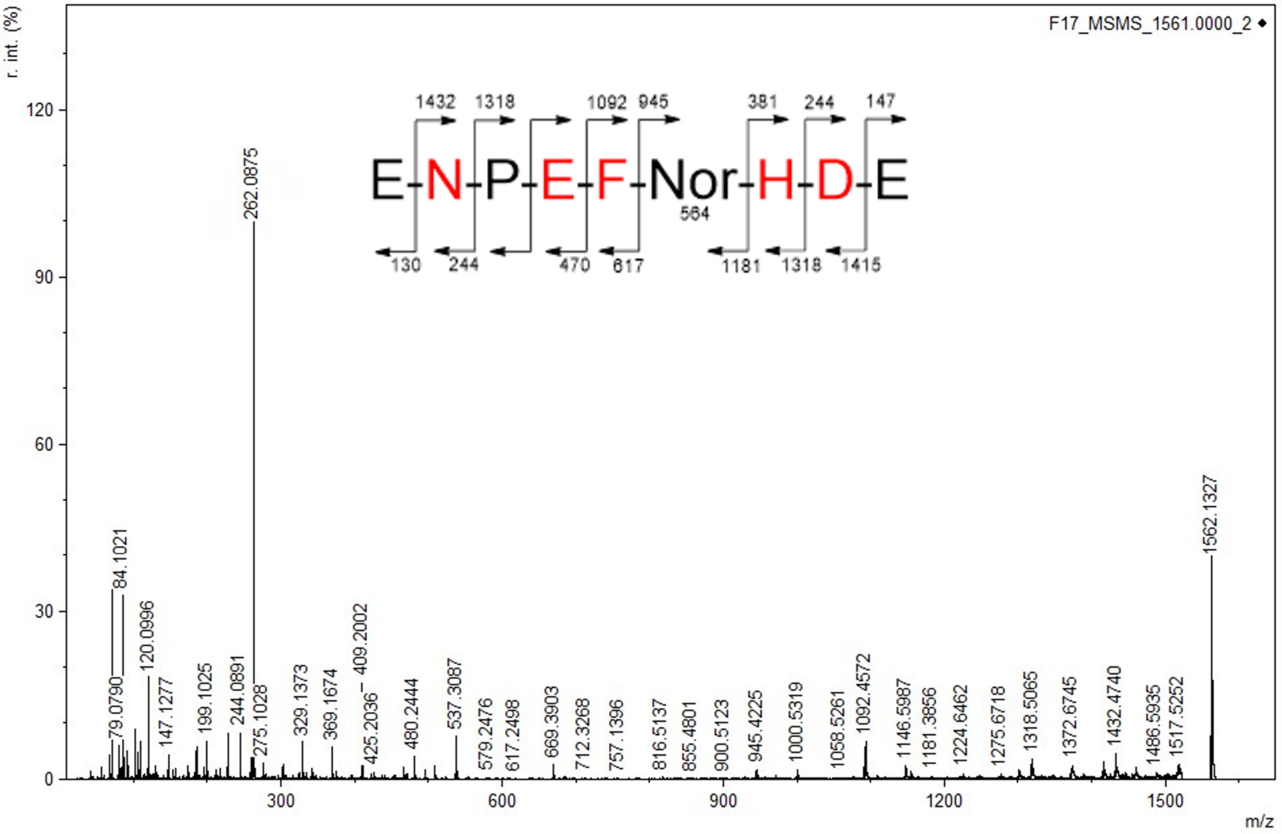

Figure S45. MS/MS of a random bead from the library, N-E-F-H-D. Parent ion: 1561.00.

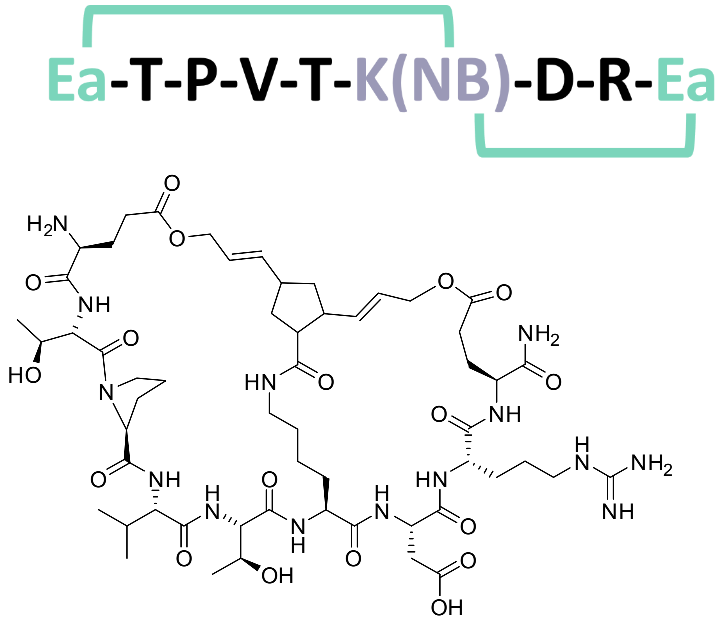

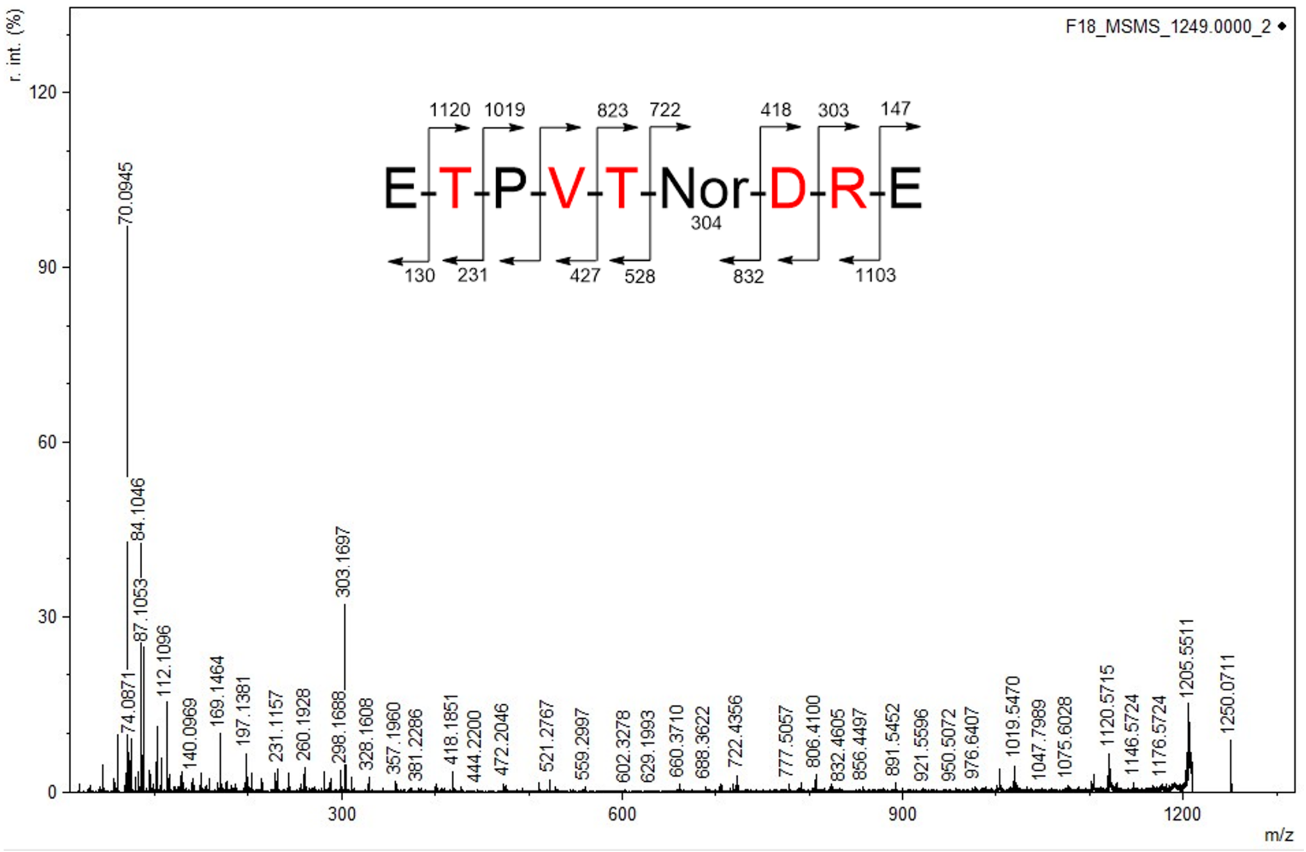

Figure S46. MS/MS of a random bead from the library, T-V-T-D-R. Parent ion: 1249.00.

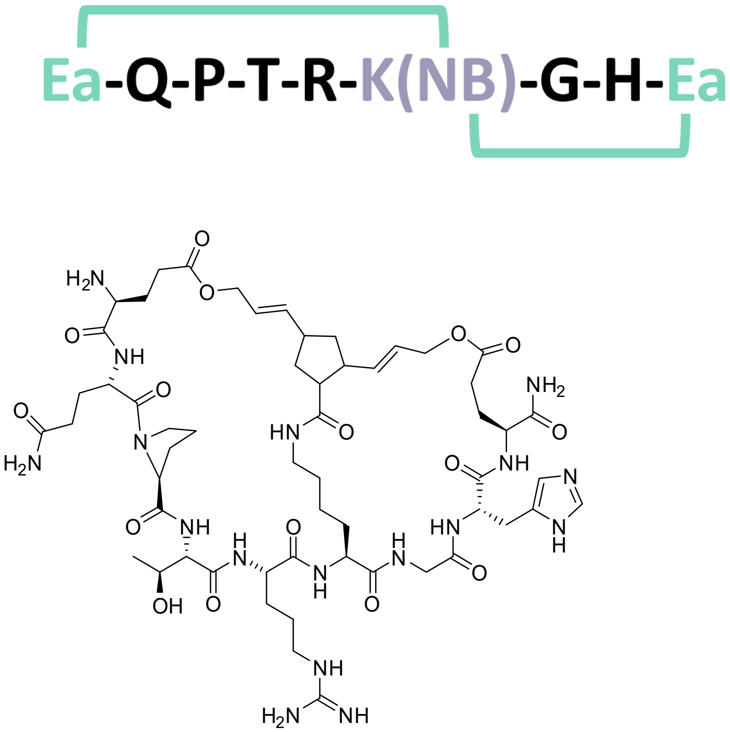

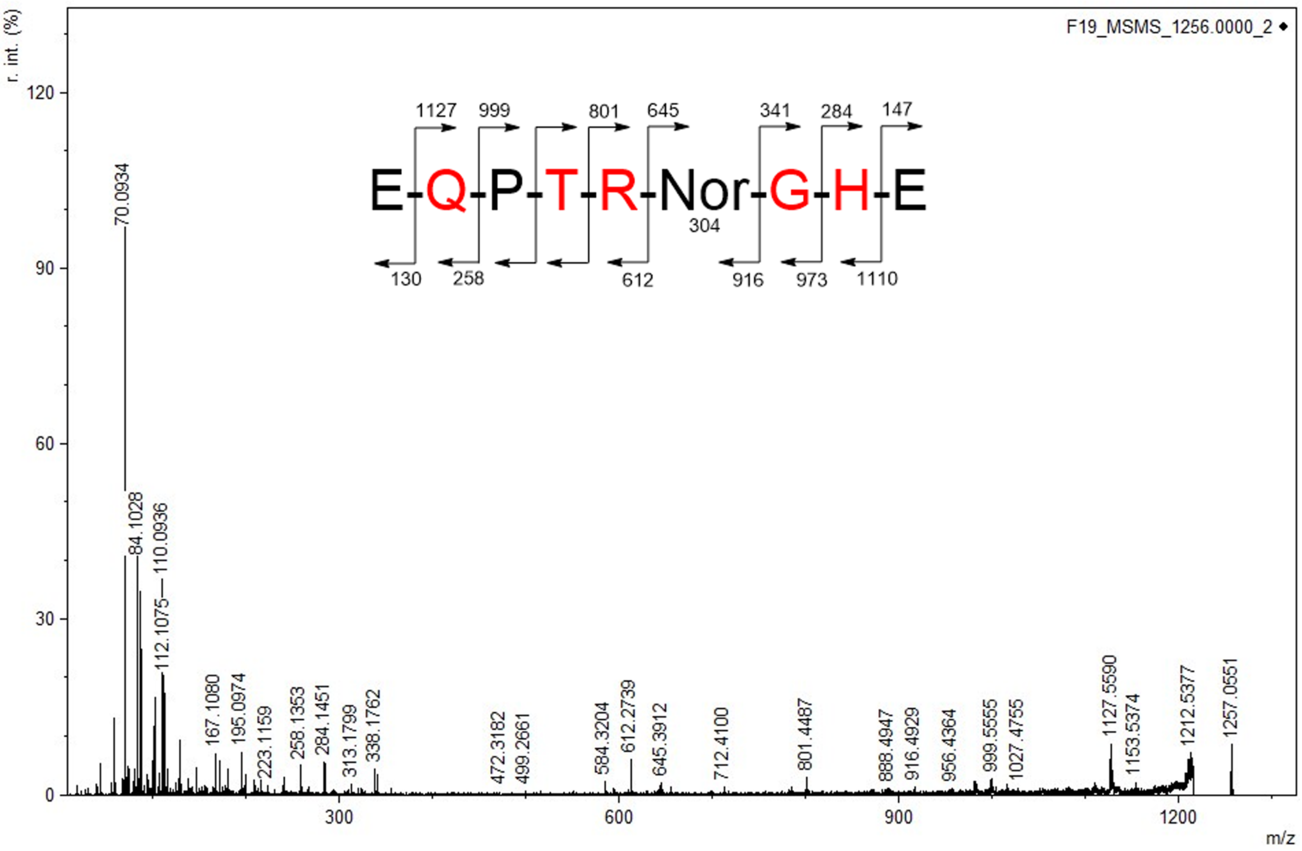

Figure S47. MS/MS of a random bead from the library, Q-T-R-G-H. Parent ion: 1256.00.

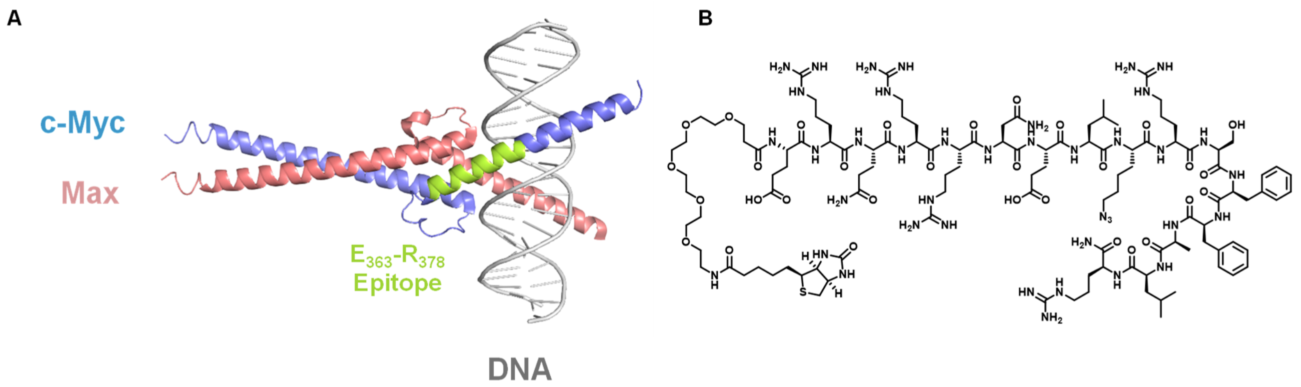

Figure S48. (A) The E_{363}-R_{378} epitope of the MYC (c-Myc) protein is involved in MYC-MAX binding and DNA recognition. Nevertheless, MYC itself exhibits disordered structures without the binding partners. Structure is based on PDB data (NDB: 5I50)[1]. (B) Structure[2] of Biotin-PEG5-epitope used for screening. The epitope sequence is Biotin-PEG5-ERQRRNEL-K(azide)-RSFFALR.

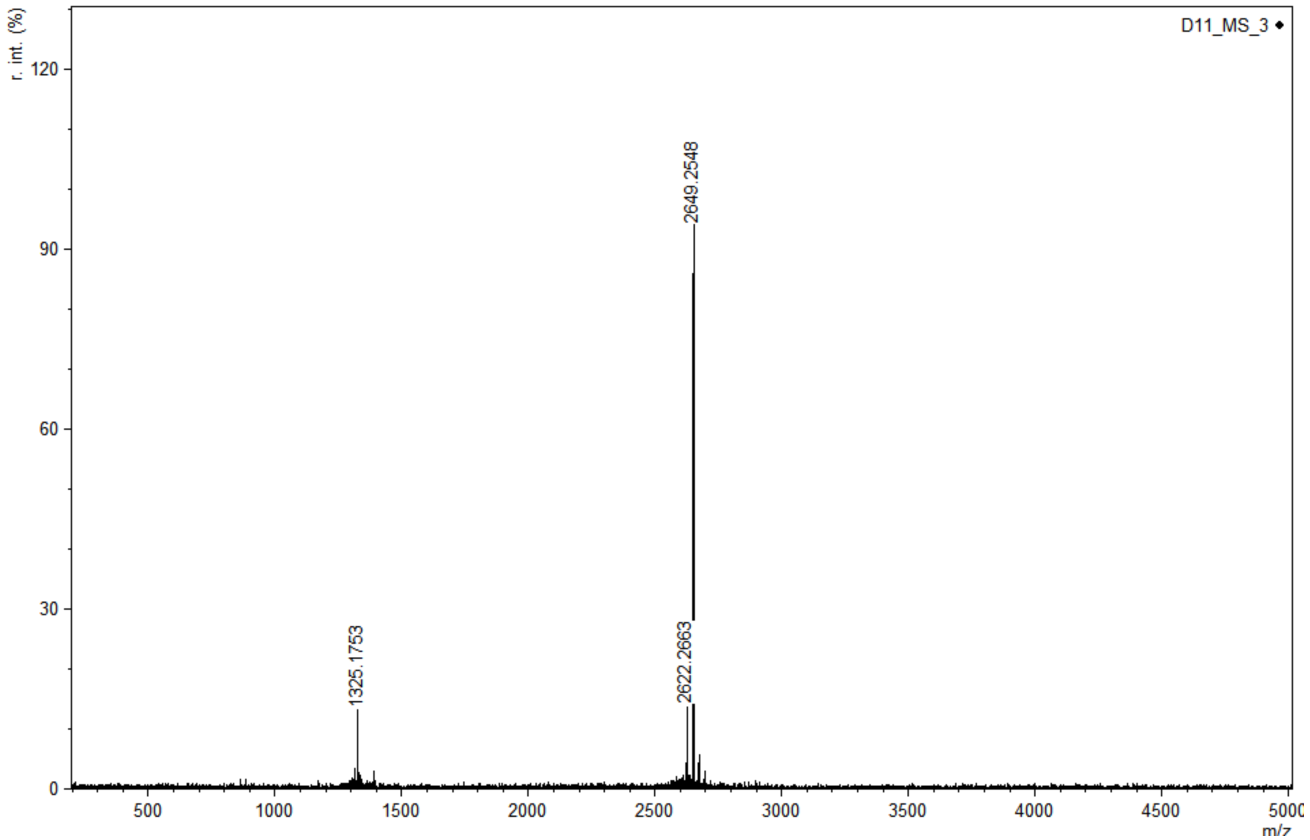

Figure S49. Mass spectrum for Biotin-PEG5-Epitope (azido lysine) (MALDI-TOF). $[M+H]^+$ calcd 2648.42, found 2649.25; $[M+2H]^{2+}$ 1325.21, found 1325.18.

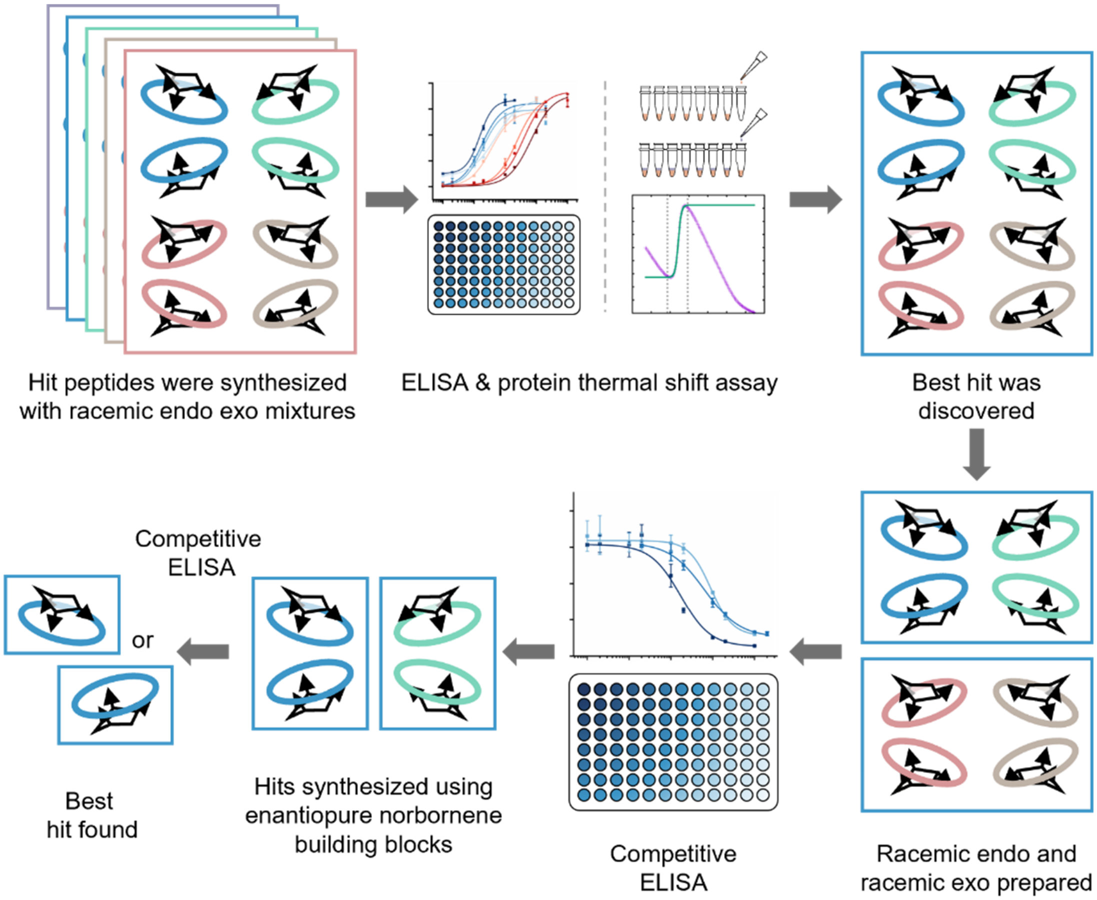

Figure S50. Workflow for candidate discovery.

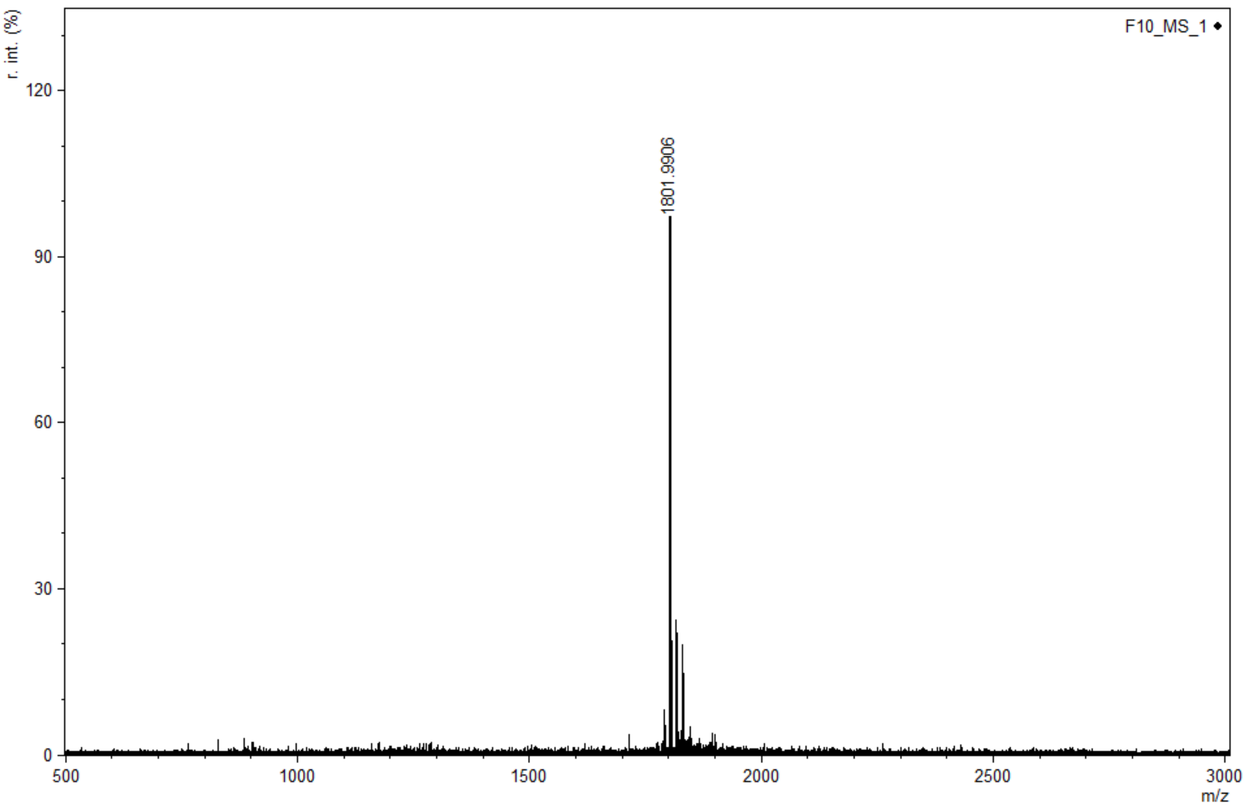

Figure S51. Mass spectrum of Biotin-PEG5-NT-B1 (MALDI-TOF). $[M+H]^+$ calcd 1801.93, found 1801.99.

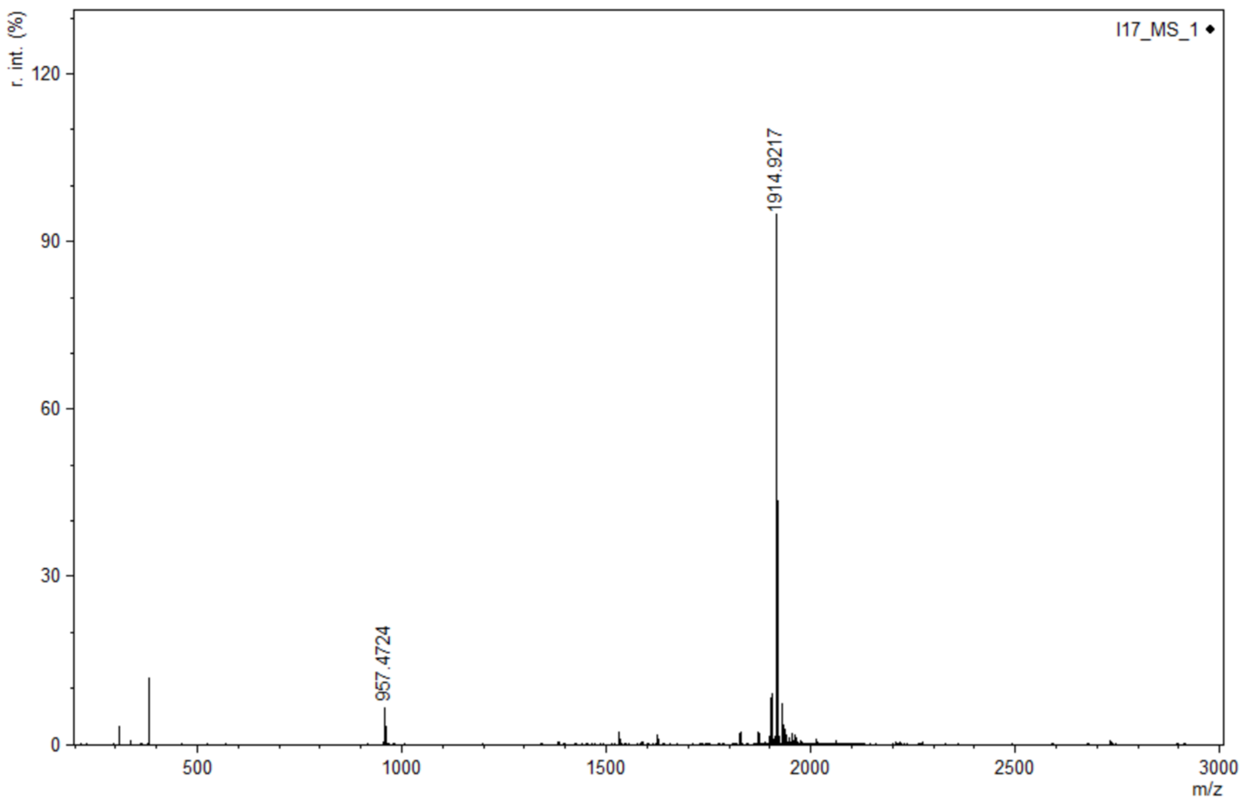

Figure S52. Mass spectrum of Biotin-PEG5-NT-B8 (MALDI-TOF). $[M+H]^+$ calcd 1913.99, found 1914.92.

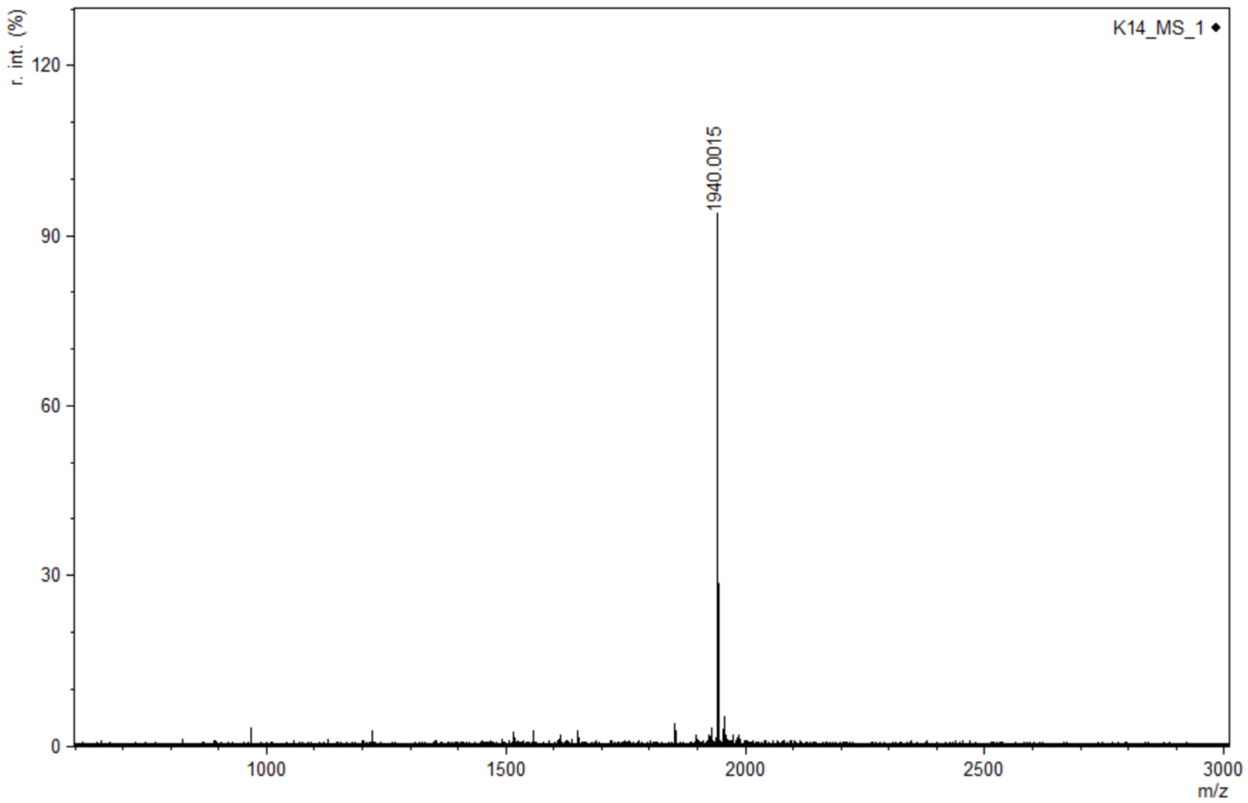

Figure S53. Mass spectrum of Biotin-PEG5-NT-B2 (MALDI-TOF). $[M+H]^+$ calcd 1939.02, found 1940.00.

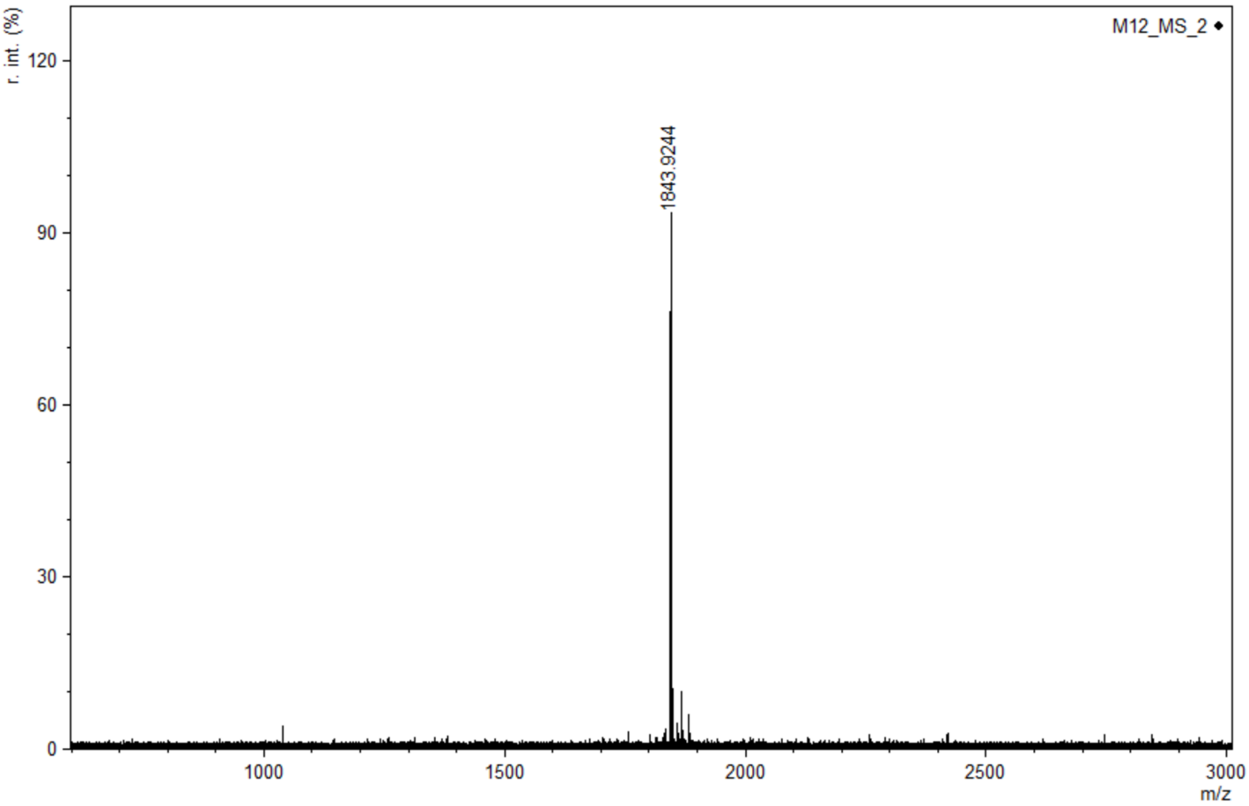

Figure S54. Mass spectrum of Biotin-PEG5-NT-B4 (MALDI-TOF). $[M+H]^+$ calcd 1842.96, found 1843.92.

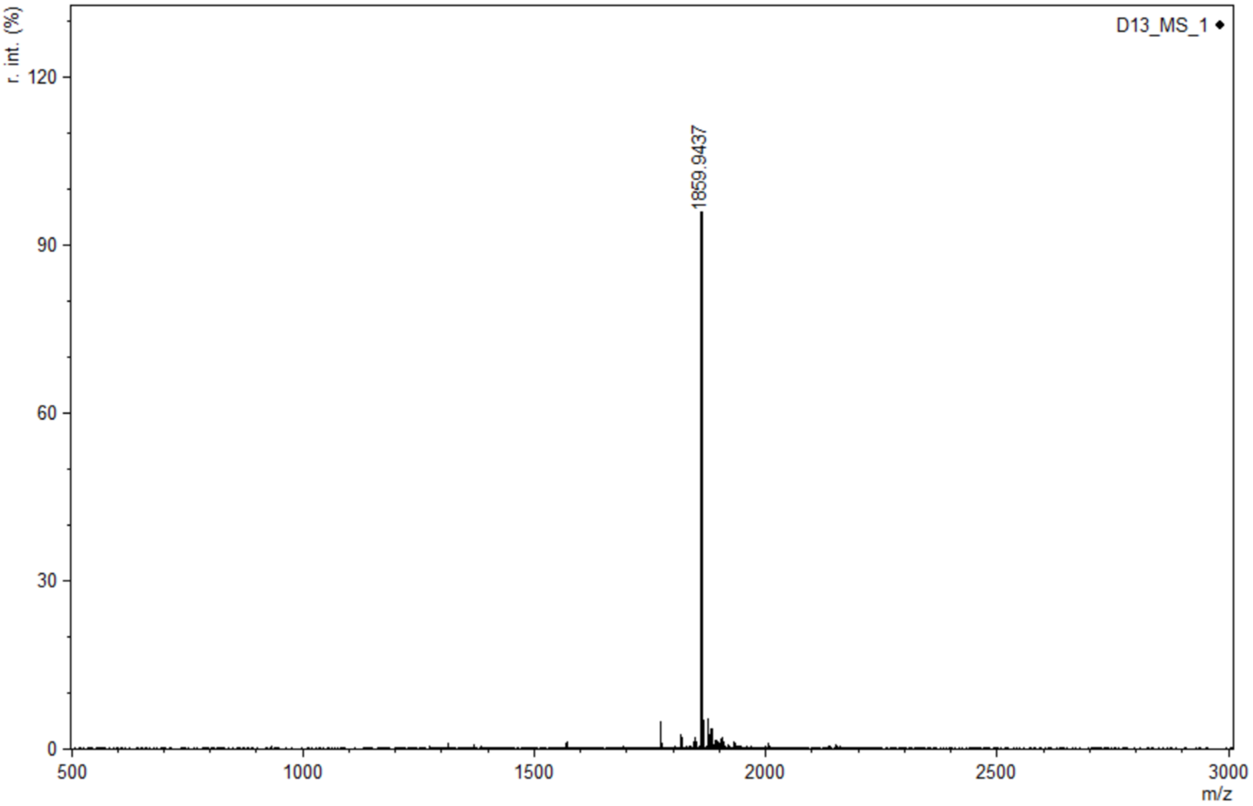

Figure S55. Mass spectrum of Biotin-PEG5-NT-B3 (MALDI-TOF). $[M+H]^+$ calcd 1858.98, found 1859.94.

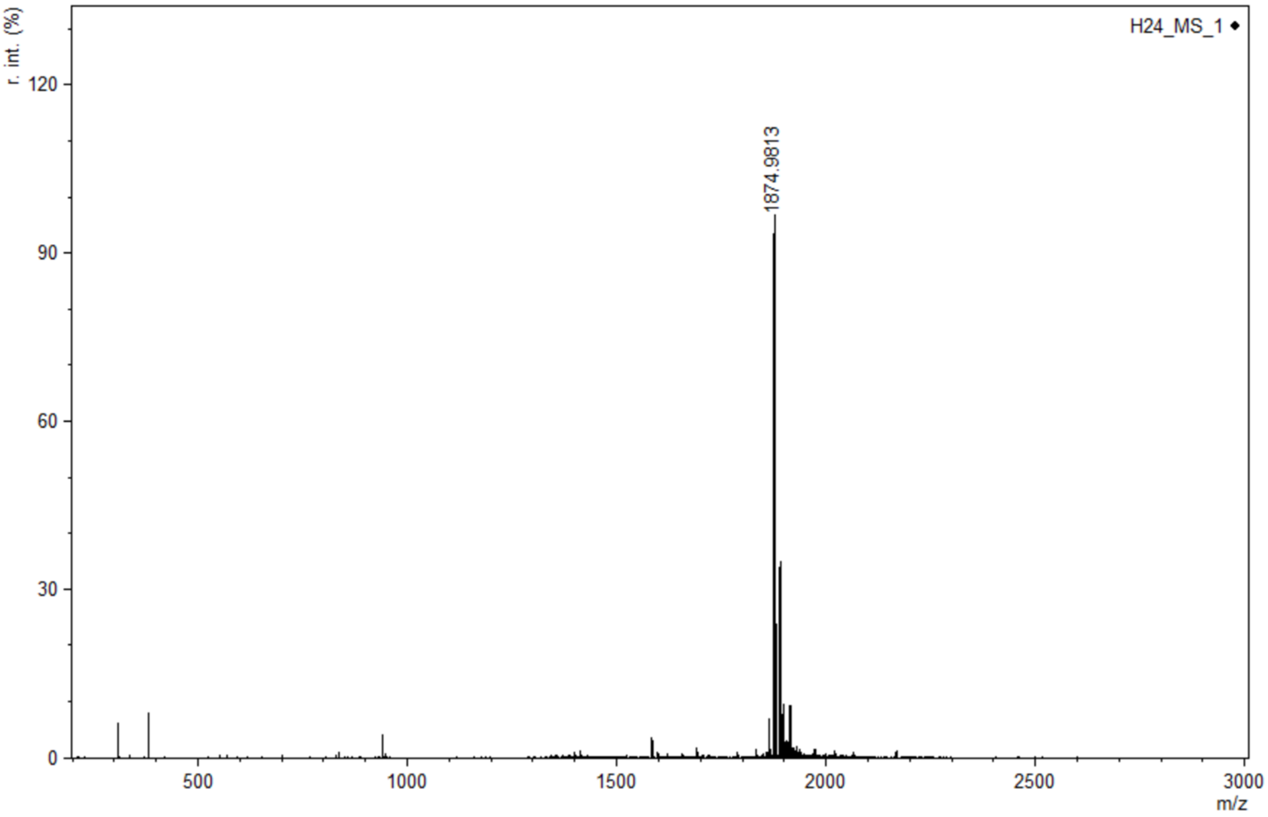

Figure S56. Mass spectrum of Biotin-PEG5-NT-B5 (MALDI-TOF). $[M+H]^+$ calcd 1873.98, found 1874.94.

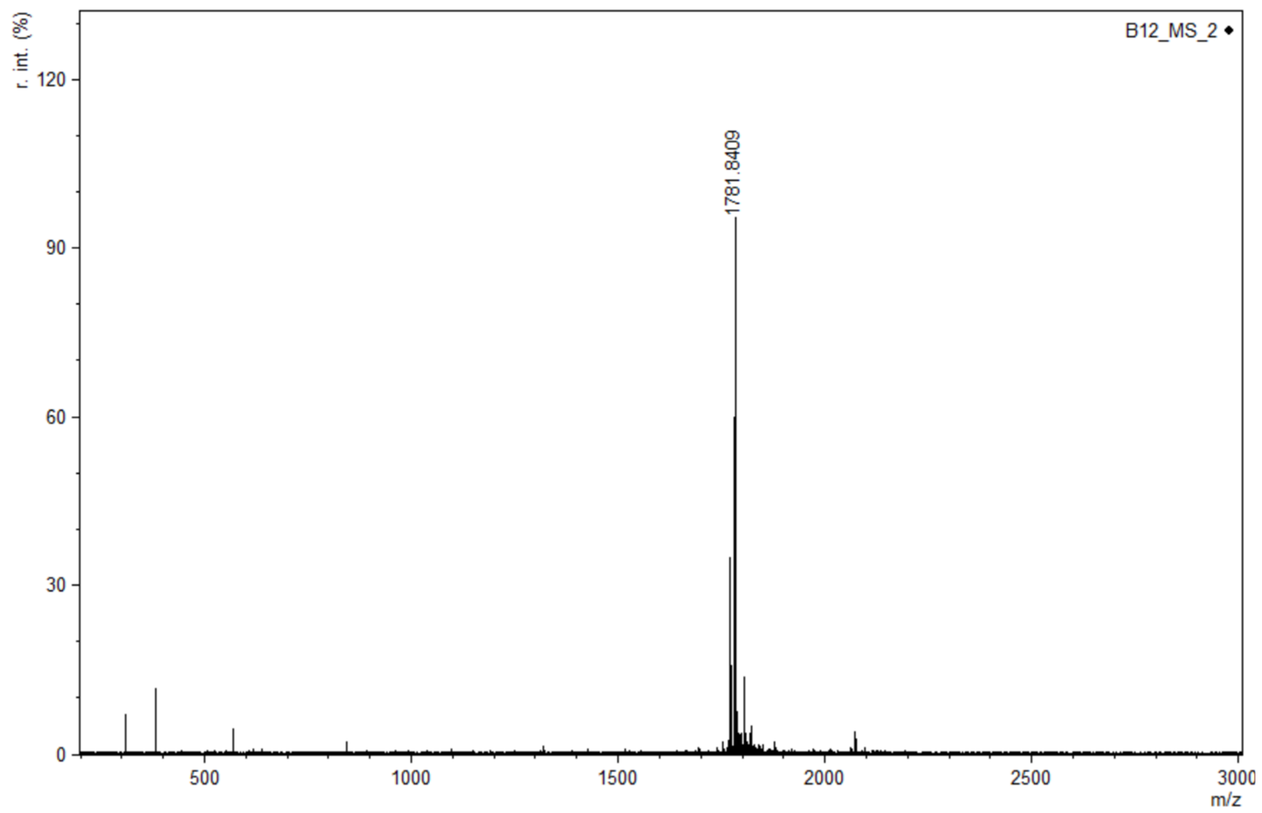

Figure S57. Mass spectrum of Biotin-PEG5-NT-B6 (MALDI-TOF). $[M+H]^+$ calcd 1780.95, found 1781.84.

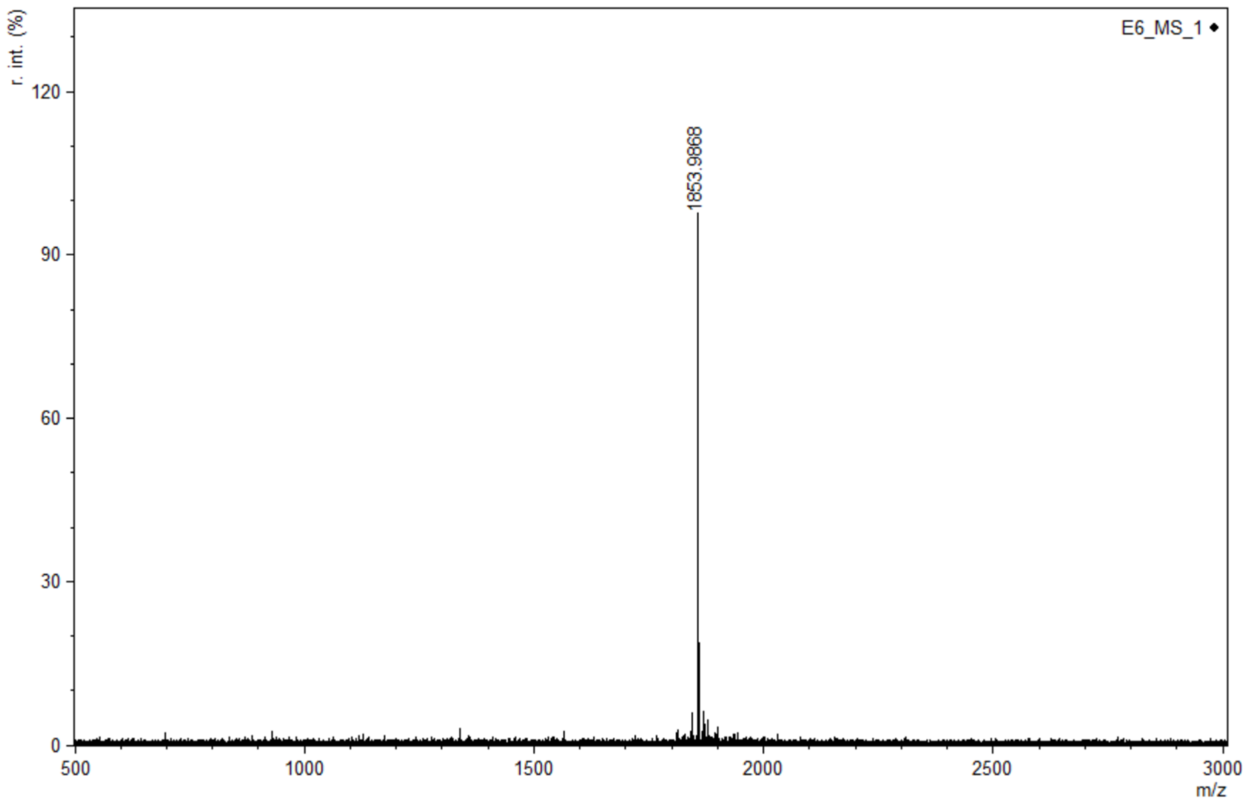

Figure S58. Mass spectrum of Biotin-PEG5-NT-B7 (MALDI-TOF). $[M+H]^+$ calcd 1853.84, found 1853.99.

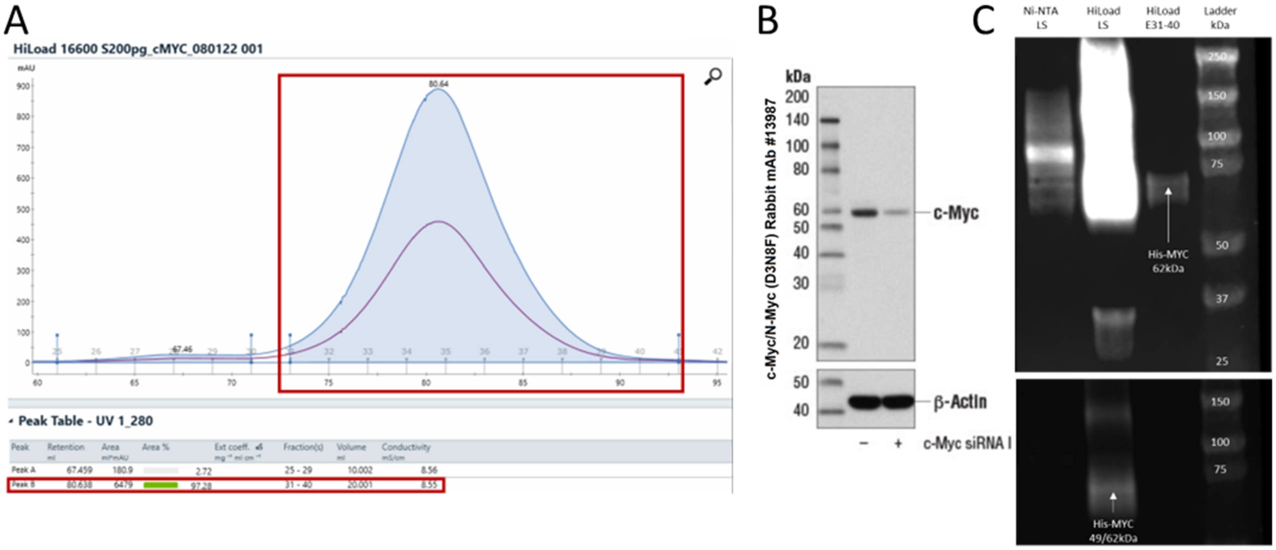

Figure S59. Purification and quality control of recombinant MYC protein expression. (**A**) SEC chromatogram demonstrating the purity of the recombinant MYC. (**B**) Antibody QC results obtained from the vendor webpage (https://www.cellsignal.com/products/primary-antibodies/c-myc-n-myc-d3n8f-rabbit-mab/13987). (**C**) Western blot results showing the successful expression of His-tagged MYC recombinant protein.

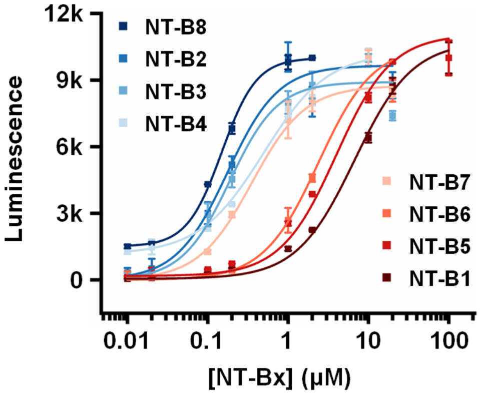

Hit	EC_{50} (μM)	Tm shift (°C)
NT-B1	6.710	1.892
NT-B2	0.174	2.206
NT-B3	0.187	1.188
NT-B4	0.425	1.495
NT-B5	3.960	1.840
NT-B6	2.384	1.852
NT-B7	0.353	-0.103
NT-B8	0.151	1.340

Figure S60. ELISA results showing the binding between Biotin-PEG5-NT-B1~8 (rac endo and rac exo mixtures) with recombinant MYC protein. EC_{50} were listed in the table (left). The binding EC_{50} values were consistent with melting temperature shifts in protein thermal shift assays obtained using unlabeled peptides (right).

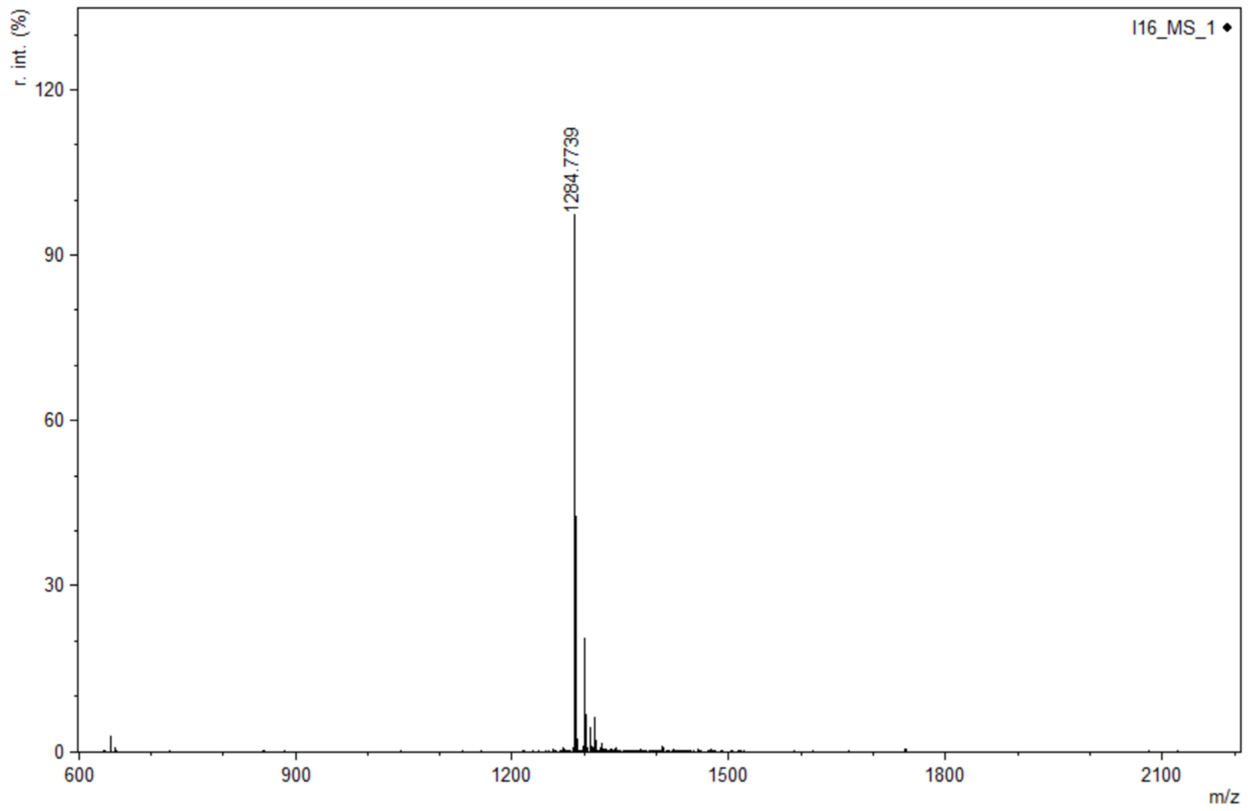

Figure S61. Mass spectrum of NT-B1 (MALDI-TOF). $[M+H]^+$ calcd 1284.69, found 1284.77.

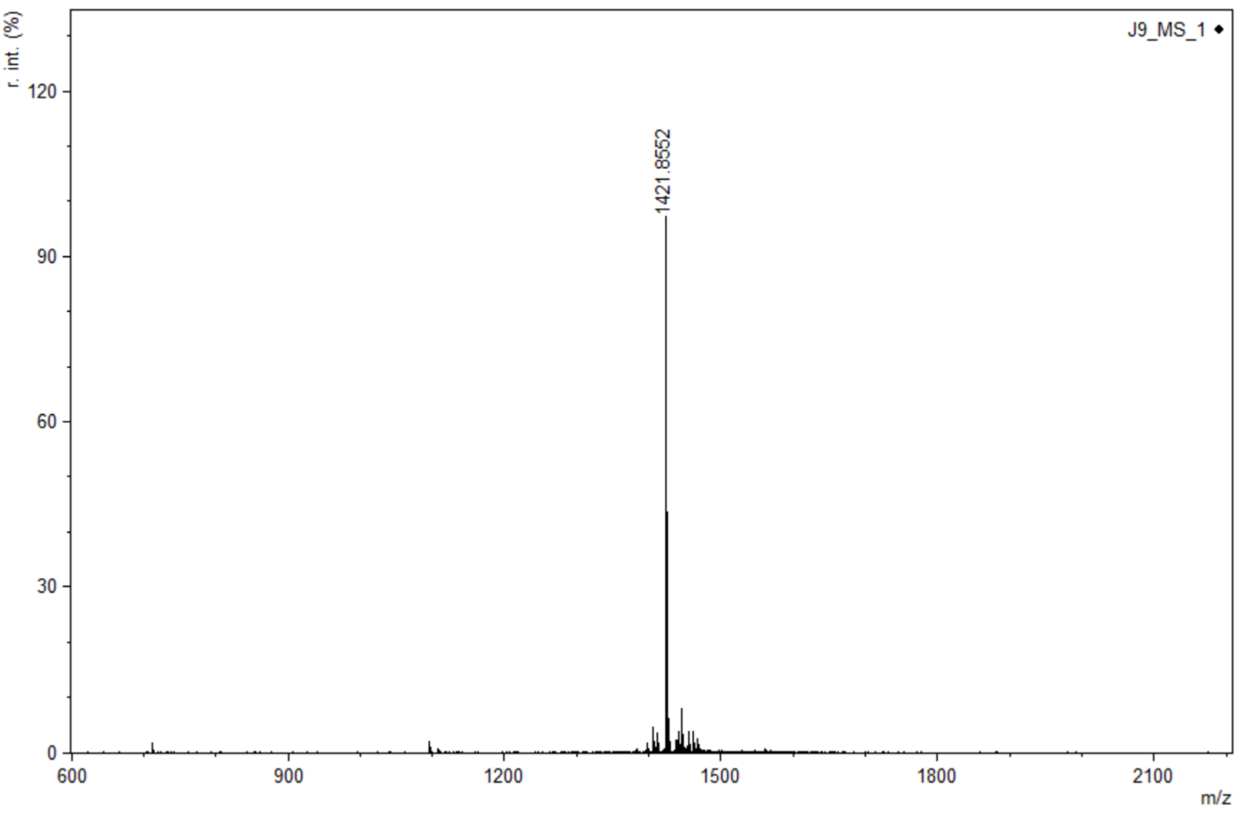

Figure S62. Mass spectrum of NT-B2 (MALDI-TOF). $[M+H]^+$ calcd 1421.77, found 1421.86.

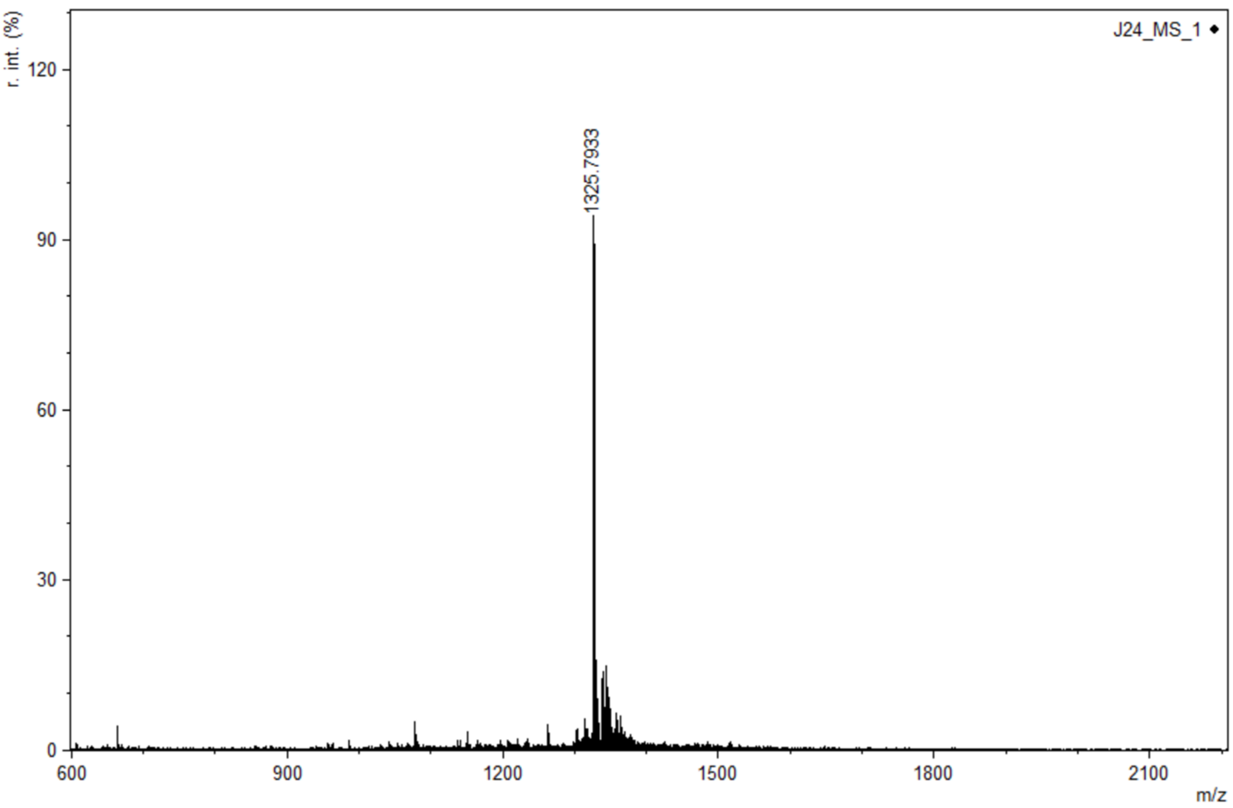

Figure S63. Mass spectrum of NT-B4 (MALDI-TOF). $[M+H]^+$ calcd 1325.72, found 1325.79.

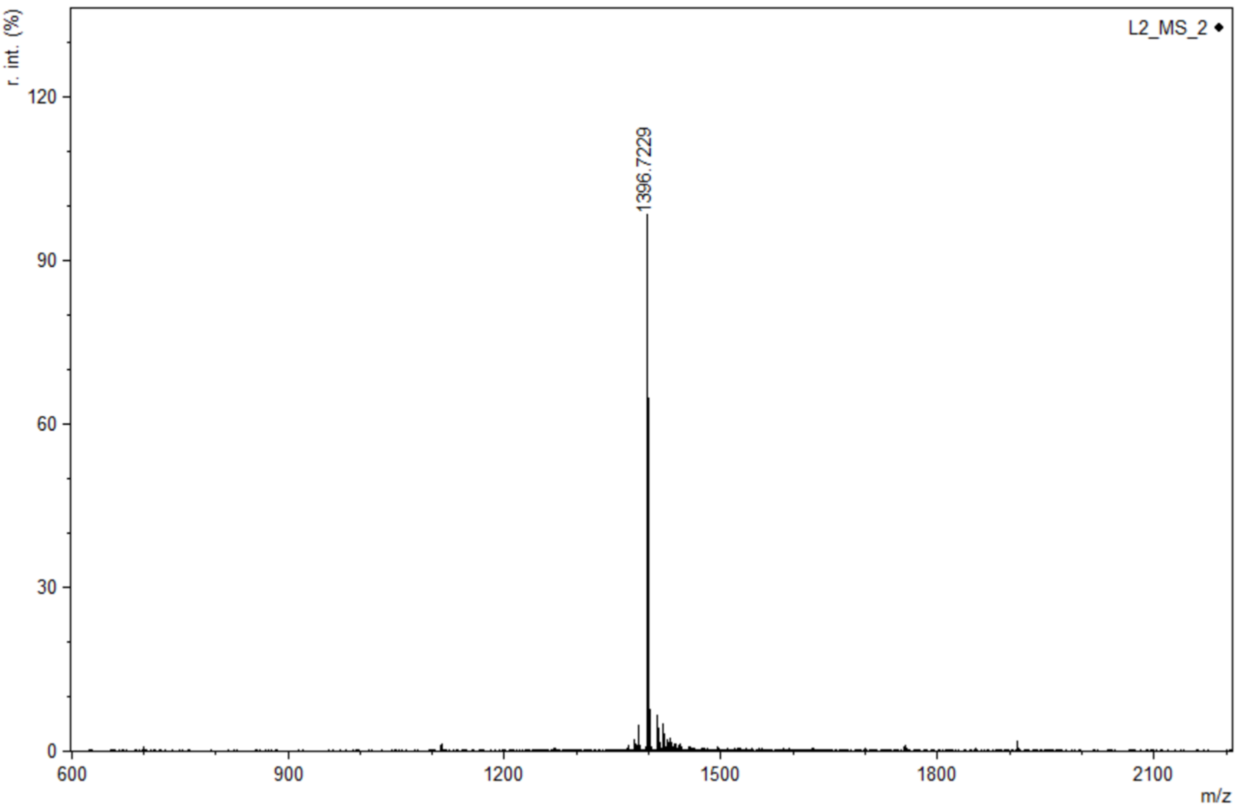

Figure S64. Mass spectrum of NT-B8 (MALDI-TOF). $[M+H]^+$ calcd 1396.74, found 1396.72.

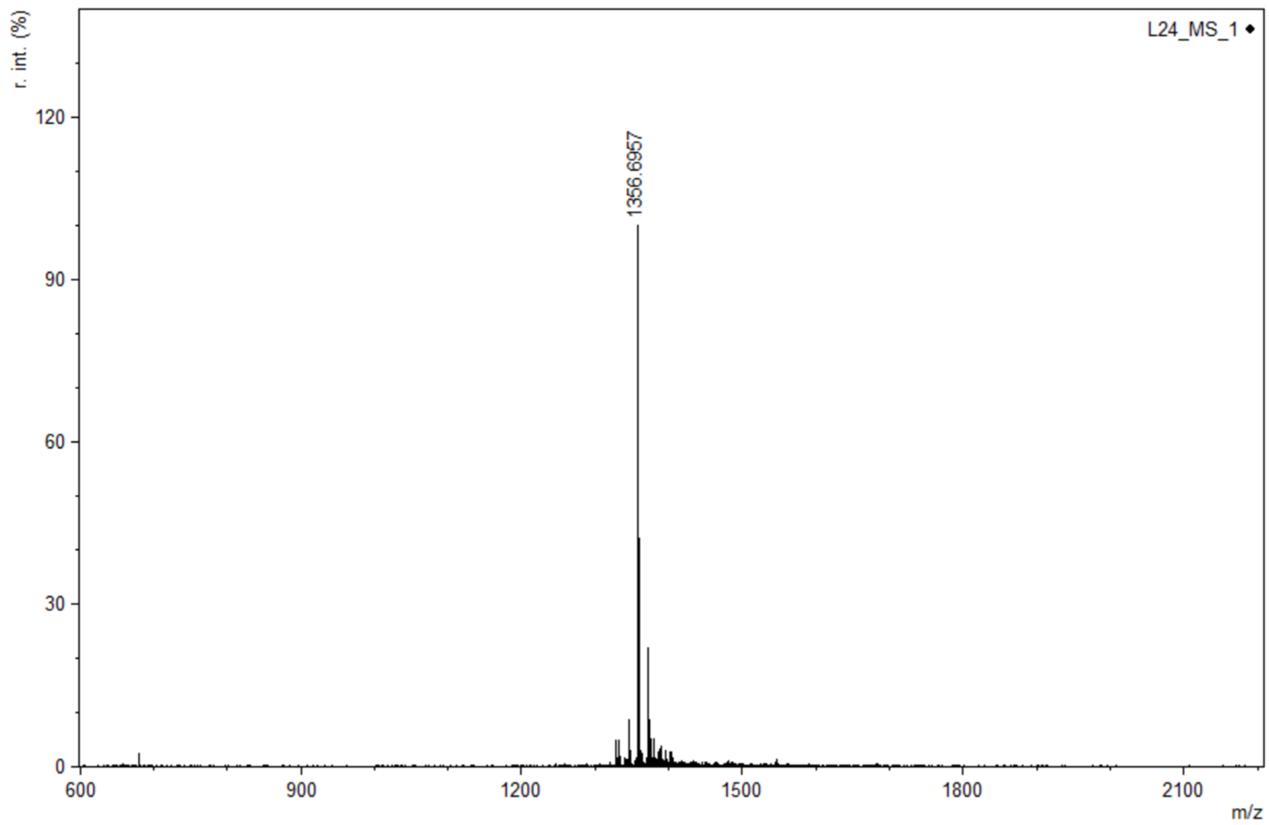

Figure S65. Mass spectrum of NT-B5 (MALDI-TOF). $[M+H]^+$ calcd 1356.74, found 1356.70.

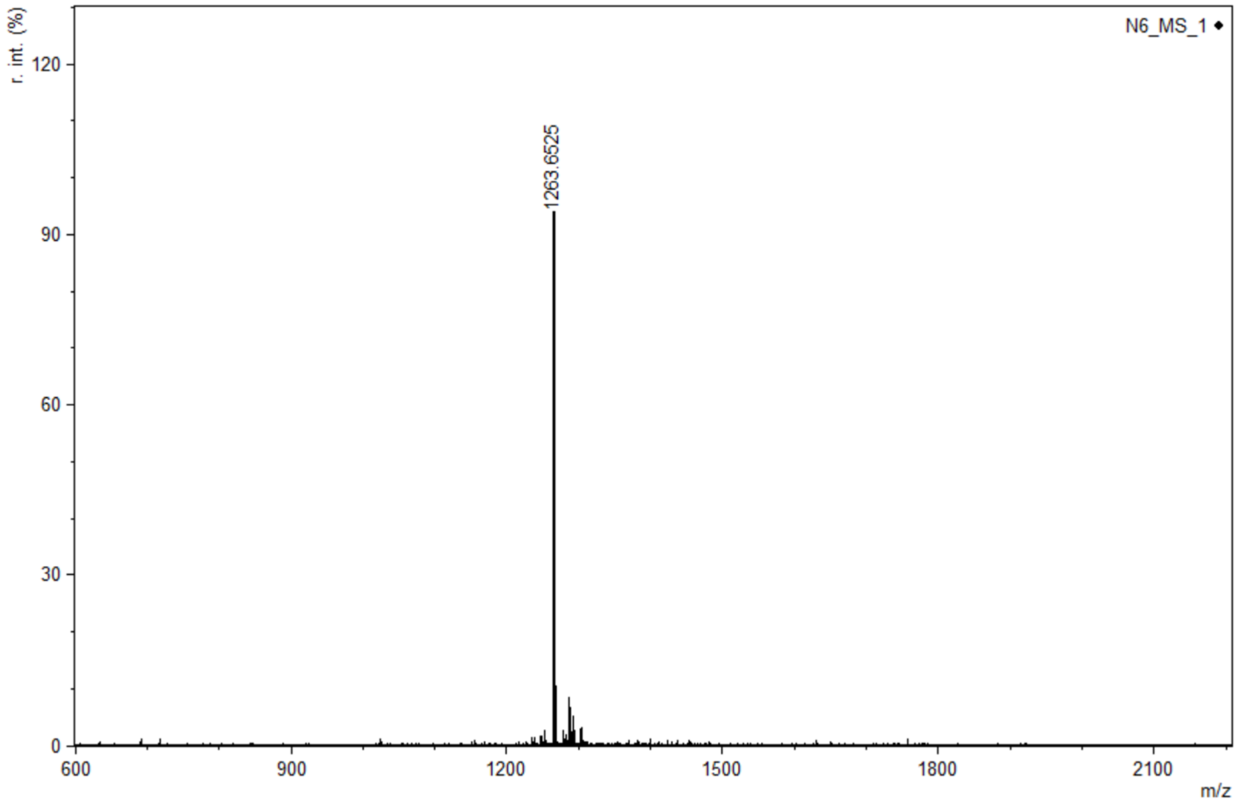

Figure S66. Mass spectrum of NT-B6 (MALDI-TOF). $[M+H]^+$ calcd 1263.70, found 1263.65.

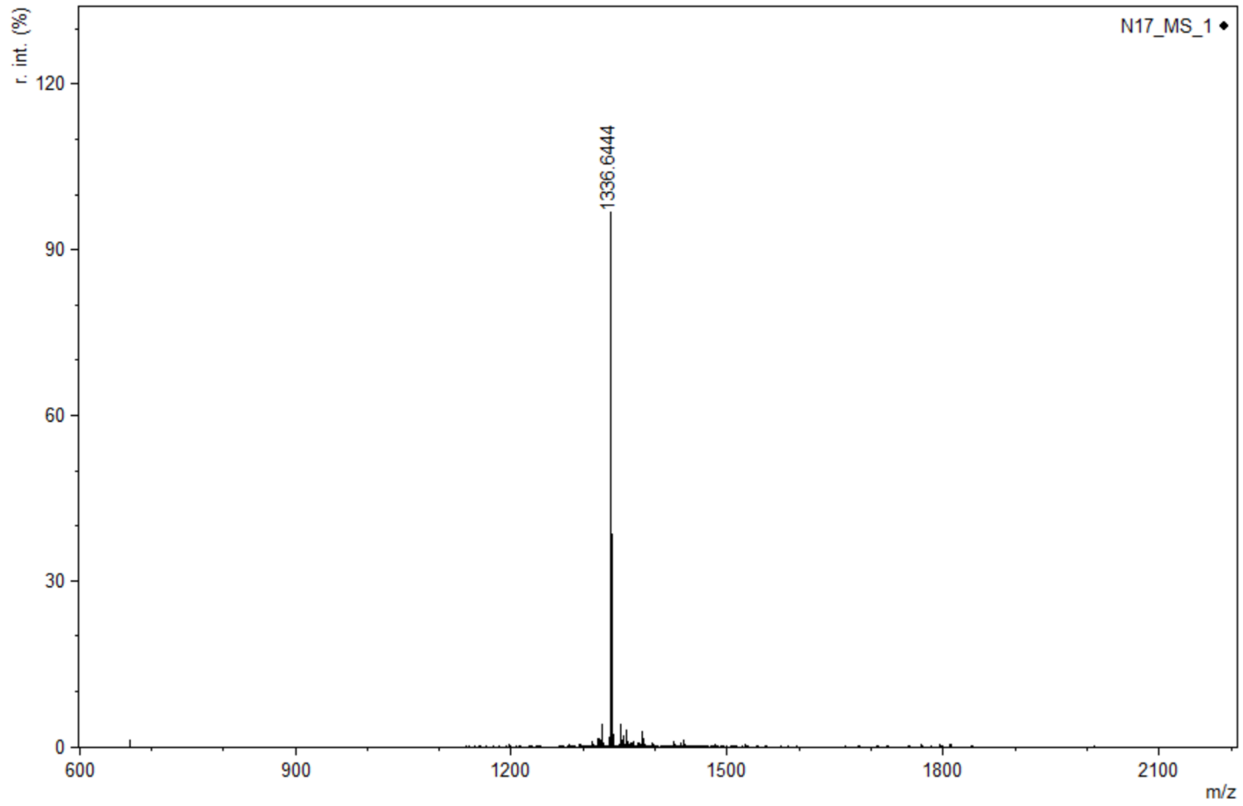

Figure S67. Mass spectrum of NT-B7 (MALDI-TOF). $[M+H]^+$ calcd 1336.69, found 1336.64.

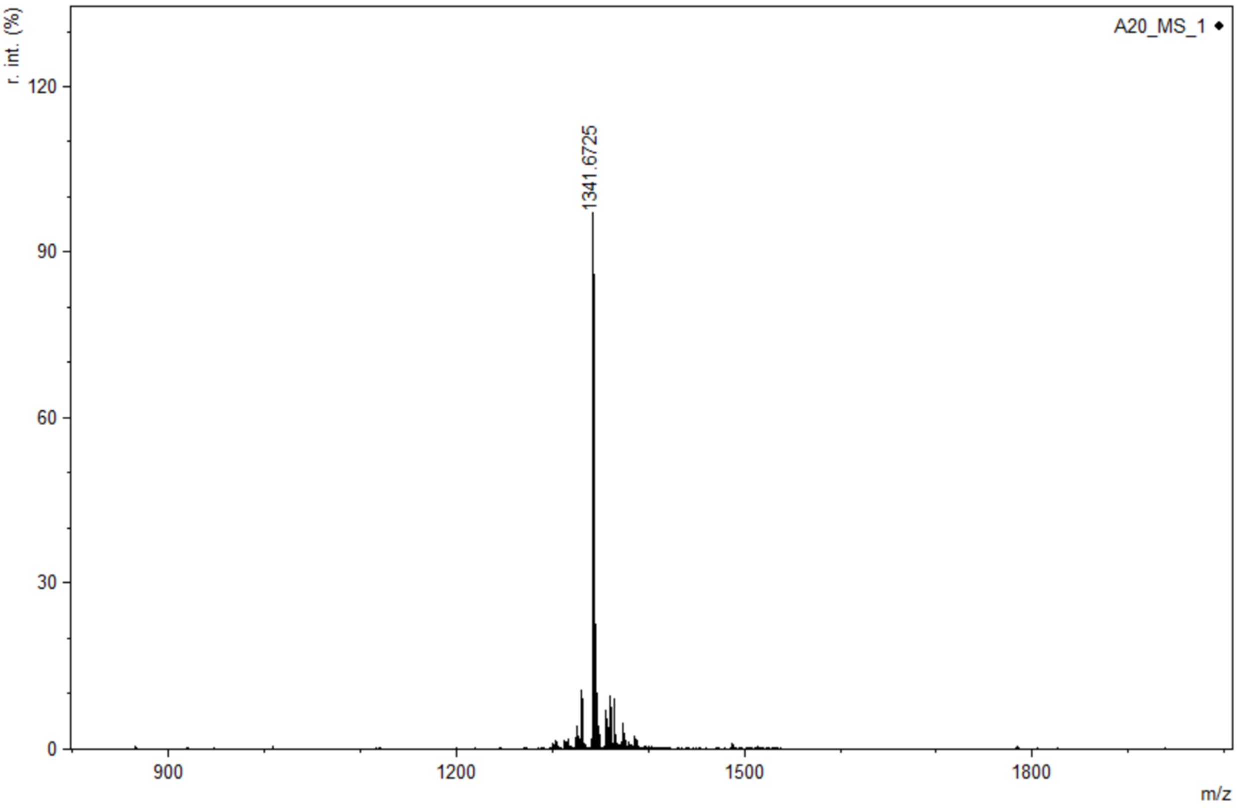

Figure S68. Mass spectrum of NT-B3 (MALDI-TOF). $[M+H]^+$ calcd 1341.74, found 1341.67.

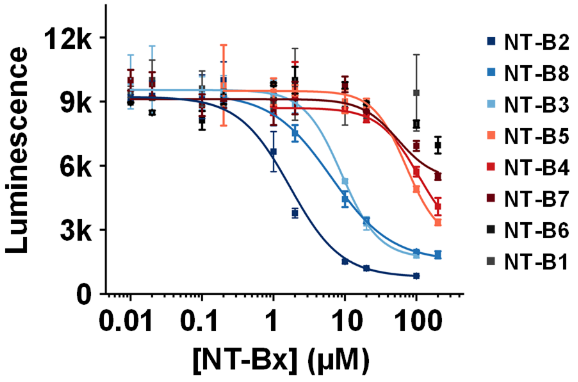

Figure S69. Competitive ELISA results for NT-B1~8 versus Biotin-PEG5-NT-B2. Recombinant MYC was used here as binding partner.

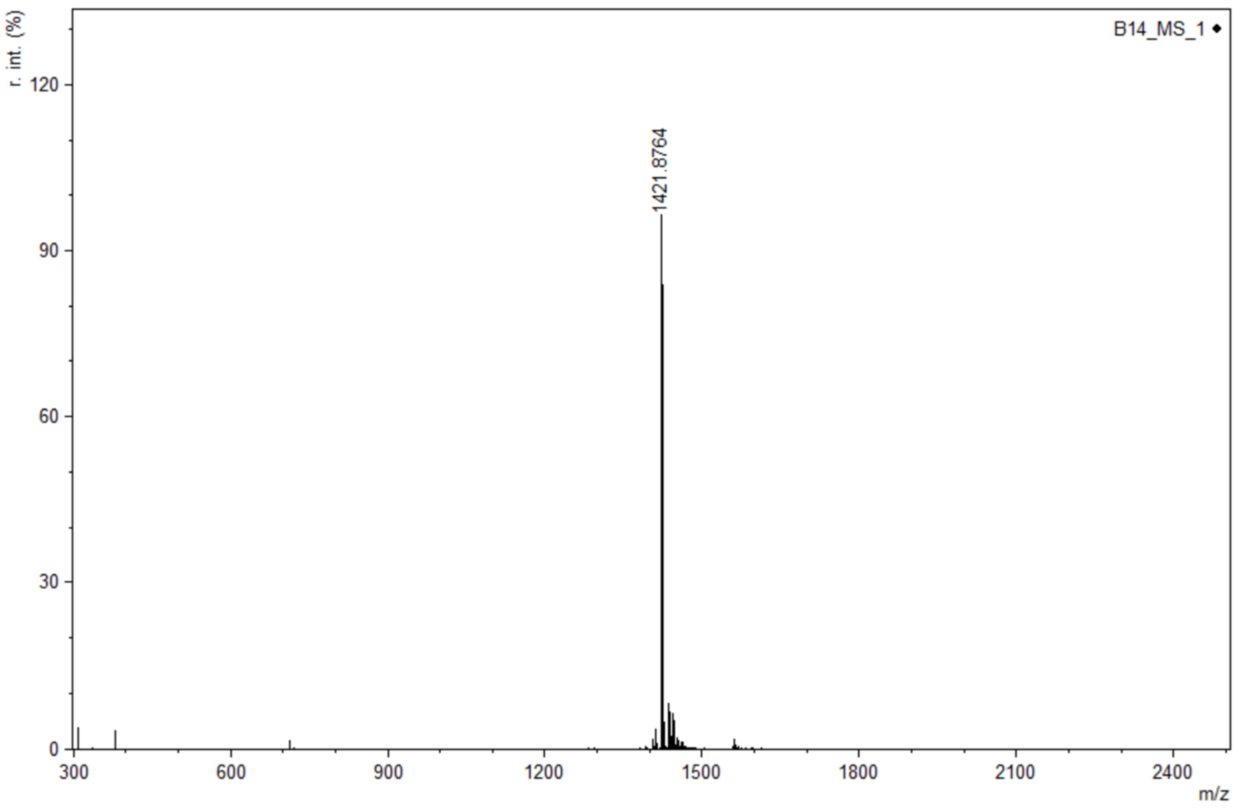

Figure S70. Mass spectrum of NT-B2 rac endo (MALDI-TOF). $[M+H]^+$ calcd 1421.86, found 1421.88.

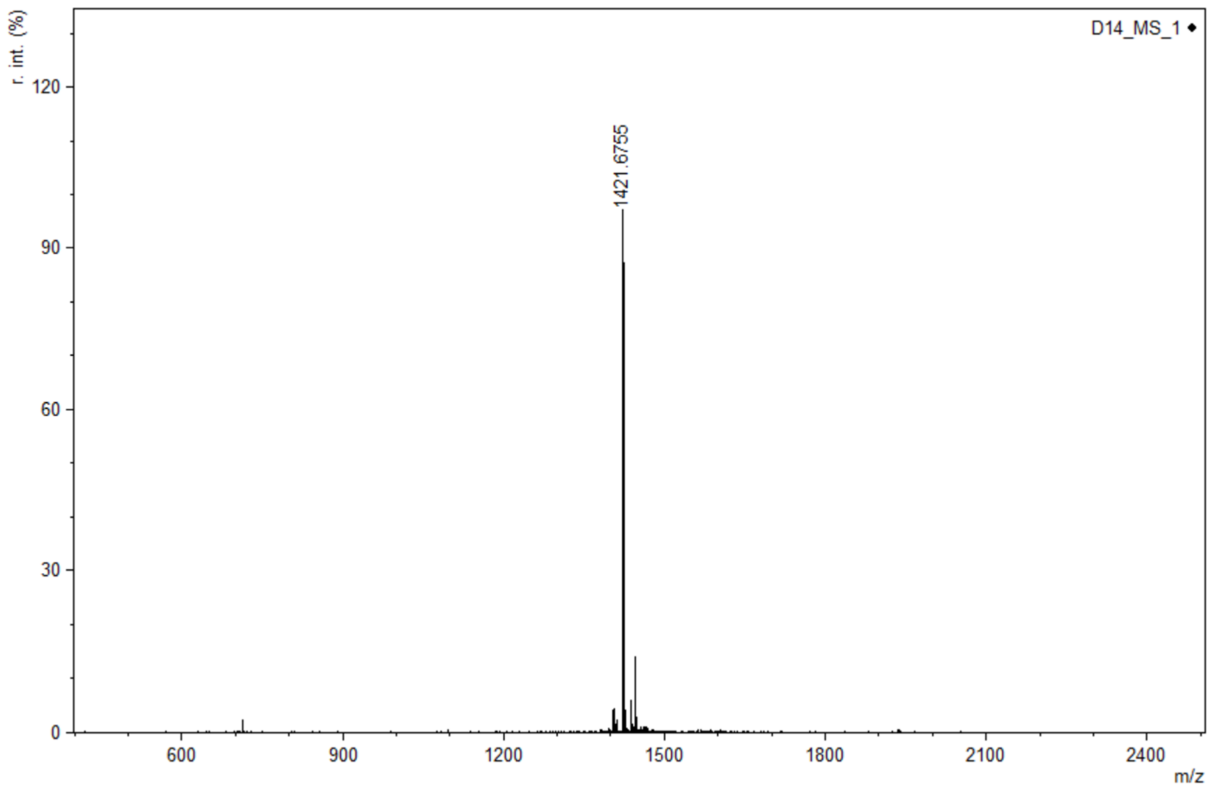

Figure S71. Mass spectrum of NT-B2 rac exo (MALDI-TOF). $[M+H]^+$ calcd 1421.86, found 1421.68.

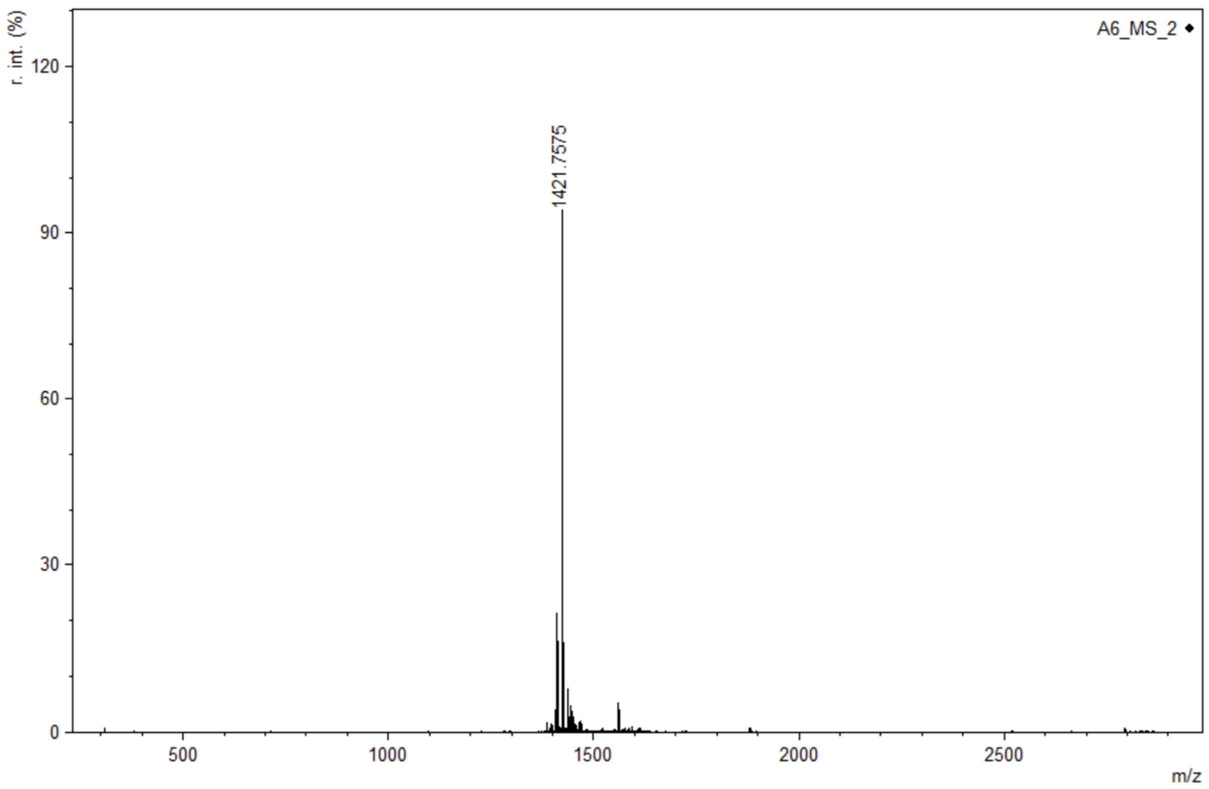

Figure S72. Mass spectrum of NT-B2R (endo) (MALDI-TOF). $[M+H]^+$ calcd 1421.86, found 1421.76.

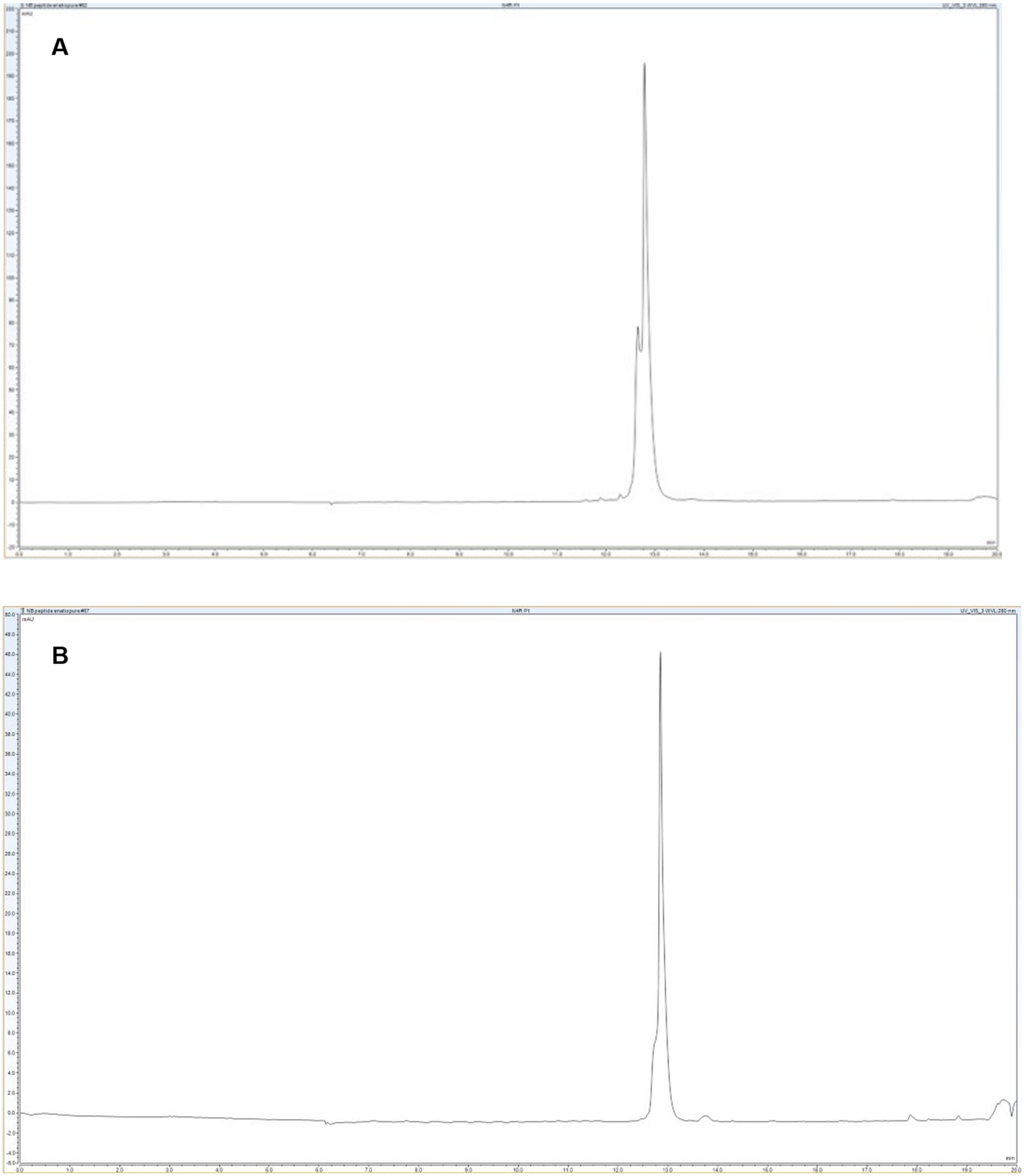

Figure S73. HPLC chromatograms of NT-B2R (endo) (280 nm). (**A**) NT-B2R (endo) after 1st round purification. (**B**) NT-B2R (endo) after 2nd round purification.

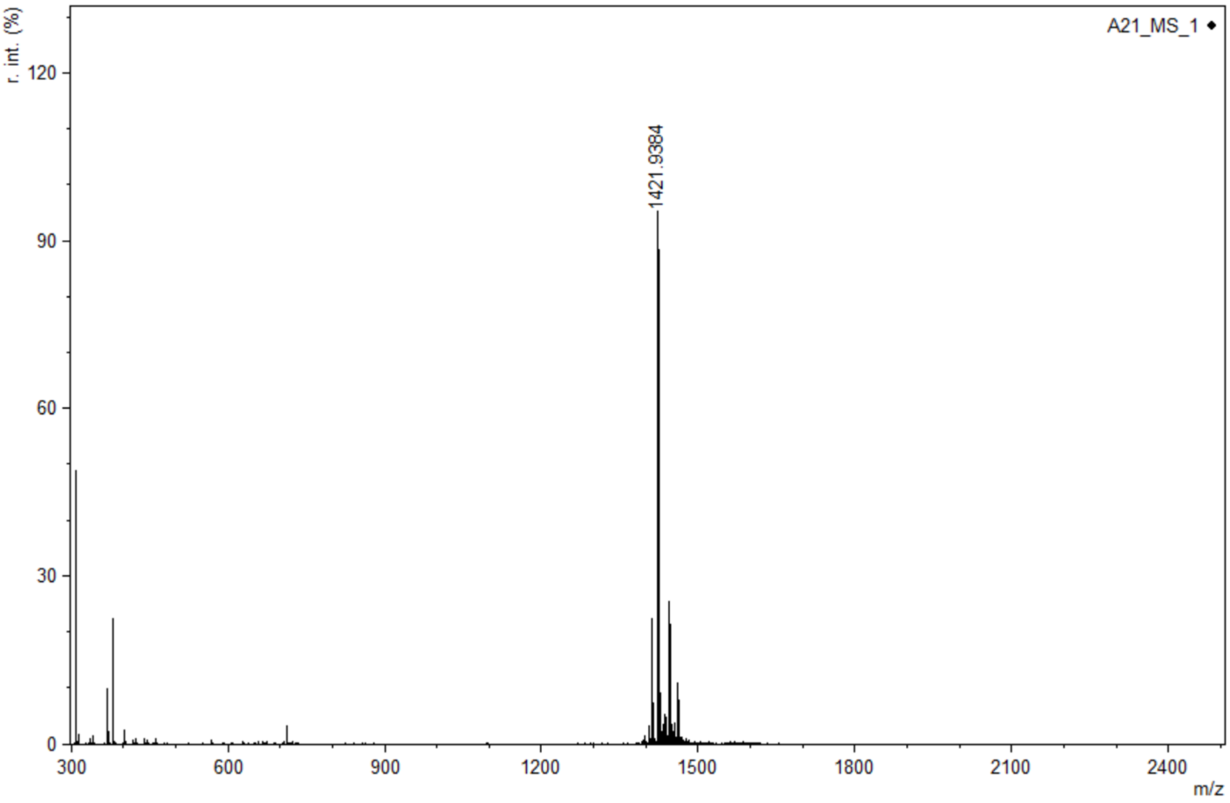

Figure S74. Mass spectrum of NT-B2S (endo) (MALDI-TOF). $[M+H]^+$ calcd 1421.86, found 1421.94.

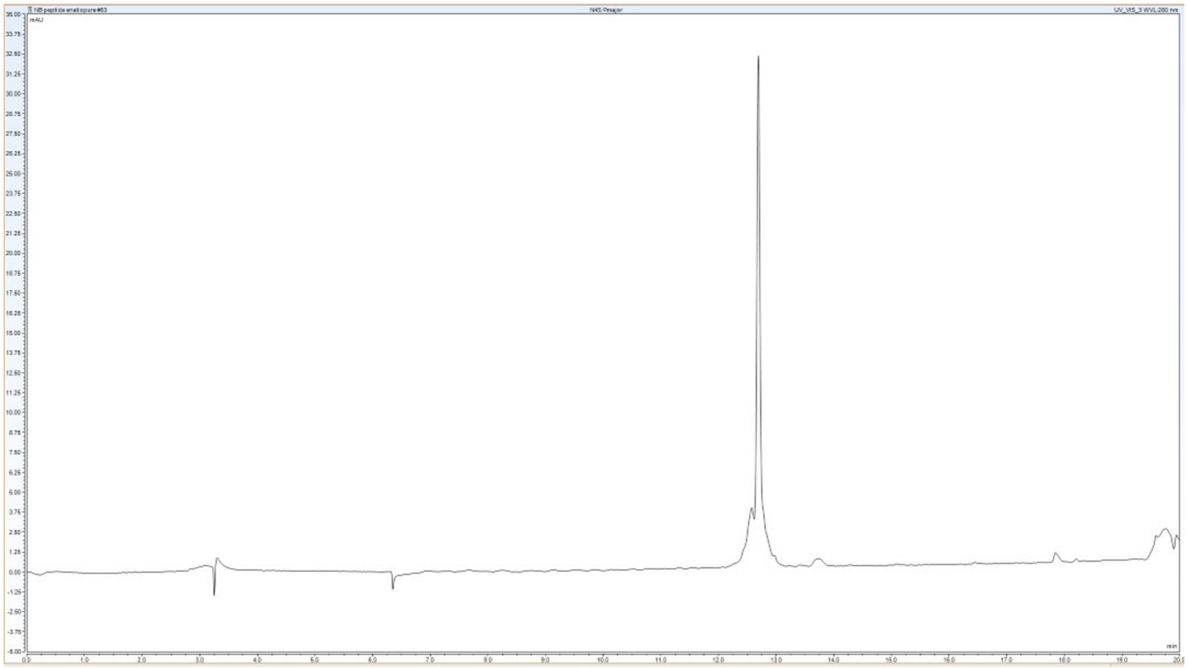

Figure S75. HPLC chromatogram of NT-B2S (endo) (280 nm).

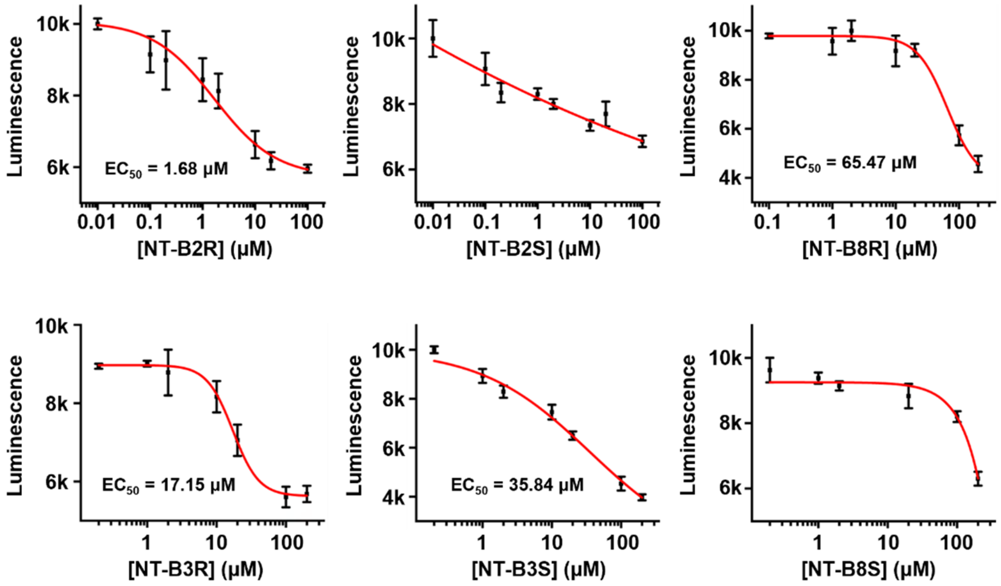

Hit	EC$_{50}$ (μM)
NT-B2R	1.68
NT-B2S	N.D.
NT-B3R	17.15
NT-B3S	38.84
NT-B8R	65.47
NT-B8S	N.D.

Figure S76. Competitive ELISA results for NT-B2,3, and 8 (endo R or endo S) versus Biotin-PEG5-NT-B2.

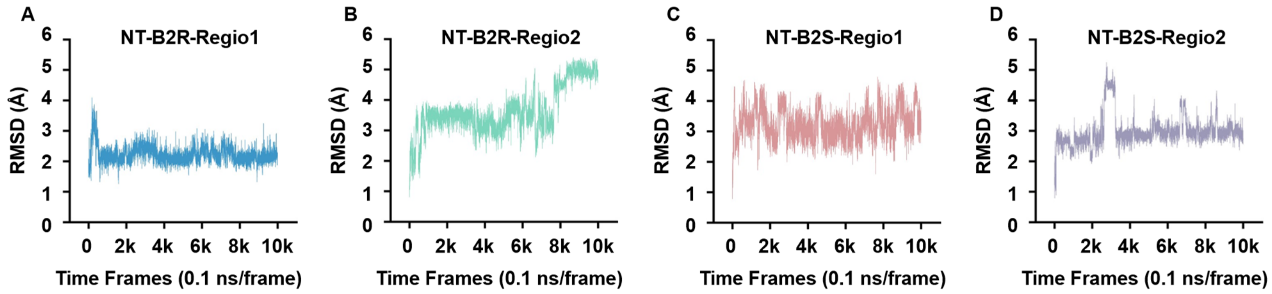

Figure S77. Molecular dynamics simulation analysis of NT-B2R and NT-B2S. Backbone RMSD analysis of NT-B2R and NT-B2S.

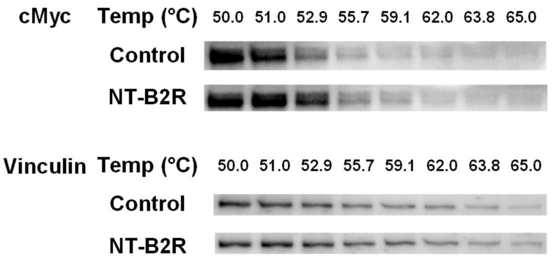

Figure S78. Cellular thermal shift assay (CETSA) results obtained using NT-B2R and U87 cell lysate. NT-B2R increased MYC's thermal stability, demonstrated by the increased melting temperature. The result here proved target engagement in cell lysate.

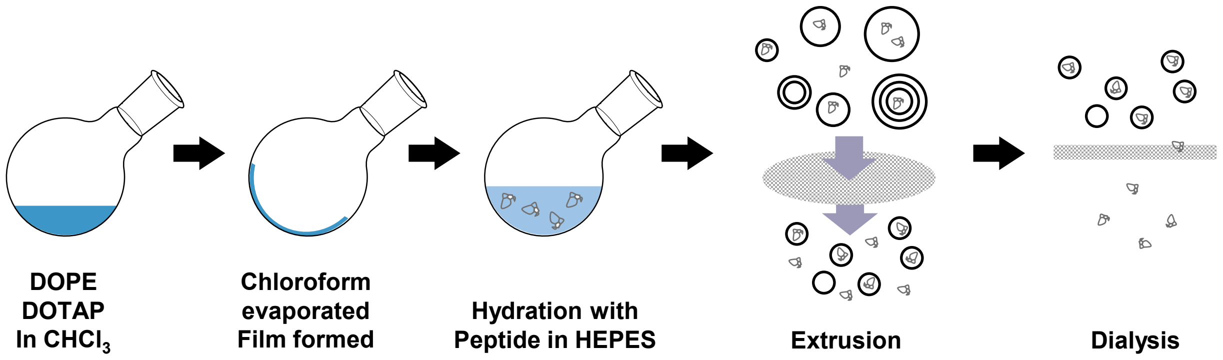

Figure S79. Schematics of the liposome preparation procedure.

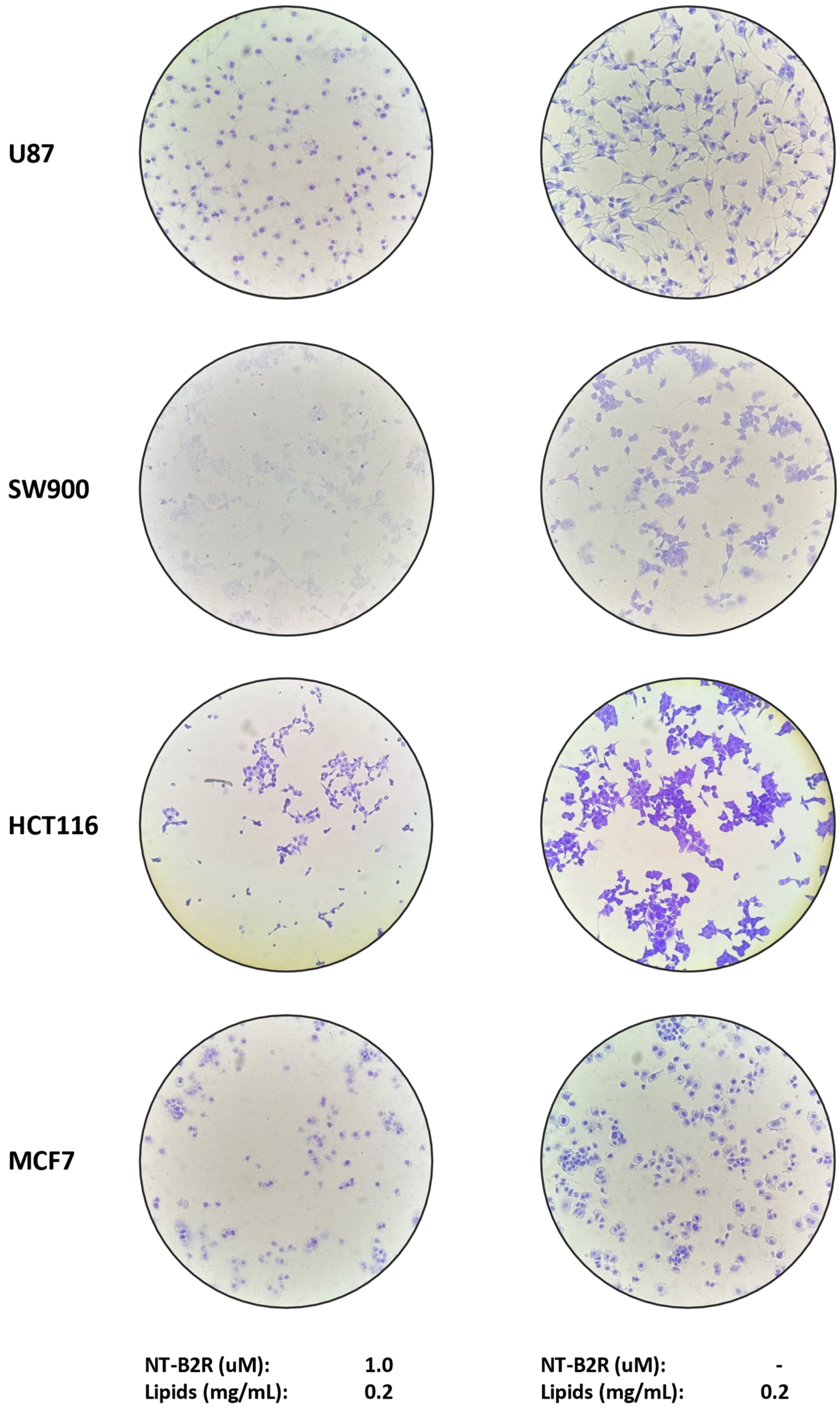

Figure S80. Crystal violet staining results showing the effects of NT-B2R treatment on U87, SW900, HCT116, and MCF7 cells.

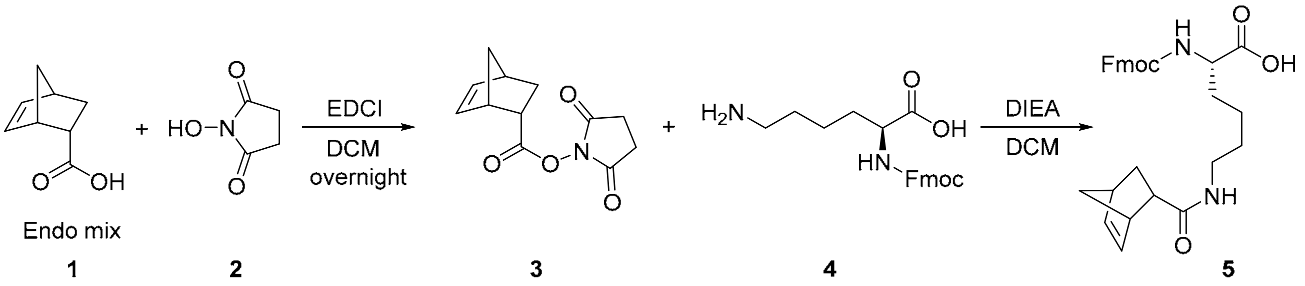

Scheme 1. Preparation of Fmoc-Lys(racemic endo Norbornene)-OH.

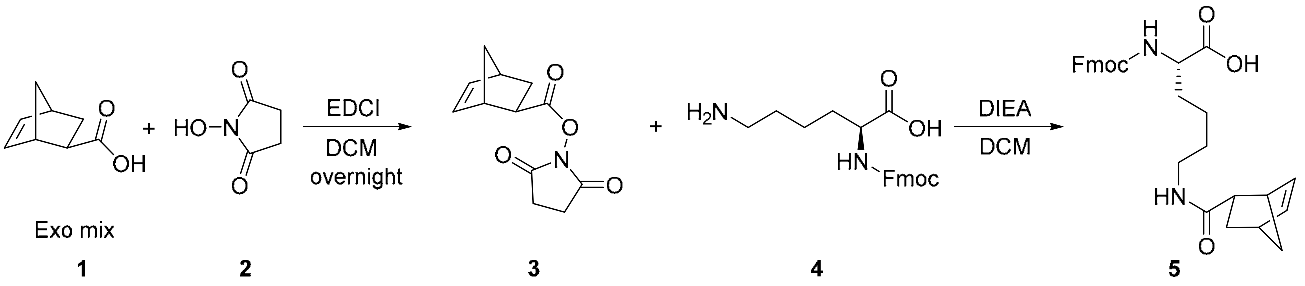

Scheme 2. Preparation of Fmoc-Lys(racemic exo Norbornene)-OH.

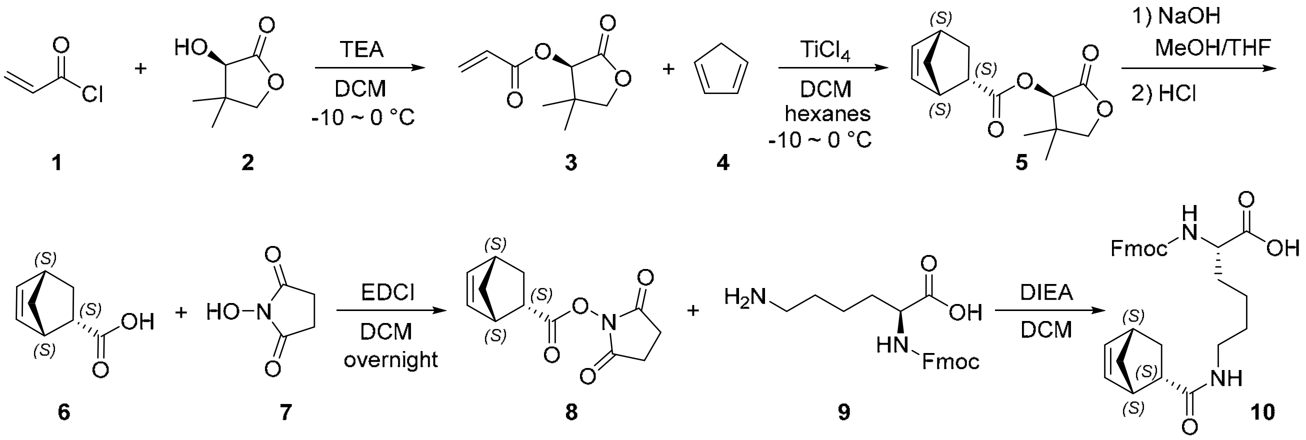

Scheme 3. Preparation of Fmoc-Lys(*S*,*S*,*S*-Norbornene)-OH.

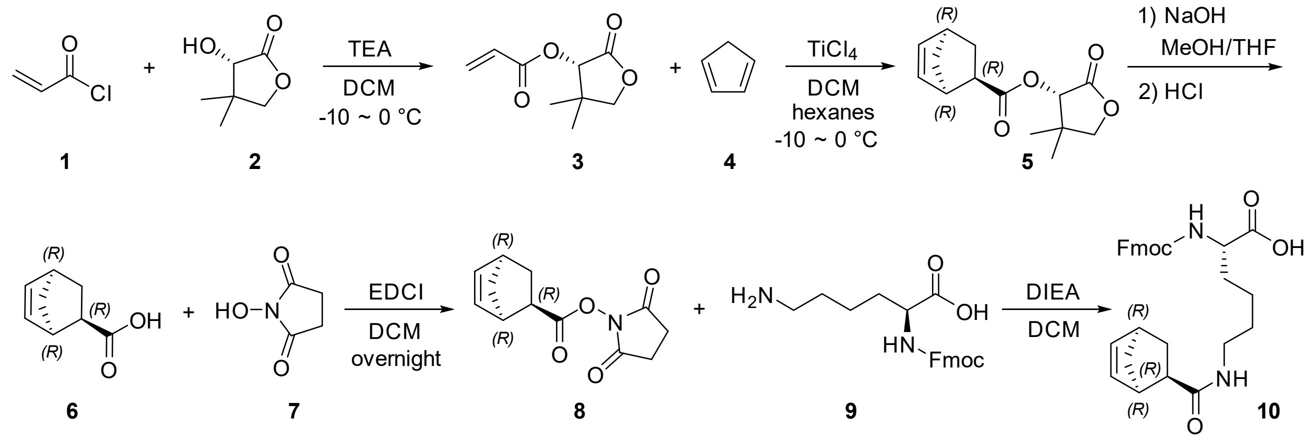

Scheme 4. Preparation of Fmoc-Lys(*R,R,R*-Norbornene)-OH.

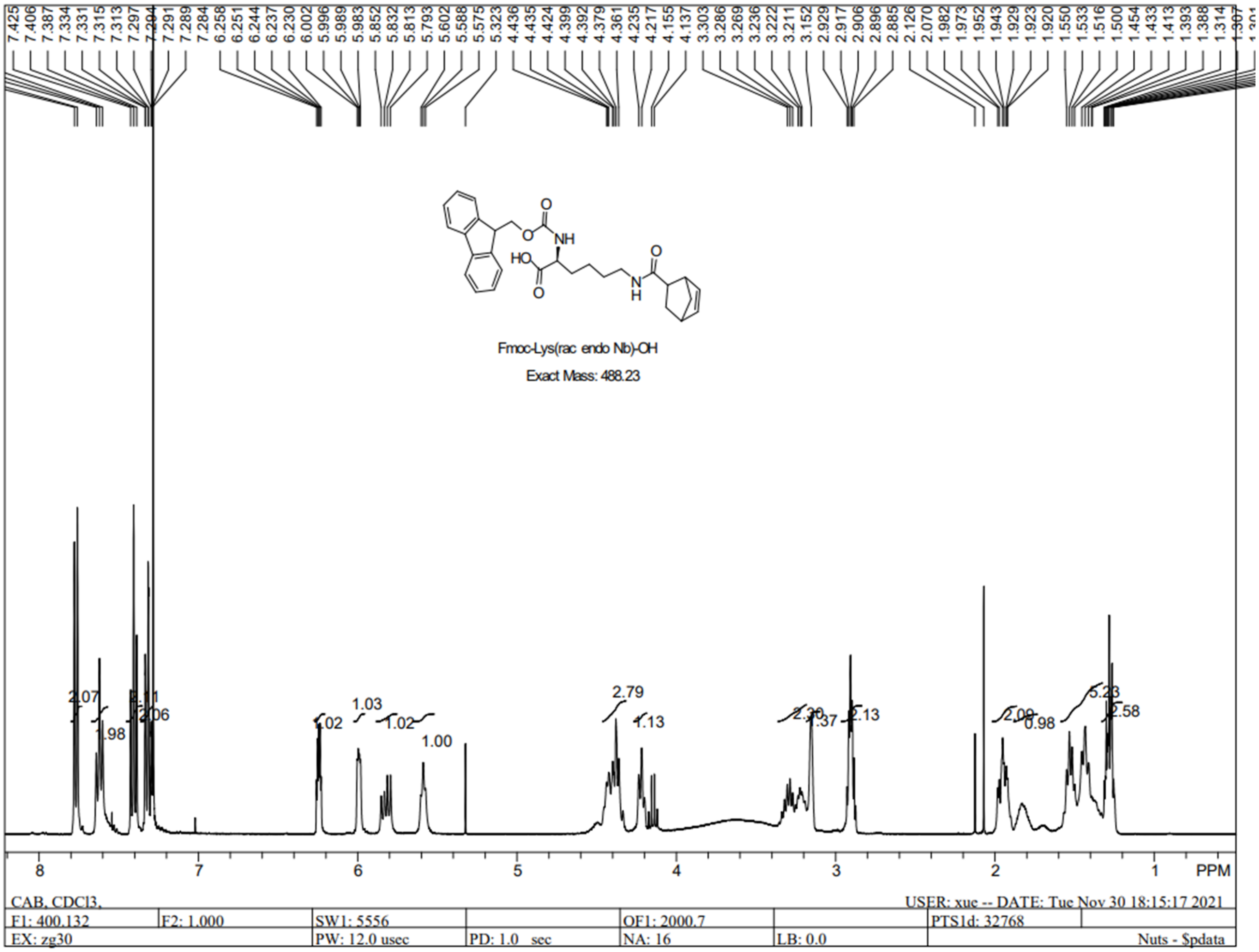

Figure S81. ^{1}H-NMR spectrum of Fmoc-Lys(rac endo Norbornene)-OH compound **5** (Scheme 1)

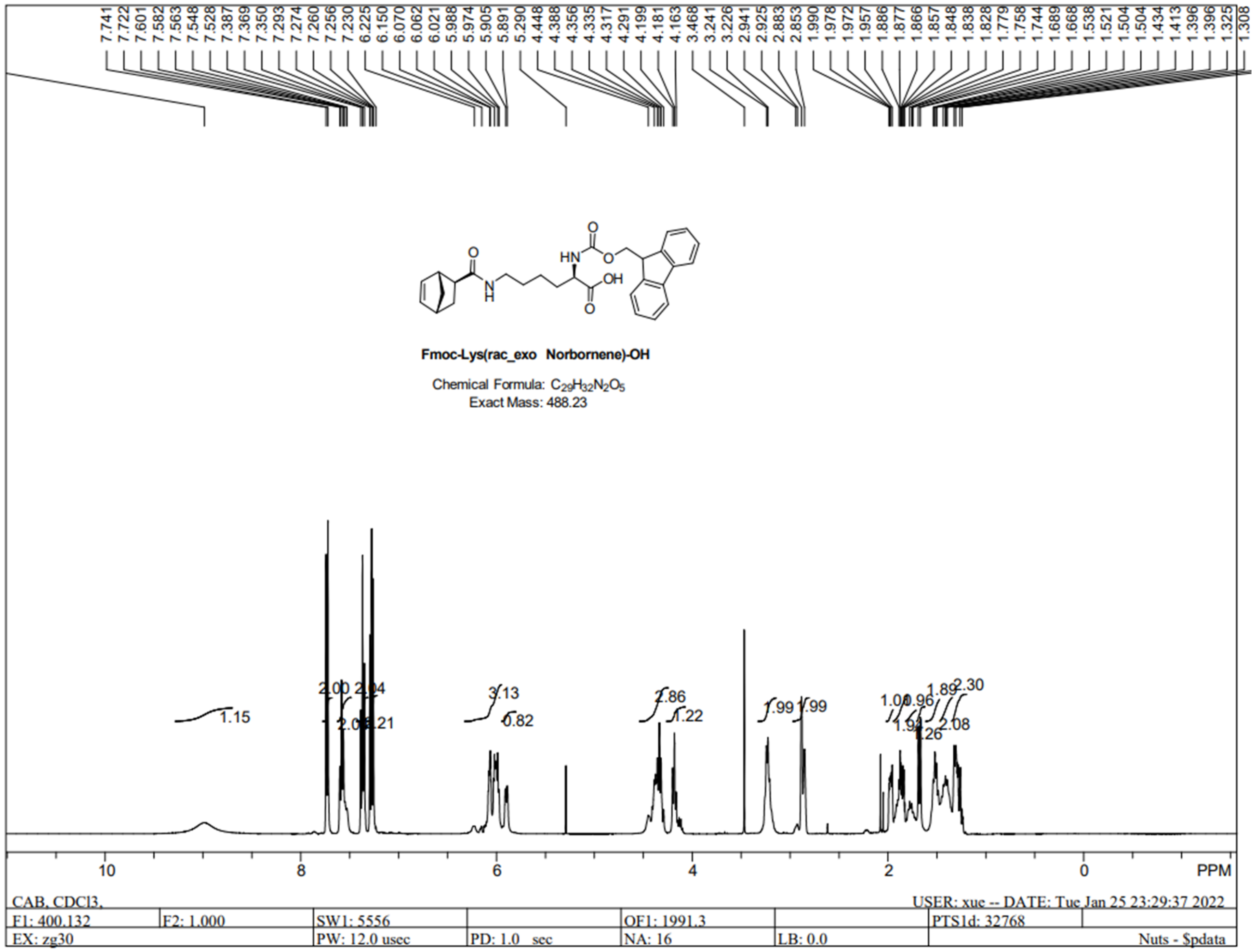

Figure S82. ^{1}H-NMR spectrum of Fmoc-Lys(rac exo Norbornene)-OH compound **5** (Scheme 2)

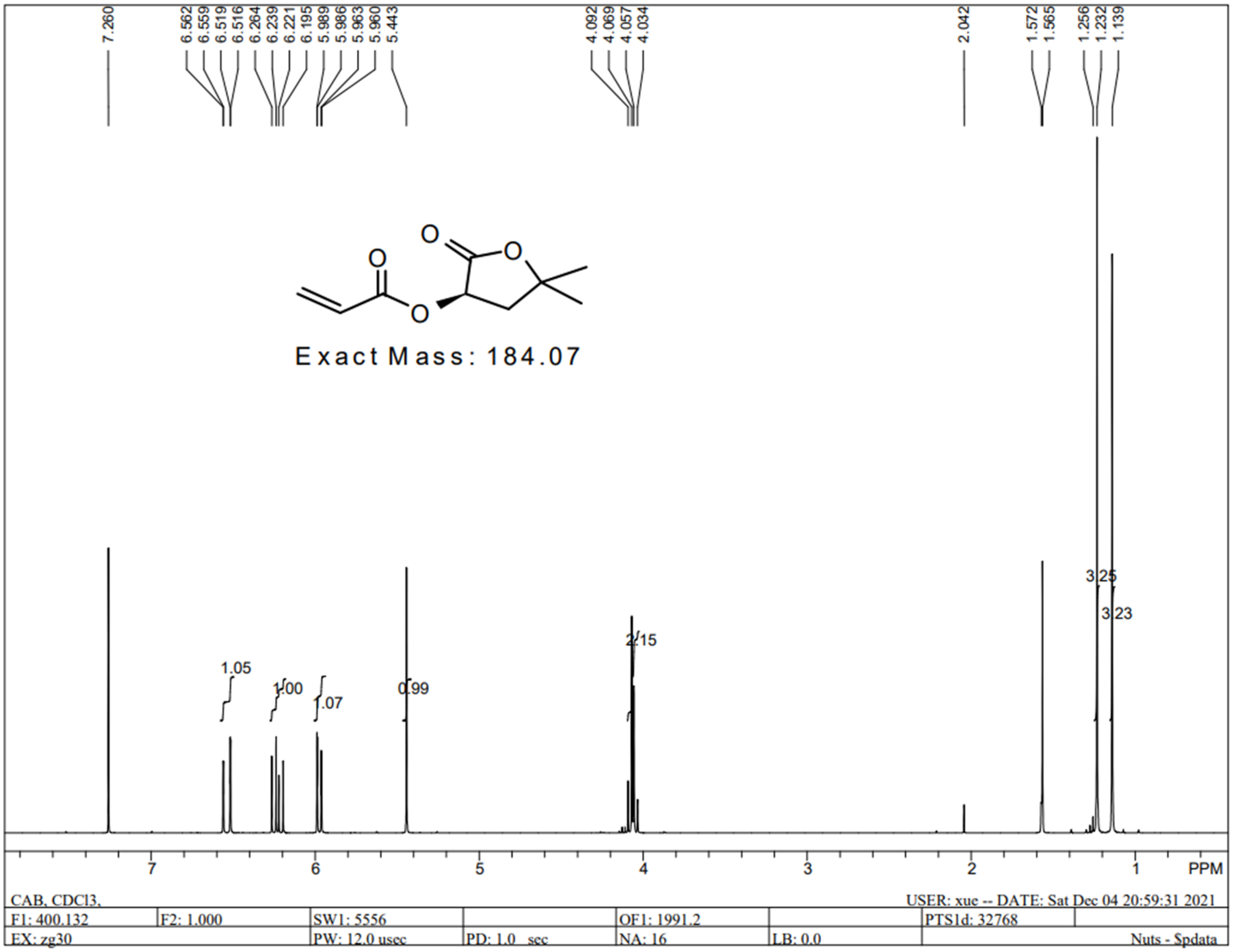

Figure S83. ^{1}H-NMR spectrum of D-pantolactone acrylate compound **3** (Scheme 3)

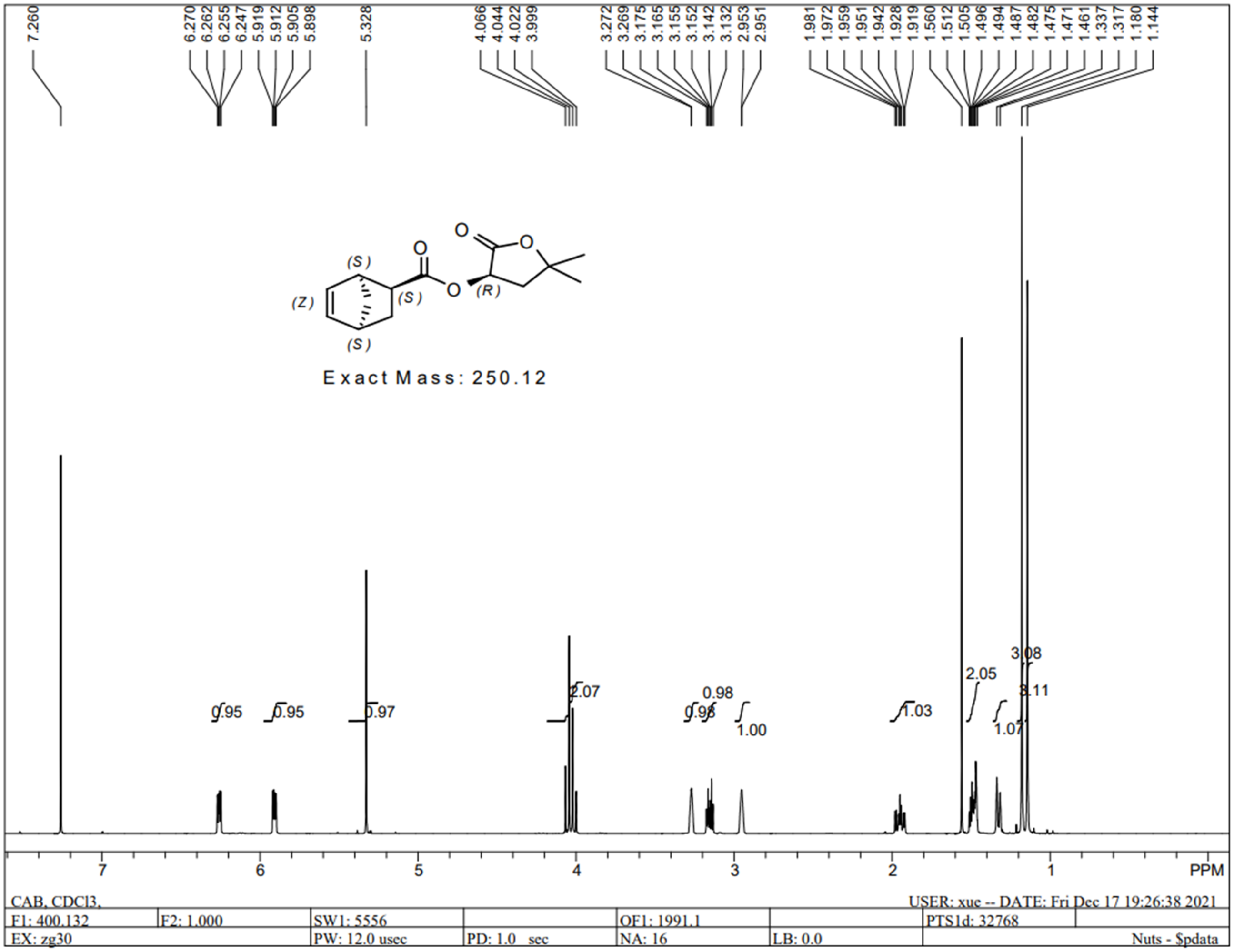

Figure S84. ^{1}H-NMR spectrum of Compound **5** in Scheme 3.

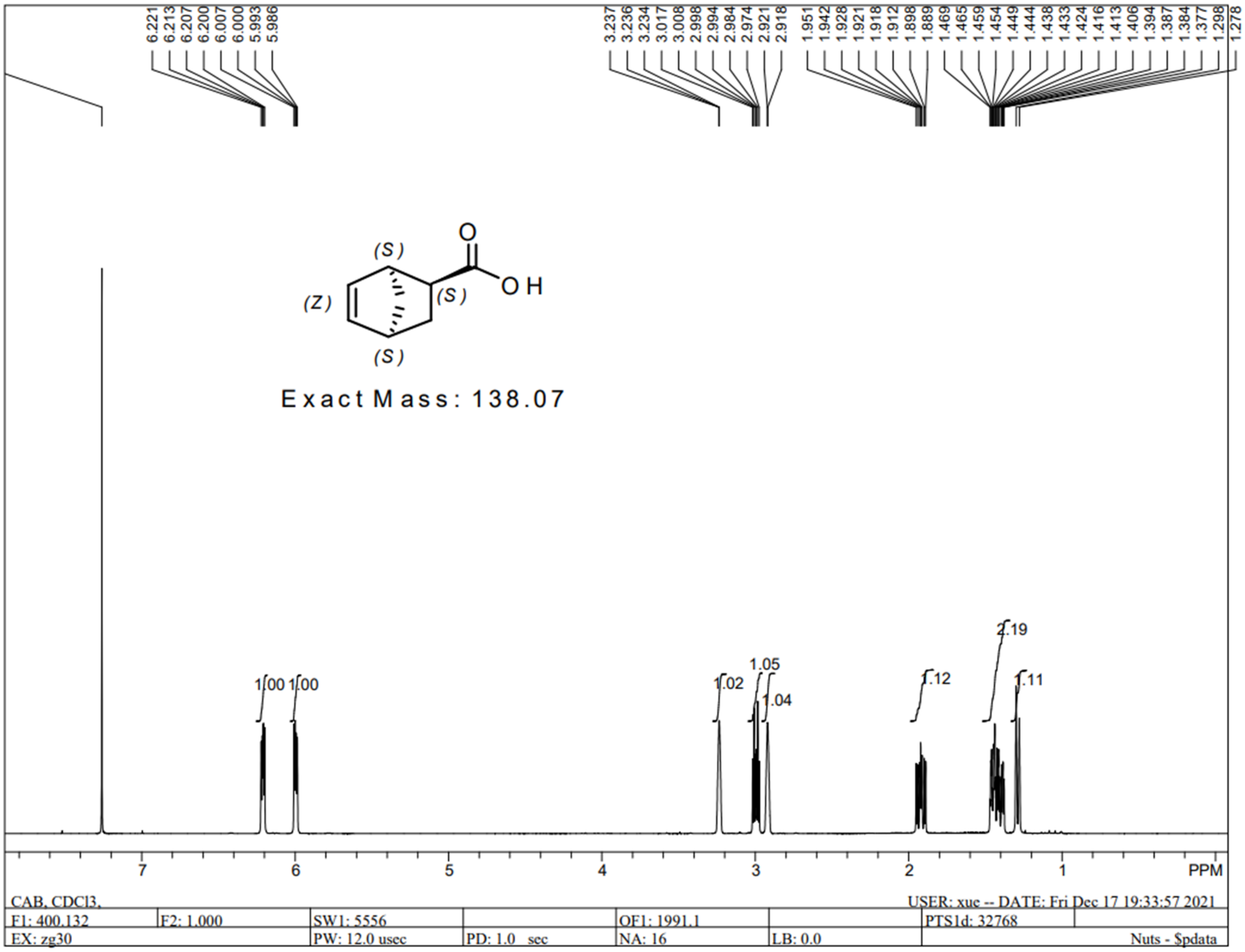

Figure S85. ^{1}H-NMR spectrum of Compound **6** ((1*S*,2*S*,4*S*)-5-norbornene-2-carboxylic acid) in Scheme 3.

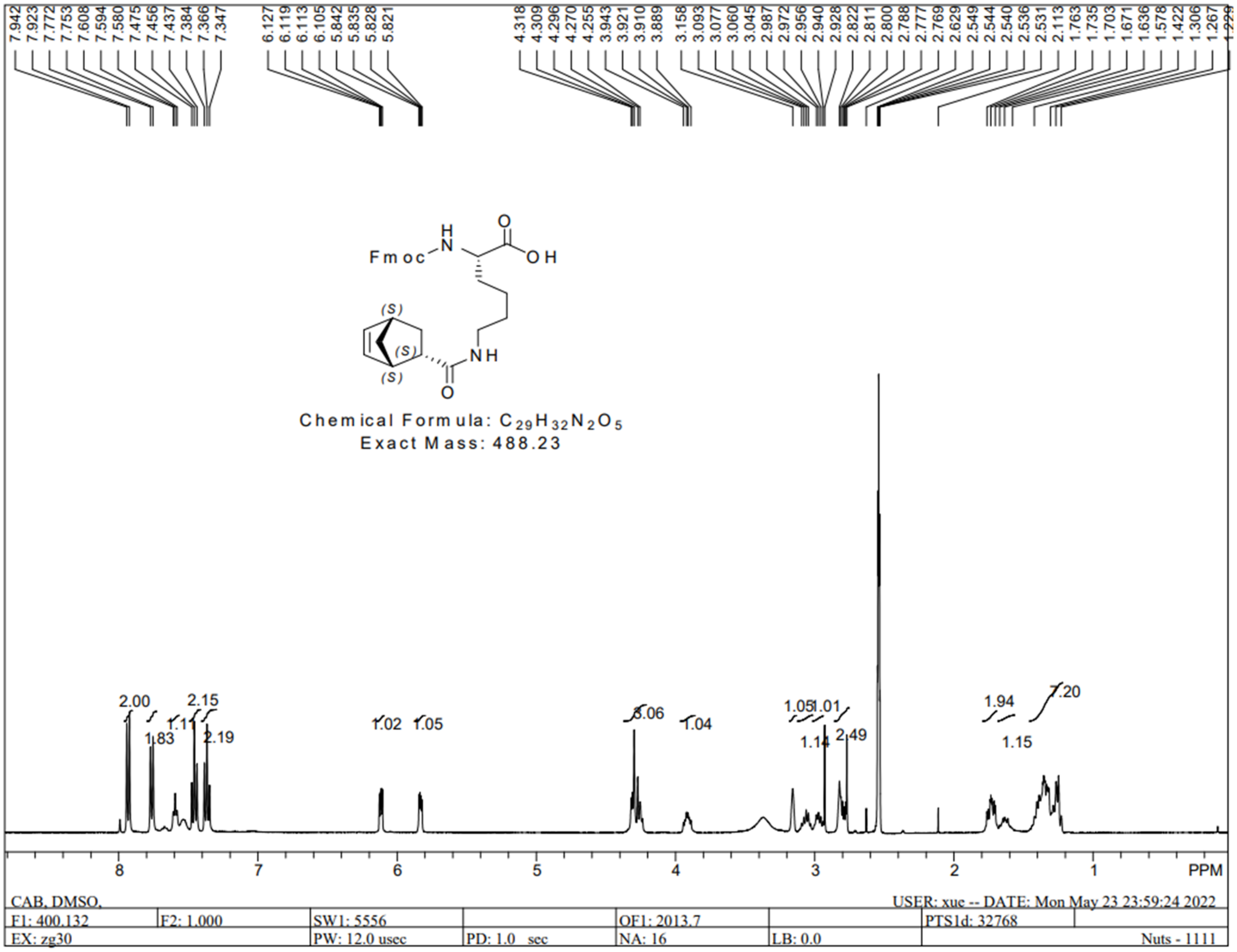

Figure S86. ^{1}H-NMR spectrum of Compound **10** (Fmoc-Lys((1*S*,2*S*,4*S*)-norbornene)-OH)) in Scheme 3.

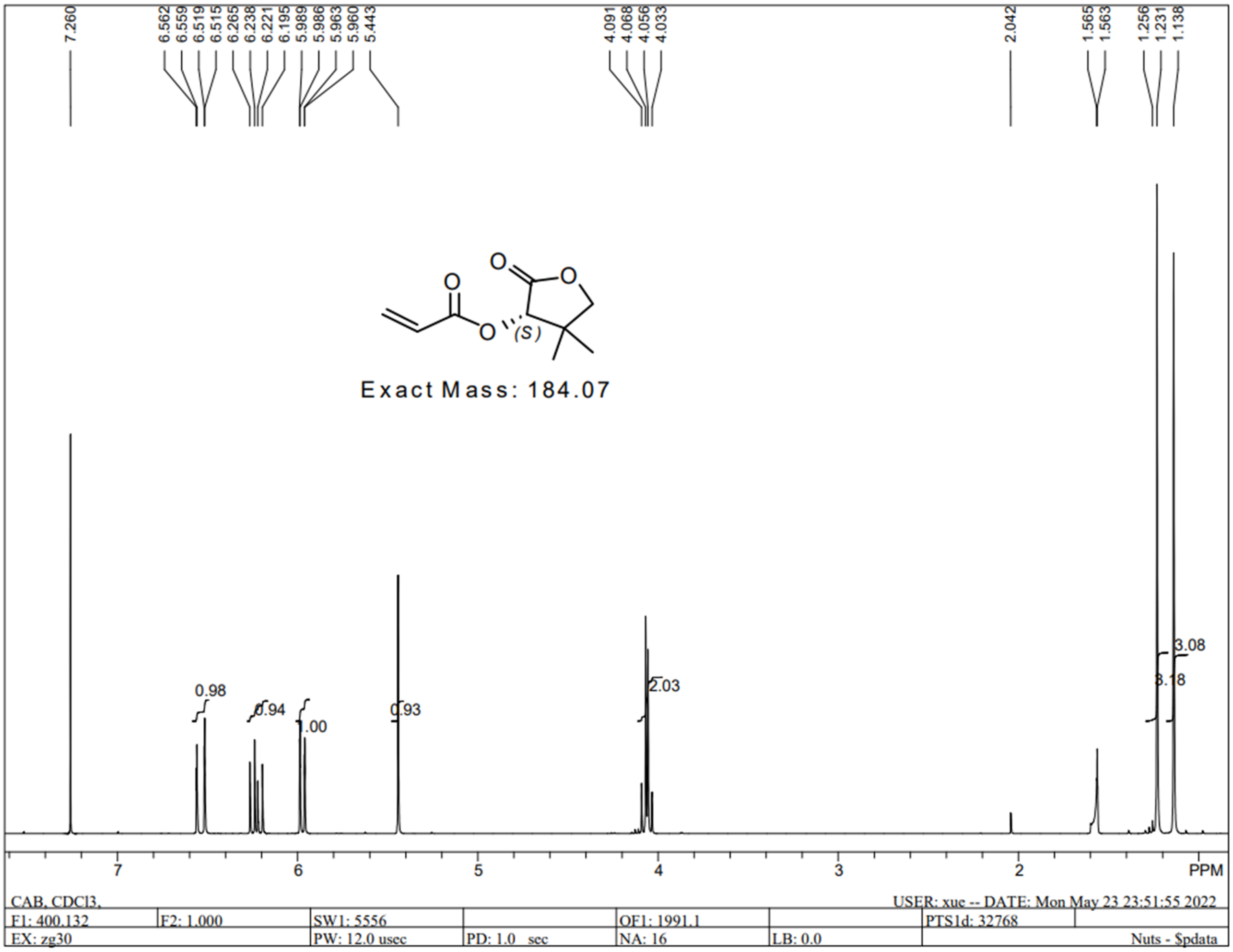

Figure S87. ^{1}H-NMR spectrum of L-pantolactone acrylate compound **3** (Scheme 4)

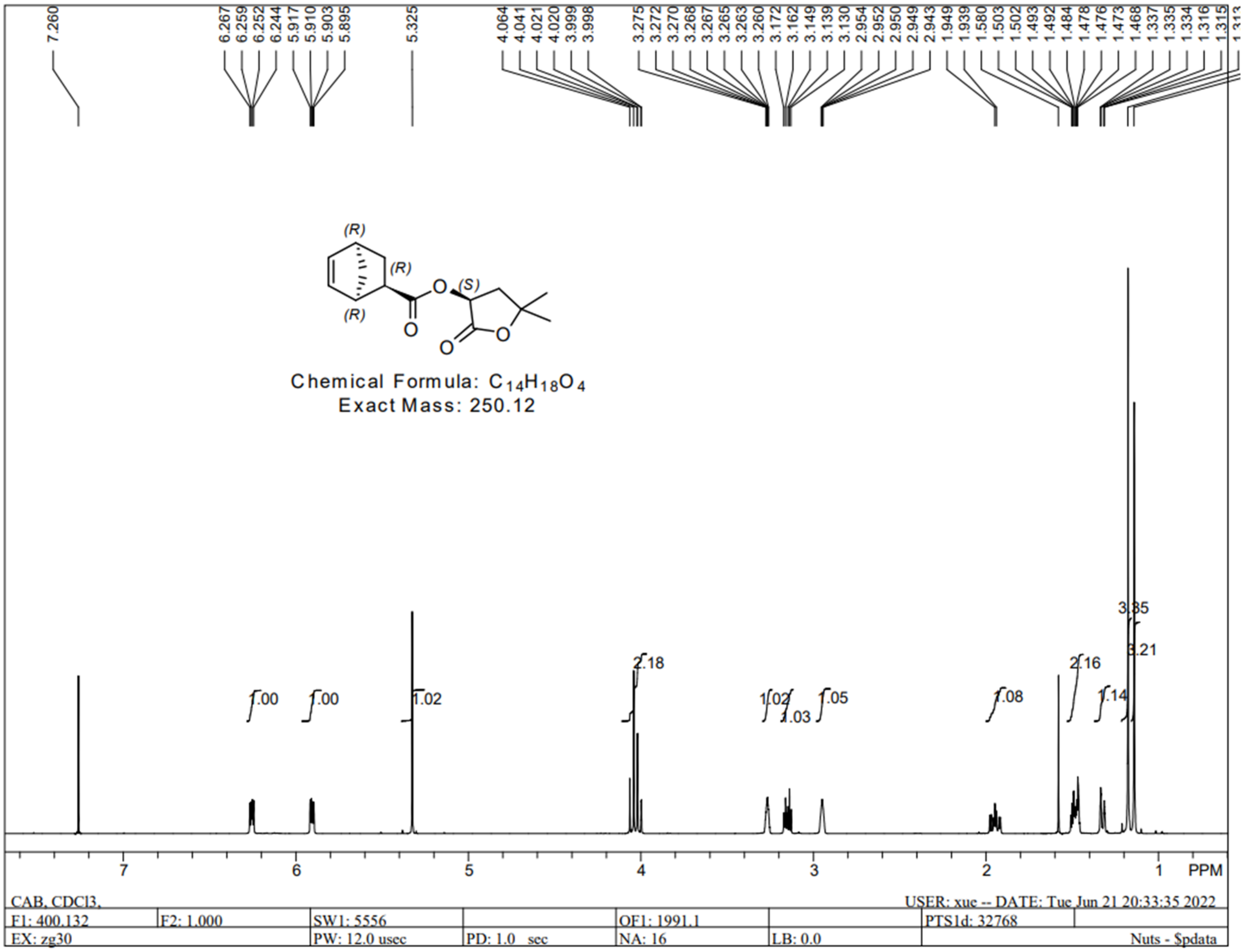

Figure S88. ^{1}H-NMR spectrum of Compound **5** in Scheme 4.

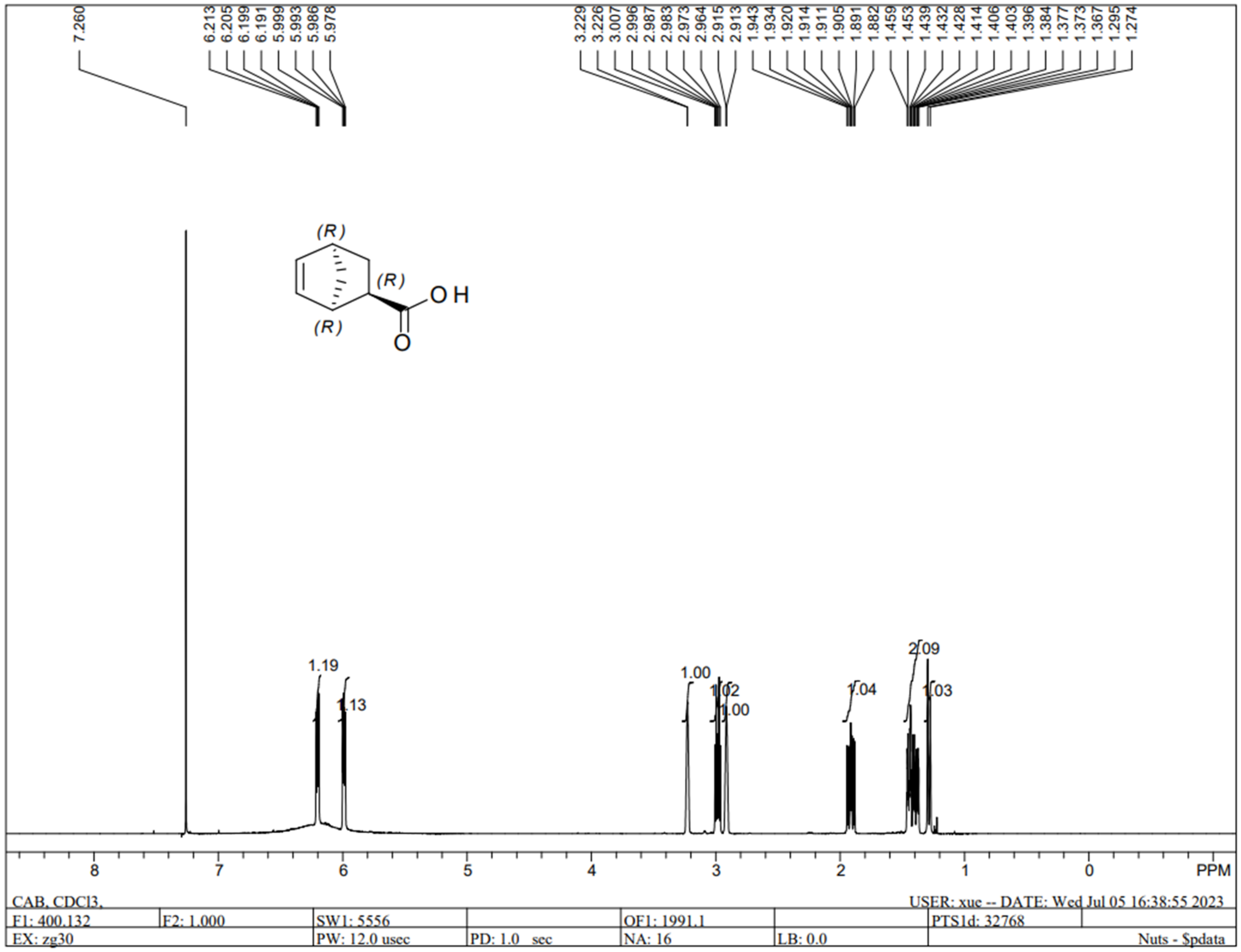

Figure S89. ^{1}H-NMR spectrum of Compound **6** ((1*R*,2*R*,4*R*)-5-norbornene-2-carboxylic acid) in Scheme 4.

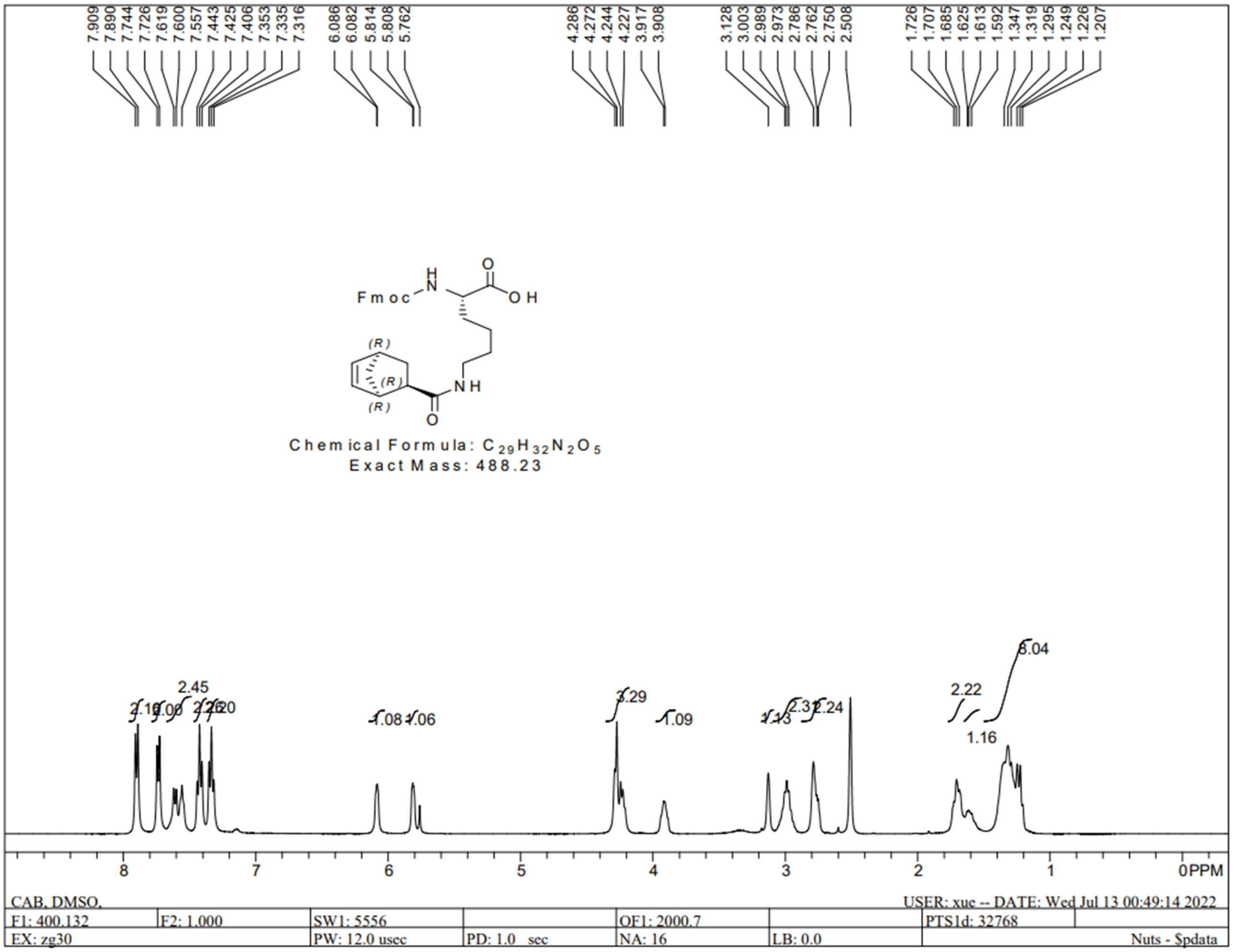

Figure S90. ^{1}H-NMR spectrum of Compound **10** (Fmoc-Lys((1*R*,2*R*,4*R*)-norbornene)-OH)) in Scheme 4.

Chemicals and reagents

(2-(1*H*-benzotriazol-1-yl)-1,1,3,3-tetramethyluronium hexafluorophosphate (HBTU, 99.6%), Fmoc-Lys(N_3)-OH (99.3%), 1-[Bis(dimethylamino)methylene]-1*H*-1,2,3-triazolo[4,5-b]pyridinium 3-oxid hexafluorophosphate (HATU, 99.7%), and D-Biotin (99.4%) were obtained from Chem-Impex (Wood Dale, IL).

Rink amide MBHA resin (0.678 mmol/g) and all Fmoc-protected canonical amino acids were purchased from AAPPTEC (Louisville, KY).

TentaGel S - NH_2 Resin (0.29 mmol/g) was purchased from RAPP POLYMERE (Tübingen, German).

Fmoc-PEG5-OH (98.68%) was obtained from BroadPharm (San Diego, CA).

Cuprous Iodide (CuI 98.0%) was ordered from Spectrum (New Brunswick, NJ).

Fmoc-Glu(OAll)-OH was purchased from Novabiochem (Latvia).

Fmoc-L-propargylglycine was ordered from Matrix Scientific (Columbia, SC).

Hoveyda-Grubbs Catalyst™ 2nd Generation (97%), Rac-EXO-5-NORBORNENECARBOXYLIC ACID, 97%, and Penicillin-Streptomycin (PS) were purchased from Sigma-Aldrich.

Tetrakis(triphenylphosphine)palladium(0) (99%) was ordered from STREM CHEMICALS (Newburyport, MA).

Diisopropylethylamine (DIEA, 99.5%) was obtained from ACROS (Germany).

Piperidine (99%) was purchased from Alfa Aesar (Ward Hill, MA).

Trifluoroacetic acid (TFA, 99%) was obtained from Oakwood Chemical (Estill, SC).

Phenylsilane (>97%) and Phenyl Isothiocyanate (>98%) were purchased from TCI (Japan).

Bovine serum albumin (BSA), N, N-Dimethylformamide (DMF, 99.9%), Acetonitrile (99.95%), Absolute methanol, HPLC-grade Acetonitrile (ACN), chloroform (with 0.75% ethanol), HEPES, and Dimethyl Sulfoxide (DMSO) were obtained from Fisher Scientific (Fair Lawn, NJ).

Sodium phosphate dibasic anhydrous (Na_2HPO_4, 99.6%) and Sodium phosphate monobasic monohydrate (NaH_2PO_4, 99.4%) were purchased from Fisher (China).

Sodium Chloride (NaCl) and TWEEN^{TM} 20 were purchased from Fisher (USA).

1,2-dioleoyl-3-trimethylammonium-propane (chloride salt) (DOTAP) and 1,2-dioleoyl-sn-glycero-3-phosphoethanolamine (DOPE) were purchased from Avanti Polar Lipids, Inc.

1X Dulbecco's Modified Eagle Medium (DMEM), Fetal Bovine Serum (FBS), and 0.25% Trypsin (with 2.21mM EDTA and 1X [-] sodium bicarbonate) were purchased from Corning Cellgro.

Ni-coated 96-well plate was purchased from GENO TECHNOLOGY, INC (SAINT LOUIS, MO).

Tetrazine acid was ordered from BIOCONJUGATE TECHNOLOGIES (SCOTTSDALE, AZ).

GloMelt Thermal Shift Protein Stability Kit was purchased from BIOTIUM INC. (FREMONT, CA).

D-(-)-Pantolactone and Rac-Endo-5-norbornenecarboxyxlic acid were ordered from COMBI-BLOCKS INC. (San Diego, CA).

S-(+)-Pantolactone was ordered from ENOVATION CHEMICALS LLC. (GREEN BROOK, NJ).

Synthesis of the bicyclic peptide library

Linear library precursor synthesis

The synthetic scheme is shown in Figure S7. The library was synthesized based on well-established protocols on Tentagel S NH_2 resin (loading capacity 0.29 mmol/g).[2] In a typical synthesis, the pre-swelled resin (2 g, 0.58 mmol) was first coupled with the ANP linker (1.25 g, 2.9 mmol), HATU (1.06 g, 2.78 mmol), DIEA (1.2 mL, 7.25 mmol) in DMF (20 mL). The mixture was agitated at room temperature for 2 hours and washed with DMF (5× 20 mL); followed by deprotection of Fmoc (20 mL, 20% v/v in DMF, 3×10 min) and washing with DMF (5× 20 mL). Then the ANP linked resin was coupled with Fmoc-Glu-(OAll)-OH (1.19 g, 2.9 mmol), HATU (1.06 g, 2.78 mmol), DIEA (1.2 mL, 7.25 mmol) in DMF (20 mL), the mixture was agitated at room temperature for 2 hours and washed with DMF (5× 20 mL); followed by deprotection of Fmoc (20 mL, 20% v/v in DMF, 3×10 min) and washed with DMF (5× 20 mL). The obtained 2 g resin was split evenly into 17 fractions and each fraction was coupled to a designated amino acid (Arg, His, Lys, Asp, Glu, Ser, Thr, Asn, Gln, Gly, Pro, Ala, Val, Leu, Phe, Tyr, Trp) using the following recipe: amino acid (0.16 mmol), HATU (0.15 mmol, 0.2 M in DMF, 768 uL), DIEA (0.4 mmol, 66 uL) in DMF (1.0 mL). The resins were agitated at room temperature for 2 hours to allow sufficient coupling. Later, all 17 fractions were washed with DMF and pooled together and undergone Fmoc deprotection. These split-pool processes were repeated for another 4 cycles. For the shared residues such as Fmoc-Glu(OAll)-OH, Fmoc-propargylglycine, and Fmoc-Lys(NB)-OH (50% racemic endo norbornene + 50% racemic exo norbornene), the coupling was performed in one pot with the same method as the ANP coupling.

Ring opening and ring closing metathesis (ROM-RCM)

The resin was first dried under vacuum and then mixed with Hoveyda-Grubbs 2nd generation catalyst (M720) (73 mg, 0.12 mmol) in a 100 mL flask, charged with argon for 3 times. De-oxygenated 1,2-dichloroethane (20 mL) was added and the mixture was stirred gently at 70 °C overnight. Another batch of M720 catalyst (73 mg, 0.12 mmol) was added and the reaction continued for an additional 16 hours. The resin was washed with DCM (20 mL × 5) and incubated with tris(hydroxymethyl)phosphine (0.2 M in isopropanol, 20 mL) at 80 °C for at least 12 hours. Then the resin was washed with (50% DMF in H_2O 20 mL×3), DMF (20 mL×3).[3]

Global deprotection

The library resin underwent an extra propargylglycine coupling following the same method mentioned above. After deprotection of the Fmoc, global deprotection was performed using a mixture of trifluoroacetic acid, triisopropylsilane and water (95:2.5:2.5 v/v, 20 mL) for 2 hours. The library was then washed with DMF (20 mL×3) and ready to use.

Library screening

Screening against the MYC epitope

The screening strategy is shown in Figure 2 in the manuscript. The bicyclic peptide library (pre-cleared following the procedure in ref [2] by Das et al, 1 M beads) resin was swelled in TBS for 2 hours, and blocked with 1% BSA-TBST at 4 °C overnight. The library was incubated with the epitope (10 μM in 0.1% BSA-TBST) at room temperature for 6 hours. Subsequently, it was washed with a) 0.1% BSA-TBST 3×5 min; b) TBST 3×5 min; c) TBS 3×5 min. The resin was then treated with guanidine HCl (6 M, pH = 2) at room temperature for 2 hours and washed with H_2O for 10 min × 5. The library was swelled again in 1% BSA-TBST at 4 °C overnight and then incubated with antibiotin-AP (1:1000 diluted in 1% BSA-TBST) at 4°C for 1 hour. Afterwards, it was washed with a) 0.1% BSA-TBST at 4°C 3×5 min; b) TBST at 4°C 3×5 min; c) TBS at 4°C 3×5 min; d) AP-buffer 1×5 min. The library was transferred to a Petri dish with 5 mL of AP buffer and developed with 10 mL of BCIP (13 μL in AP buffer) solution for 20 min. The reaction was then quenched with addition of concentrated HCl (50 μL / 1 mL AP-buffer). At this stage, the hit beads were colored blue and could be picked out from the library.

Linearization of the library and photocleavage

The synthetic scheme is shown in Figure S7. The candidate beads (color stripped) were incubated with phenyl isothiocyanate (2.5% v/v) in pyridine/water (1:1) and heated to 50 °C for 30 min. The solution was drained, and the beads were washed with DMF ×3, EtOAc ×3 and dried. The beads were agitated with a mixture of $Pd(PPh_3)_4$ (13 mg, 11 μmol), $PhSiH_3$ (111 μL, 1.3 mmol) in DCM (1 mL) at rt for 2 hours. The resulting dark solution was removed, and the beads were washed with a) DMF ×3, b) chelating solution (Sodium diethyldithiocarbamate (5% w/v), DIEA (5% v/v) in DMF) ×3, c) DMF ×3, d) 50% DMF in H_2O ×3, e) DMF ×3, f) EtOAc ×3 and dried. Then the beads were treated with TFA (degassed with argon) and heated to 50 °C for 10 min; after removal of the TFA, a 20% TFA in H_2O (v/v) solution was added and heated to 80 °C for 10 min. The beads were washed with DMF, EtOAc and dried.

For the photocleavage, 50 μL of THF (degassed with argon) was added in a 1.5 mL centrifuge tube with a single bead inside, the tube was irradiated under a UV lamp (Llak-Ray B-100A high intensity uv lamp, 100 W, 365 nm) at rt for 20 min. The resulting solution was removed by a SpeedVac concentrator (Savant SPD121P), the residue was dissolved in 0.5 μL of CHCA solution (4 mg/mL in 50% acetonitrile in H_2O, with 0.1% TFA) and spotted on a MALDI plate, ready for MALDI-TOF. mMass version 5.0 was utilized for spectra analysis and de novo sequencing[4].

Unnatural amino acid synthesis

Synthesis of Fmoc-Lys(racemic endo norbornene)-OH (Scheme S1)

A mixture of endo mix 5-norbornene-2-carboxylic acid (compound **1**, 2.5 g, 18.1 mmol), Succinic acid (compound **2**, 2.92 g, 25.3 mmol), EDCI (4.51 g, 23.5 mmol) in DCM (50 mL) was stirred at rt. Overnight. The resulting mixture was diluted with DCM 50 mL, and washed with saturated Na_2CO_3 solution, water, and brine. The organic phase was dried over Na_2SO_4 and concentrated by rotavap. The crude was obtained as white solid which was used directly in the next step.

A mixture of Fmoc-L-Lys-OH (1.84 g, 5.0 mmol), compound **3** (1.41 g, 6.0 mmol) from last step, and DIEA (1.6 mL, 10 mmol) in DCM (50 mL) was stirred at rt for 48 hours. The reaction was quenched with 0.5 N HCl 30 mL, and the mixture was partitioned between DCM and water. The organic phase was washed with water, brine, and dried over Na_2SO_4. The crude product was purified by column chromatography on silica gel with DCM/MeOH 15:1 to 10:1 to give the product as white solid (1.8 g, Yield 75%). ^{1}H NMR (400 MHz, $CDCl_3$) δ 7.74 (d, *J* = 7.5 Hz, 2H), 7.60 (t, *J* = 7.8 Hz, 2H), 7.38 (t, *J* = 7.5 Hz, 2H), 7.33 – 7.26 (m, 2H), 6.22 (dt, *J* = 5.6, 2.8 Hz, 1H), 6.00 – 5.94 (m, 1H), 5.80 (dd, *J* = 15.5, 8.0 Hz, 1H), 5.61 – 5.49 (m, 1H), 4.56 – 4.26 (m, 3H), 4.19 (t, *J* = 7.1 Hz, 1H), 3.35 – 3.16 (m, 2H), 3.13 (s, 1H), 2.94 – 2.81 (m, 2H), 2.00 – 1.85 (m, 2H), 1.56 – 1.35 (m, 5H), 1.30 – 1.22 (m, 2H). Mass (ESI) $[M-H]^+$: 487.2331, calc: 487.2233.

Synthesis of Fmoc-Lys(racemic exo norbornene)-OH (Scheme S2)

The procedure was the same as the racemic endo norbornene one described above, and the only difference is the starting material, racemic exo 5-norbornene-2-carboxylic acid. 4.8 g product was obtained as white solid. Yield 65%. ^{1}H NMR (400 MHz, $CDCl_3$) δ 7.76 (d, *J* = 7.6 Hz, 2H), 7.61 (t, *J* = 7.4 Hz, 2H), 7.39 (t, *J* = 7.4 Hz, 2H), 7.30 (t, *J* = 7.4 Hz, 2H), 6.13 – 6.07 (m, 1H), 6.07 – 5.97 (m, 2H), 5.92 (d, *J* = 5.9 Hz, 1H), 4.52 – 4.28 (m, 3H), 4.21 (t, *J* = 7.1 Hz, 1H), 3.34 – 3.16 (m, 2H), 2.99 – 2.83 (m, 2H), 2.03 – 1.96 (m, 1H), 1.96 – 1.84 (m, 2H), 1.84 – 1.72 (m, 1H), 1.70 (d, *J* = 8.4 Hz, 1H), 1.61 – 1.49 (m, 2H), 1.49 – 1.37 (m, 2H), 1.37 – 1.23 (m, 2H).Mass (ESI) $[M+H]^+$: 489.2397, calc: 489.2389.

Synthesis of Fmoc-Lys(*S*,*S*,*S* norbornene)-OH (Scheme S3)

(*R*)-4,4-dimethyl-2-oxotetrahydrofuran-3-yl acrylate[5] (compound **3**)

To a stirred solution of (-)-D-pantolactone (13.0 g, 99.9 mmol), TEA (20.8 mL, 150.2 mmol) in DCM (150 mL) at 0 °C was added acryloyl chloride (7.8 mL, 96.6 mmol) dropwise. The resulting mixture was stirred at 0 °C or below for 6 hours. The mixture was poured into 100 mL 1N HCl to quench the reaction. The organic layer was separated and washed with saturated Na_2CO_3, brine, dried over Na_2SO_4 and purified by chromatography on silica gel with hexanes/EA 10:1 ~ 4:1. 9.2 g product was obtained as colorless oil. ^{1}H NMR (400 MHz, $CDCl_3$) δ 6.56 (dd, *J* = 17.3, 1.2 Hz, 1H), 6.25 (dd, *J* = 17.3, 10.4 Hz, 1H), 6.00 (dd, *J* = 10.5, 1.2 Hz, 1H), 5.47 (s, 1H), 4.16 – 4.04 (m, 2H), 1.26 (s, 3H), 1.16 (s, 3H). Mass (ESI) $[M+H]^+$: 185.0801, calc: 185.0727.

(*R*)-4,4-dimethyl-2-oxotetrahydrofuran-3-yl (1*S*,2*S*,4*S*)-bicyclo[2.2.1]hept-5-ene-2-carboxylate[5] (compound **5**)

To a stirred solution of compound 3 from last step (8.9 g, 48.3 mmol) in DCM 80 mL and hexanes 10 ml was added $TiCl_4$ (1.0 M in DCM, 4.8 mL) dropwise at -20 °C. 30 min later, 1,3-cyclopentadiene (4.8 mL, 57.9 mmol) was added dropwise. The reaction mixture was kept at -10 ~ 0 °C for 3 hours before quenched by adding $Na_2CO_3 \cdot 10H_2O$ (5 g) and stirred at 0 °C for 20 min. The resulting mixture was filtered and the filtrate was concentrated in vacuo. The crude product was recrystallized in EA/hexanes. 9 g product was obtained as white crystal. Yield 75%. $[\alpha]_D^{21.4}$ -105.3 (c 3.0, $CHCl_3$). ^{1}H NMR (400 MHz, $CDCl_3$) δ 6.28 (dd, *J* = 5.7, 3.1 Hz, 1H), 5.93 (dd, *J* = 5.7, 2.8 Hz, 1H), 5.35 (s, 1H), 4.08 (d, *J* = 9.0 Hz, 1H), 4.04 (d, *J* = 9.2 Hz, 1H), 3.32 – 3.27 (m, 1H), 3.18 (dt, *J* = 9.2, 4.0 Hz, 1H), 2.98 (s, 1H), 1.97 (ddd, *J* = 12.9, 9.2, 3.7 Hz, 1H), 1.56 – 1.47 (m, 2H), 1.35 (d, *J* = 8.1 Hz, 1H), 1.20 (s, 3H), 1.17 (s, 3H). Mass (ESI) $[M+H]^+$: 251.1194, calc: 251.1205.

(1*S*,2*S*,4*S*)-5-norbornene-2-carboxylic acid[5] (compound **6**)

To a stirred solution of compound **5** (6.5 g, 2.6 mmol) in THF (126 mL) and MeOH (13 mL) was added 5N NaOH (26 mL) dropwise. The resulting mixture was stirred at room temperature for 1 hour and the mixture was concentrated in vacuo (with water bath 35~45 °C) to remove organic solvents. The residue was then placed in a ice bath and 4N HCl was added dropwise to adjust pH to 2-3. The formed white slurry was stirred for 0.5 hour in the ice bath and then filtered. The filter cake was washed with small amount of cold water and dried to give final product as white solid 2.1 g. Yield 60%. $[\alpha]_D^{21}$ -134.0 (c 3.0, $CHCl_3$). ^1H NMR (400 MHz, $CDCl_3$) δ 6.23 (dd, *J* = 5.7, 3.1 Hz, 1H), 6.02 (dd, *J* = 5.7, 2.8 Hz, 1H), 3.26 (s, 1H), 3.02 (dt, *J* = 9.3, 3.9 Hz, 1H), 2.94 (s, 1H), 1.94 (ddd, *J* = 11.8, 9.4, 3.7 Hz, 1H), 1.52 – 1.38 (m, 2H), 1.31 (d, *J* = 8.3 Hz, 1H). Mass (ESI) $[M-H]^+$: 137.0122, calc: 137.0681.

Fmoc-Lys(*S*,*S*,*S* norbornene)-OH (compound **10**)

It was followed the similar protocol for Fmoc-Lys(racemic endo norbornene)-OH (Scheme 1). 5.2 g of final product was obtained as white solid. Yield 68%. $[\alpha]_D^{18.7}$ -64.6 (c 1.0, MeOH). ^1H NMR (400 MHz, DMSO) δ 7.90 (d, *J* = 7.5 Hz, 2H), 7.73 (d, *J* = 7.5 Hz, 2H), 7.56 (t, *J* = 5.5 Hz, 1H), 7.42 (t, *J* = 7.4 Hz, 2H), 7.33 (t, *J* = 7.4 Hz, 2H), 6.08 (dd, *J* = 5.6, 3.0 Hz, 1H), 5.80 (dd, *J* = 5.6, 2.8 Hz, 1H), 4.31 – 4.18 (m, 3H), 3.88 (td, *J* = 8.8, 4.7 Hz, 1H), 3.13 (s, 1H), 3.08 – 2.98 (m, 1H), 2.98 – 2.90 (m, 1H), 2.81 – 2.74 (m, 2H), 1.70 (ddd, *J* = 11.3, 9.3, 3.7 Hz, 2H), 1.65 – 1.56 (m, 1H), 1.41 – 1.17 (m, 8H). Mass (ESI) $[M+H]^+$: 489.3012, calc: 489.2311.

Synthesis of Fmoc-Lys(*R*,*R*,*R* norbornene)-OH (Scheme S4)

The procedure is similar to Fmoc-Lys(*S*,*S*,*S* norbornene)-OH. The auxiliary was exchanged to (+)-L-pantolactone.

(*S*)-4,4-dimethyl-2-oxotetrahydrofuran-3-yl acrylate (compound **3**)

The procedure was similar to D-pantolactone acrylate. Around 10 g product was obtained as colorless oil.). ^1H NMR (400 MHz, $CDCl_3$) δ 6.56 (dd, *J* = 17.3, 1.2 Hz, 1H), 6.25 (dd, *J* = 17.3, 10.5 Hz, 1H), 6.00 (dd, *J* = 10.5, 1.2 Hz, 1H), 5.47 (s, 1H), 4.16 – 4.03 (m, 2H), 1.26 (s, 3H), 1.16 (s, 3H). Mass (ESI) $[M+H]^+$: 185.1911, calc: 185.0736.

(*S*)-4,4-dimethyl-2-oxotetrahydrofuran-3-yl (1S,2S,4S)-bicyclo[2.2.1]hept-5-ene-2-carboxylate (compound **5**)

14.6 g product was obtained after crystallization. Yield 70%. $[\alpha]_D^{20}$ 114.0 (c 3.0, $CHCl_3$). ^1H NMR (400 MHz, $CDCl_3$) δ 6.28 (dd, *J* = 5.7, 3.1 Hz, 1H), 5.93 (dd, *J* = 5.7, 2.8 Hz, 1H), 5.35 (s, 1H), 4.12 – 4.00 (m, 2H), 3.33 – 3.26 (m, 1H), 3.17 (dt, *J* = 9.2, 4.0 Hz, 1H), 3.00 – 2.95 (m, 1H), 1.97 (ddd, *J* = 12.9, 9.2, 3.7 Hz, 1H), 1.56 – 1.44 (m, 2H), 1.38 – 1.31 (m, 1H), 1.20 (s, 3H), 1.17 (s, 3H). Mass (ESI) $[M+H]^+$: 251.2433, calc: 251.1205.

(1*R*,2*R*,4*R*)-5-norbornene-2-carboxylic acid (compound **6**)

4.1 g product was obtained as off white solid. Yield 51%. $[\alpha]_D^{19.2}$ 139.8 (c 1.0, MeOH). ^1H NMR (400 MHz, $CDCl_3$) δ 6.23 (dd, *J* = 5.7, 3.1 Hz, 1H), 6.01 (dd, *J* = 5.7, 2.8 Hz, 1H), 3.25 (s, 1H), 3.01 (dt, *J* = 9.3, 3.9 Hz, 1H), 2.94 (s, 1H), 1.94 (ddd, *J* = 11.8, 9.4, 3.7 Hz, 1H), 1.51 – 1.37 (m, 2H), 1.31 (d, *J* = 8.3 Hz, 1H). Mass (ESI) $[M-H]^+$: 137.0122, calc: 137.0681.

Fmoc-Lys(*R*,*R*,*R* norbornene)-OH (compound **10**)

6.2 g product was obtained as white solid. Yield 81.4%. $[\alpha]_D^{18.6}$ 54.7 (c 1.0, MeOH). ^{1}H NMR (400 MHz, DMSO) δ 7.90 (d, *J* = 7.4 Hz, 2H), 7.74 (d, *J* = 7.3 Hz, 2H), 7.64 – 7.54 (m, 2H), 7.42 (t, *J* = 7.3 Hz, 2H), 7.34 (t, *J* = 7.2 Hz, 2H), 6.12 – 6.05 (m, 1H), 5.84 – 5.79 (m, 1H), 4.31 – 4.21 (m, 3H), 3.92 (s, 1H), 3.13 (s, 2H), 3.03 – 2.95 (m, 2H), 2.81 – 2.73 (m, 2H), 1.75 – 1.64 (m, 2H), 1.64 – 1.57 (m, 1H), 1.45 – 1.10 (m, 8H). Mass (ESI) $[M-H]^+$: 488.9964, calc: 489.2311.

Synthesis of NT-B2 and Biotin-PEG5-NT-B2

The NT-B2 and its analogue Biotin-PEG5-NT-B2 were synthesized on Rink MBHA resin followed standard SPPS protocol. After cleavage in TFA (2.5% H_2O, 2.5% TIPS) the crude products were evacuated by rotavap and re-dissolved in MeOH, which later precipitated in ethyl ether and purified by reverse-phase HPLC (0.1% TFA in H_2O; 0.1% TFA in acetonitrile). MALDI for NT-B2 and Biotin-PEG5-NT-B2 are shown in Figure S58, S65.

Recombinant 6xHis-MBP-tagged MYC Expression and Purification

Human MYC plasmid was prepared in a pVP13-GW expression vector (prepared in-house) with a 6xHis-MBP tag. The plasmid was transformed into BL21(DE3) E. coli cell line and expressed at 37°C for 4hrs in Lysogeny Broth, rotating at 225rpm, after induction of optimal density of 0.6. Protein was extracted from the cells through series of sonication utilizing a sonication probe (QSonica). Purification was first performed using a HisTrap HP 5 mL (Cytiva) column. 6xHis-MBP-tagged MYC was eluted at 20mM Tris, pH7.5; 300mM NaCl; 150mM Imidazole. The sample was then further purified through use of series of size exclusion columns, first with a HiPrep 16/60 S-200 HR (Cytiva) column followed by a HiLoad 16/600 S-200 pg (Cytiva) column. Separation was achieved using 20 mM Tris-HCl, pH 7.5; 100 mM KCl; 0.2 mM EDTA; 20% Glycerol. The obtained 6xHis-MBP-tagged MYC was quantified using nanodrop 280 nm absorbance and corrected with the construct's extinction coefficient.

Generation of binding affinity curves using the recombinant MYC protein

96-well plate (Nunc Immobilizer Nickel-Chelate, Thermo Fisher) was coated with the recombinant 6xHis-MBP-tagged MYC Protein (6.8 µg/mL, 100 µL/well) for 1 hour. Then the plate was washed with PBST ×3 and blocked with peptide (His-His-His-His-His-His, 9 pmol/well) for 1 hour. Later the plate was washed with PBST×3 and incubated with varying concentrations of Biotin-PEG5-NT-B2 (1 nM - 200 µM in 0.1% BSA-PBST) for 1 hour. After washing 3 times with PBST, streptavidin-HRP (200:1) diluted with 0.1% BSA-PBST 200 folds was added to each well (100 µL/well). 20 min later, the solution was dumped, and the plate was washed with PBST×3, followed by addition of chemiluminescent substrate (SuperSignal West Pico Plus Chemiluminescent Substrate, Thermo Scientific) 100 µL/well. The luminescence density of each well was determined by a plate reader (BioTek Synergy H1).

Protein thermal shift assay with Glomelt™

Follow the Biotium's protocol PI-3302133022. The reactions were set up in qPCR tubes, for each tube 1 µg recombinant MYC protein (1 µg/µL, 1 µL), Glomelt™ dye (10x, 2 µL), peptide ligand (2x, 10 uL, final concentration 500 µM), PBS (1x, 7 uL) to make a total volume of 20 µL with triplicate. For control group, PBS (1x, 17 µL) was used without peptide ligand. For qPCR instrument, the ramp rate was set up as 0.02 °C/sec (0.3 °C every 15 sec), temperature range was 37-95 °C. The resulting curve was fitted with a Boltzmann curve within the range of 45-60 °C and Tm was calculated from the fitted curve.

Temperature-dependent circular dichroism spectroscopy

The recombinant MYC protein solution was buffer exchanged into 0.1M of Phosphate Buffer, pH 7.4 (7.4 mL of 1M Na_2HPO_4 + 2.6 of 1M NaH_2PO_4) using 7kD MW Zebra Desalting Column (89882). Following the manufacturer's buffer exchange protocol.

NT-B2R peptide and MYC protein were diluted to the final working concentrations of 100 µM and 0.1 mg/mL respectively. The mixture of both NT-B2R peptide and MYC were prepared with the same final concentrations. The total volume of the samples was brought to 200 µL with 0.1 M of Phosphate Buffer, pH 7.4. Data acquisition was done utilizing a Jasco J-1500 Circular Dichroism Spectrophotometer with the Temperature Interval Scan Measurement function. Temperatures varied from 30 ˚C to 60 ˚C with a 5 ˚C step, holding each temperature point for 20 seconds. Spectra in the 180-280 nm range were recorded in a 1 mm path length cell.

The collected CD spectra were processed in OriginPro™. The set of discrete temperature-dependent spectra was first smoothed by a 10-point weighted average method to improve signal quality. The smoothed set was used to construct a 150x50 virtual matrix with regular gridding, and plotted as a 3D graph with 2D projection. Spectra addition and subtraction were performed before the matrix conversion to generate the calculated and difference spectra.

Cell culture

Cells were cultured in DMEM media containing 10% FBS and 1% PS, at 37°C with 5% CO_2.

Liposome preparation

Equal volumes of DOTAP and DOPE solution (both at 10 mg/mL in chloroform) were mixed and dried under vacuum. The obtained film was rehydrated with either 20 mM HEPES (for empty liposome control), or a peptide solution (200 µM in 20 mM HEPES, for peptide-loaded liposomes). The mixture was sonicated 3 times for 1 minute each to assist the rehydration process. The final concentration of the liposomes was 4 mg/mL. The liposomes were extruded at 60 °C using a polycarbonate membrane with a pore size of 0.2 µm on a mini-extruder (Avanti Polar Lipids, Inc.). The liposome solution was then dialyzed against a 20 mM HEPES solution overnight using a Tube-O-Dialyzer (G-Biosciences).

Liposome loading capacity quantification

200 μL of the liposome solution (4 mg/mL) was dried under vacuum and extracted with 50 μL of DMSO. The obtained solution was analyzed on a reverse-phase HPLC and the corresponding peptide peak was identified by mass spectrometry. The peptide concentration was then determined by comparing the peak area to that of a standard solution. The loading capacities were calculated accordingly.

Cell viability test

Cells were seeded in a 96-well plate at 4 k/well and incubated overnight. Afterward, the cells were washed with complete media and treated with varying concentrations of peptide-loaded liposome or empty liposome. The final media volume was 200 μL in each well. The cells were treated for 48 hours and then washed with fresh media to remove residue peptides or liposomes. For a single well, Resazurin (10 μg/mL in PBS) mixed with 200 μL of fresh cell media was added, and the plate was incubated at 37 °C for 3 hours. The 96-well plate was shaken for 15 min on an orbital mixer in dark, and the fluorescence (540 nm excitation, 590 nm emission) intensities were read by a plate reader.

CETSA[6]

U87 cells were detached by trypsin and resuspended in 5 mL of PBS(1x). The total number of cells was approximately 3.5 million, and cells were centrifuged at 400 g for 5 minutes at 4 °C. The PBS was removed, and the cells were resuspended in 1 mL of fresh PBS. The resuspended cells were transferred to a 1.5 mL centrifuge tube and centrifuged again at 400 g for 5 min at 4 °C. The supernatant was removed, and 300 uL of lysis buffer (1x with protease and phosphatase inhibitors) was added to the cell pellet. The lysed cells were carefully aspirated 10 times, put on ice, and incubated for 30 min on ice. The cell lysate was then centrifuged at 14,000 rpm for 20 min at 4 °C, and the supernatant was collected. The protein concentrations were determined by BCA assay.

To prepare the sample for the cellular thermal shift assay, the quantified cell lysate was adjusted to a final concentration of 1.481 mg/mL with lysis buffer. Then, 195 μL of lysate was mixed with 19.5 μL of peptide (1 mM in 1x PBS) in a PCR tube. For the control group, 19.5 uL PBS (1x) was added. 25 uL of each sample was loaded on PCR tubes with caps on. The PCR tubes were heated on a gradient temperature on a PCR instrument for 3 min with a temperature gradient from 50 to 65 °C. After heating, the sample was cooled at room temperature for 3 min and then centrifuged at 20000 g for 20 min at 4°C.

Next, 18 μL of the resulting supernatant was carefully collected and mixed with 6 μL of XT sample buffer (4x). The sample was then heated to 96 °C for 5 min to denature the protein before running Western blot analysis.

RNA-seq experiments

U87 cells were seeded in six 100x20 mm cell culture dishes at 1000 k/dish and incubated for 24h in full DMEM medium containing 10% FBS and 1% PS. On the next day, empty liposome (55 μg/mL in fresh DMEM medium) were used to replace the old medium for three dishes, and peptide-loaded liposome (0.36 μM peptide, 55 μg/mL lipids in fresh DMEM medium) were used to replace the other three dishes. After overnight incubation in the 5% CO_2 incubator at 37 °C, the cells of each dish were treated with trypsin and harvested for RNA extraction.

Total RNA of the harvested cells was extracted and purified using the RNeasy Micro Kit (QIAGEN). Following the extraction, RNA integrity and quantitation were assessed using the RNA Nano 6000 Assay Kit on the Bioanalyzer 2100 system (Agilent Technologies). A total amount of 1 μg RNA per sample was used as input material for the RNA sample preparations. Poly-A selective RNA-seq libraries were prepared using the NEB NextUltra II kit (New England Biolabs) following manufacturer's recommendations and index codes were added to attribute sequences to each sample. The obtained six library samples were pooled together and sequenced on a NovaSeq 6000 (Illumina) to reach approximately 30M 150bp pair-end reads per sample.

The obtained sequencing data was preprocessed using TrimGalore, aligned using STAR, and counted using HTSeq. Differentially expressed genes were identified using DESeq2. The sequence data and analysis reports are deposited in the GEO repository.

Molecular dynamics (MD) simulation

Before performing MD simulation, the linker charge was computed with Austin Model 1-bond charge corrections (AM1-BCC) methods implemented in AMBER 20 package. After connecting ligand with cyclic peptide, the extra charged were redistributed back to heavy atoms of the linker. The bond, angle, dihedral parameters were edited based on the new covalent bonds forming between peptide and linkers.

All MD simulations were performed by using the AMBER 20 package with GPU acceleration[7]. Force Field ff14sb[8] was used on peptide backbone and the gaff2 force field was used on linker. First, we minimized the hydrogen atoms, amino acid side chain, and the entire peptide system for 500,1000,5000 steps respectively in a general born implicit solvent. All systems were then solvated with TIP3P water with the extension of 15Å from the solute edge. The solvated system contains roughly 12,000 atoms. The water molecules were minimized for 10,000 steps followed by the minimization of the whole system for 20,000 steps until the convergence is reached. Third, the water of the solvated system was equilibrated under constant pressure and temperature (NPT ensemble) for 5-ns then the whole system was equilibrated from 50K to 275K with 25K increments and 100-ps each, and finally at 298K for 800-ps. Production runs were also performed in NPT ensemble for 1-us at 298K using Langevin Thermostat with 2-fs time step. The cutoff of nonbonding interaction which includes Van Der Wall and Electrostatic components was set to 12 Å. The particle Mesh Ewald Method was used to compute long-range electrostatic interaction.

Force Distribution Analysis

Gromacs Force Distribution Analysis (FDA)[9] was used to compute nonbonding forces with Leonard-Jones' potential and Coulomb potential. 12 Å was used for the short-range interaction cutoff. The long-range electrostatic forces are computed with PME. To understand the folded or unfolded conformations, we calculate the sum of pair-wise forces between two loops of the cyclic-peptides by using our in-house script.

Clustering Analysis

In order to identify the dominant cluster of conformations throughout the MD simulations of each cyclic-peptides, cpptraj[10] is employed to perform clustering analysis via the k-means algorithm. Distance between each frame of the trajectories were calculated using backbone Root Mean Square Deviation (RMSD). To decide the optimal numbers of cluster, we compute the Silhouette Score which evaluate the intra-cluster distance. The higher value indicates the conformations is well matched in its own cluster and poorly match in other clusters. Following the clustering analysis, we select the representative conformations that are nearest to the centroid of each cluster for additional analysis.

REFERENCES

(1) Jung, L. A.; Gebhardt, A.; Koelmel, W.; Ade, C. P.; Walz, S.; Kuper, J.; von Eyss, B.; Letschert, S.; Redel, C.; d'Artista, L.; Biankin, A.; Zender, L.; Sauer, M.; Wolf, E.; Evan, G.; Kisker, C.; Eilers, M. OmoMYC blunts promoter invasion by oncogenic MYC to inhibit gene expression characteristic of MYC-dependent tumors. *Oncogene* **2016**, *36*, 1911.

(2) Das, S.; Nag, A.; Liang, J.; Bunck David, N.; Umeda, A.; Farrow, B.; Coppock Matthew, B.; Sarkes Deborah, A.; Finch Amethist, S.; Agnew Heather, D.; Pitram, S.; Lai, B.; Yu Mary, B.; Museth, A. K.; Deyle Kaycie, M.; Lepe, B.; Rodriguez-Rivera Frances, P.; McCarthy, A.; Alvarez-Villalonga, B.; Chen, A.; Heath, J.; Stratis-Cullum Dimitra, N.; Heath James, R. A General Synthetic Approach for Designing Epitope Targeted Macrocyclic Peptide Ligands. *Angewandte Chemie International Edition* **2015**, *54* (45), 13219-13224.

(3) Pederson, R. L.; Fellows, I. M.; Ung, T. A.; Ishihara, H.; Hajela, S. P. Applications of Olefin Cross Metathesis to Commercial Products. *Advanced Synthesis & Catalysis* **2002**, *344* (6-7), 728-735.

(4) Niedermeyer, T. H. J.; Strohalm, M. mMass as a Software Tool for the Annotation of Cyclic Peptide Tandem Mass Spectra. *Plos One* **2012**, *7* (9).

(5) Geng, B.; Guo, L. X.; Lin, B. P.; Keller, P.; Zhang, X. Q.; Sun, Y.; Yang, H. Side chain liquid crystalline polymers with an optically active polynorbornene backbone and achiral mesogenic side groups. *Polym Chem-Uk* **2015**, *6* (29), 5281-5287.

(6) Jafari, R.; Almqvist, H.; Axelsson, H.; Ignatushchenko, M.; Lundback, T.; Nordlund, P.; Molina, D. M. The cellular thermal shift assay for evaluating drug target interactions in cells. *Nature Protocols* **2014**, *9* (9), 2100-2122.

(7) *Amber2020*; 2020.
(8) Maier, J. A.; Martinez, C.; Kasavajhala, K.; Wickstrom, L.; Hauser, K. E.; Simmerling, C. ff14SB: Improving the Accuracy of Protein Side Chain and Backbone Parameters from ff99SB. *J Chem Theory Comput* **2015**, *11* (8), 3696-3713.
(9) Costescu, B. I.; Grater, F. Time-resolved force distribution analysis. *Bmc Biophys* **2013**, *6*.
(10) Roe, D. R.; Cheatham, T. E. PTRAJ and CPPTRAJ: Software for Processing and Analysis of Molecular Dynamics Trajectory Data. *Journal of Chemical Theory and Computation* **2013**, *9* (7), 3084-3095.
